# Supplementary material for: Need for a Standardized Translational Drug Development Platform: Lessons Learned from the Repurposing of Drugs for COVID-19
Source: Microorganisms. 2022 Aug 12;10(8):1639. doi: 10.3390/microorganisms10081639 (PMC9460261; doi:10.3390/microorganisms10081639)
Supplement: Supplementary file 1 [file microorganisms-10-01639-s001.zip › microorganisms-1830682-SM.pdf]

microorganisms

MDPI

Supplementary Material

S1. Experimental details

S1.1. Identification of potential repurposing candidates

To identify compounds and their SARS-CoV-2 antiviral potency and pharmacokinetic data, we performed a literature search on PubMed, Google Scholar, BioRxiv, and MedRxiv. The following search terms were used for in vitro activity data - (COVID-19 or SARS-CoV-2) and (EC50). In addition, the Stanford University Coronavirus Antiviral & Resistance Database (Stanford Coronavirus Antiviral & Resistance Database (CoVDB)), the NIH National Center for Advancing Translational Science database (Data Browser (nih.gov)) were used to identify and extract additional in vitro SARS-CoV-2 efficacy data. For pharmacokinetic data search the terms C_{max} or pharmacokinetics was used in combination with the drug name for drugs with reported anti-SARS-CoV-2 activity. Additional clinical pharmacokinetic data were obtained from the US Food and Drug Administration (FDA) and the European Medicines Agency (EMA) factsheets.

S1.2. In vitro determination of antiviral activity

S1.2.1. In vitro assays using Vero cells and Calu-3 cells

Vero cells were sourced from American Type Culture Collection (ATCC CCL-81) and maintained at 37°C with 5% CO_2 in Dulbecco's Modified Eagle's Medium (DMEM; Welgene), supplemented with 10% heat-inactivated fetal bovine serum (FBS) and 1X Antibiotic-Antimycotic solution (Gibco). Calu-3 cells were sourced from the American Type Culture Collection (ATCC HTB-55) and maintained at 37°C with 5% CO2 in Eagle's Minimum Essential Medium (EMEM, ATCC), supplemented with 20% heat-inactivated fetal bovine serum (FBS), 1X non-essential amino acids (NEAA) and 1X Antibiotic-Antimycotic solution (Gibco).

Vero cells were seeded at 12,000 cells per well in DMEM, supplemented with 2% FBS and 1X Antibiotic-Antimycotic solution (Gibco) in black 384-well, μClear plates (Greiner Bio-One), 24 h prior to the experiment. Calu-3 cells were seeded at 20,000 cells per well in EMEM, supplemented with 20% FBS, 1X nonessential amino acids (NEAA) and 1X Antibiotic-Antimycotic solution (Gibco) in black 384-well, μClear plates (Greiner Bio-One), 24 h prior to the experiment.

SARS-CoV-2 (βCoV/KOR/KCDC03/2020, NCCP43326) was provided by the Korea Disease Control and Prevention Agency (KDCA). Viruses were propagated in Vero cells and viral titers were determined by plaque assays. Each drug was added to the cells prior to the virus infection. Ten-point DRCs were generated, with compound concentrations ranging from 0.05–50 μM. For SARS-CoV-2 infection, plates were transferred into the BSL-3 containment facility and virus was added at a multiplicity of infection (MOI) of 0.0125 for Vero cells or 0.3 for Calu-3 cells.

The infected cells were fixed at 24 hpi for Vero or 48 hpi for Calu-3 in 4% PFA with 0.25% Triton X-100 and scored by immunofluorescence analysis with an antibody specific for the viral N protein of SARS-CoV-2. The confocal microscope images (Perkin Elmer Operetta CLS) were acquired for both viral N protein and cell nuclei then used to generate a dose-response curve (DRC) for each drug. The acquired images were analyzed (Perkin Elmer Columbus software) to quantify cell numbers and infection ratios, and antiviral activity which were normalized to positive (mock no virus with 0.5% DMSO) and negative (virus with 0.5% DMSO) controls in each assay plate. DRCs were fitted by sigmoidal dose-response models, with the following equation: Y = Bottom + (Top - Bottom)/(1 +(IC50/X)Hillslope), using XLfit 5.5 Software or Prism7. IC50 values were calculated from the normalized activity dataset-fitted curves. All IC50 and CC50 values were measured in duplicate, and the quality of each assay was controlled by Z'-factor and the coefficient of variation in percent (%CV).

Pgp-efflux inhibition assays were run using similar protocols including 2 µM (final concentration in well) of CP-100356 (Sigma-Aldrich) added simultaneously to the tested drug.

All experiments using SARS-CoV-2 at Institut Pasteur Korea were performed in compliance with the guidelines of the KNIH, using enhanced Biosafety Level 3 (BSL-3) containment procedures in laboratories approved for use by the KDCA.

S1.2.2. In vitro assays using A549-Dual™ hACE2-TMPRSS2 cells

A549-Dual™ hACE2-TMPRSS2 cells obtained by Invitrogen (Cat. a549d-cov2r) were cultured in DMEM 10% FCS (Hyclone) supplemented with 10 µg/ml blasticidin (Invivigen, ant-bl-05), 100 µg/ml hygromycin (Invivogen, ant-hg-1), 0.5 µg/ml puromycin (Invivogen, ant-pr-1) and 100 µg/ml zeocin (Invivogen, ant-zn-05). For antiviral assay, cells were seeded in assay medium (DMEM 2%) at a density of 15,000 cells/well. One day after, compound was serially diluted in assay medium (DMEM supplemented with 2% v/v FCS) and cells were infected with the SARS-CoV-2-B.1.1.7 (Alpha variant) strain at a MOI of approximately 0.05 $TCID_{50}$/cell. The MOI was kept comparable for the variant strains in the different experiments. On day 4 pi., differences in cell viability caused by virus-induced CPE or by compound-specific side effects were analyzed using MTS as described previously [1].

The results of *in vitro* antiviral experiments were expressed as EC50 values defined as the concentration of compound achieving 50% inhibition of the virus-reduced eGFP signals as compared to the untreated virus-infected control cells.

S1.3. Ex-vivo determination of antiviral activity in Human Airway Epithelia (HAE)

S1.3.1. Ex vivo HAE model – KU Leuven

Viral infection

Bronchial HAEC (catalogue no. EP01MD) from healthy donors were provided by Epithelix company (Geneva, Switzerland) in an air-liquid interphase set-up and treated as described elsewhere [2]. On day 0 of the experiment, the HAEC were first pre-treated for 1 h with basal medium containing compounds, followed by infection with SARS-CoV-2_B.1.1.7 at 5x10^2 $TCID_{50}$/insert virus input in 100 µL MucilAIR medium. After 1.5 hours incubation at 37°C the virus input was removed and at 24 h p.i. an apical wash with MucilAir medium was collected. Every other day from day 0, subsequent apical washes were collected, whereas compound-containing medium in the basolateral side of the H(s)AEC culture was refreshed. Wash fluid was stored at -80°◦C until analysis by RT-qPCR.

RNA extraction and quantitative reverse transcription-PCR (RT-qPCR)

Viral RNA in the apical wash was isolated using the Cells-to-cDNA II cell lysis buffer kit (Thermo Fisher Scientific, catalogue no. AM8723). Briefly, 5 µL wash fluid was added in 50 µL lysis buffer, incubated at room temperature (RT) for 10 min and then at 75◦C for 15 min. 150 µL nuclease-free water was additionally added to the mixture prior to RT-qPCR. Together with the samples, a ten-fold serial dilution of the corresponding virus stock was extracted to later generate a standard curve for the RT-qPCR. Based on this standard curve the amount of viral RNA can be expressed as median Tissue Culture infective dose (TCID50) equivalents per insert (TCID50eq/insert), and the lowest point of the linear part of the standard curve (highest Ct value) determines the lower limit of quantification (LLOQ). The fold reduction of viral load is calculated by dividing the amount of vRNA of an infected-untreated control with the amount of vRNA of the infected-treated sample. The RT-qPCR was performed using iTaq universal probes one-step kit (Bio-Rad, catalogue no. 1725141), and a commercial mix of primers for N gene, manufactured at IDT Technologies (catalogue no. 10006606). The reaction (final volume: 20 µL) consisted of 10 µL one-step reaction mix 2x, 0.5 µL reverse transcriptase, 1.5 µL of primers and probes

mix, 4 µL nuclease-free water, and 4 µL viral RNA. The RT-qPCR was executed on a Lightcycler 96 thermocycler (Roche), starting at 50°C for 15 min and 95°C for 2 min, followed by 45 cycles of 3 sec at 95°C and 30 sec at 55°C.

S1.3.2. Ex vivo HAE model – UVE Marseille

Mucilair™ human airway epithelia (HAE) are reconstituted epithelia composed with human primary cells at low passage (P1) which are fully differentiated and functional. The HAE used in the present study have been reconstituted from primary cells of bronchial biopsies of a 56-year-old Caucasian female donor or a 55-year-old African female donor both with no reported pathologies. HAE were purchased from Epithelix SARL, Geneva, Switzerland [3]. They were maintained at air liquid interface in a serum free specific media (Mucilair media from Epithelix SARL, Geneva, Switzerland). After being washed with pre-warmed OptiMEM medium (Life technologies), human airway epithelia were infected with SARS-CoV-2 at the apical side using a MOI of 0.1, as previously described [3,4]. The SARS-CoV-2 strain used for these studies was derived from a German SARS-CoV-BavPat1/2020 isolate (hCoV-19/Germany/BY-ChVir-929/2020; EPI_ISL_406862; 2020-01-28, kindly provided by C. Drosten, Charité, Berlin, Germany or through European Virus Archive GLOBAL (https://www.european-virus-archive.com/). The virus stock used for this experiment was produced as previously described [3].

Cells were cultivated in a basolateral medium that contained the repurposed drug or remdesivir (positive control) at different concentrations or with no drug (virus control). Each day, medium was renewed, and samples were collected by washing the apical side with 200µL of pre-warmed OptiMEM medium. Infectious titers were determined at day 3 or 4 depending on the experiment by $TCID_{50}$, described below. Experiments with infectious virus were performed in a biosafety level 3 laboratory.

S1.4. In vivo efficacy in hamster

In vivo efficacy of drugs was assessed in two different hamster models of SARS-CoV-2 infection as described below (1.4.1 and 1.4.2). The hamster model of SARS-CoV-2 infection was chosen as the most relevant *in vivo* model for these studies. Indeed, features associated with SARS-CoV-2 infection in Syrian Golden hamsters recapitulate many characteristics found in humans with mild SARS-CoV-2 infections. The presence of viral antigens in nasal mucosa, bronchial epithelial cells and areas of lung have been detected after 2-3 days following intranasal infection that cause inflammation as well as type I interferon dysregulation in both respiratory and non-respiratory tissues including the heart and kidney. This is followed by rapid viral clearance and pneumocyte hyperplasia seven days after inoculation. Viral antigens in epithelial cells of the duodenum, and viral RNA in feces were also detected. In addition, SARS-CoV-2 was transmitted efficiently from inoculated hamsters to naive hamsters by direct contact [5-7].

Although it had shown some positive impact in human, remdesivir was not used as a control in the hamster studies. Its intravenous route of administration and its poor plasma stability in rodent – possibly responsible for its lack of efficacy in hamsters – made it a poor control candidate in this animal model. Indeed, when injected intraperitoneally at a dose of 20.5mg/kg QD from the day of infection to 2 dpi, little significant effect on lung viral RNA yields and surprisingly no significant effect on lung infectious titers were observed in sacrificed animals at 3 dpi. These results are in line with other preclinical investigations using remdesivir as a control compound in rodent studies [8,9]. Sheahan et al. for example showed that "GS-5734 has relatively poor plasma stability in mice (half-life of <5 min) due to the expression of a secreted carboxylesterase 1c (Ces1c) absent in humans" [10]. This is the reason why a genetically modified mouse system (Cesc-/- knock-out) was used for experiments using SARS-CoV; in this system, plasma stability of GS-5734 was markedly increased (half-life of around 25 min). Pruijssers et al. used the same genetically modified mice to show efficacy [11]. It is important to note that the

carboxylesterase 1c (Ces1c) gene is conserved in rat (Rattus norvegicus) and the Syrian hamster (Mesocricetus auratus /Accession number: XM_013119896). Taken together, these data suggest that remdesivir is not a suitable control for Syrian hamster experiments and this explain the lack of data available regarding its efficacy in the hamster model.

S1.4.1. SARS-CoV-2 hamster model - KU Leuven

Hamster and infection

Experimental details of the first hamster infection model were described before [12,13]. Briefly, female Syrian hamsters (Mesocricetus auratus) were purchased from Janvier Laboratories and kept per two in individually ventilated isolator cages (IsoCage N Bio-containment System, Tecniplast) at 21°C, 55% humidity and 12:12 day/night cycles. Housing conditions and experimental procedures were approved by the ethics committee of animal experimentation of KU Leuven (license P065-2020). For infection, female hamsters of 6-8 weeks old were anesthetized with ketamine/xylazine/atropine and inoculated intranasally with 50 µL containing 2x106 $TCID_{50}$ of SARS-CoV-2 Wuhan strain or 104 $TCID_{50}$ of SARS-CoV-2 Beta variant B.1.351 (day 0). On day 4 pi, animals were euthanized for sampling of the lungs and further analysis by i.p. injection of 500 µL Dolethal (200 mg/mL sodium pentobarbital, Vétoquinol SA). All caretakers and technicians were blinded to group allocation in the animal facility.

Treatment regimen

Hamsters (as n=6 per group) were treated by oral gavage (except favipiravir, which was given intraperitoneally, amodiaquine and ivermectin that were given both with oral and subcutaneous administration) with either the vehicle or the drug at selected doses once or twice daily (depending on the determined pharmacokinetic profile determined in hamsters) starting from D0, just before the infection with the selected variant. All the treatments continued until day 3 pi. Hamsters were monitored for appearance, behavior and weight. At day 4 pi, hamsters were euthanized by i.p. injection of 500 µL Dolethal (200mg/mL sodium pentobarbital, Vétoquinol SA). Lungs were collected and viral RNA and infectious virus were quantified by RT-qPCR and end-point virus titration, respectively as described before [14].

Histology

For histological examination, the lungs were fixed overnight in 4% formaldehyde and embedded in paraffin. Tissue sections (5 µm) were analyzed after staining with hematoxylin and eosin and scored blindly for lung damage by an expert pathologist. The scored parameters, to which a cumulative score of 1 to 10 was attributed, were the following: congestion, intra-alveolar hemorrhagic, intra-alveolar edema, apoptotic bodies in bronchus wall, necrotizing bronchiolitis, perivascular edema, bronchopneumonia, perivascular inflammation, peribronchial inflammation and vasculitis.

Sample size calculation

For *in vivo* antiviral efficacy, we want to detect at least 1 log10 reduction in viral RNA levels in treated subjects compared to the untreated, infected control group. Group size was calculated on the independent t-test with an effect size of 2.0 and a power of 80% (effect size = delta mean/SD = 1 log10 decrease in viral RNA/0.5 log10), resulting in 5-6 animals/group. Sample sizes maximized considering limits in BSL3 housing capacity, numbers of animals that can be handled under BSL3 conditions, and availability of compounds.

S1.4.2. SARS-CoV-2 hamster model – UVE Marseille

Hamster

The second model used in these studies involved three-week-old female Syrian hamsters and was previously described in the literature [3,15-18]. Briefly, animals provided by Janvier Labs were weighed/monitored daily and maintained in ISOcage P - Bioexclusion System (Techniplast) with unlimited access to water/food and 14h/10h light/dark cycle. General anesthesia was obtained with isoflurane (Isoflurin®, Axience). Euthanasia, which was also realized under general anesthesia, was performed by cervical dislocation. These experiments were approved by the ethical committee of Marseille (C2EA—14) and the French 'Ministère de l'Enseignement Supérieur, de la Recherche et de l'Innovation' (APAFIS#23975).

Hamster infection and treatment regimen

Four-week-old anesthetized animals (n=6) were intranasally infected with 50µL containing 104 $TCID_{50}$ of SARS-CoV-2 strain BavPat1 (https://www.european-virus-archive.com/; Ref: 026V-03883) in 0.9% sodium chloride solution. Hamsters were orally treated with either of the drugs to be assessed in the appropriate formulation (solution, suspension or emulsion), at the appropriate concentration, prepared from the sourced active principal ingredients (API). The control groups were orally or intranasally inoculated with the corresponding vehicle.

Experimental analysis

Tissue collection, quantitative real-time RT-PCR (RT-qPCR) assays, tissue-culture infectious dose 50 ($TCID_{50}$) assays and histology were all performed as previously described [3,15-17].

Sample size calculation

Group size was calculated with an effect size of 2 and a power of 80%, resulting in 6 animals/group. Sample sizes were maximized within the capacity of the BSL3 housing, and compound and virus stock availability. Animals were randomly assigned to groups but confounders were not controlled. Since the same experimenters carried out infection/treatment/clinical follow-up, it was impossible to perform a blind trial. Blind trials were performed only for the evaluation of lung histopathological changes. Predefined humane endpoints (>20% weight loss, moribund and a scoring >10 calculated according to a clinical evaluation scale) were set as exclusion criteria. No animals were excluded from the study.

S1.5. Graphical representations and statistical analysis

Graphical representations and statistical analyses for both *SARS-CoV-2 hamster models* were performed with Graphpad Prism 7 (Graphpad software). Two-sided statistical analysis was performed using Shapiro–Wilk normality test, Fisher's exact test, Student t-test, Mann–Whitney test, Welch's test, one-way and two-way ANOVA with Post-hoc Dunnett's multiple comparisons test. P-values lower than 0.05 were considered statistically significant. Statistical details for each experiment are described in the figure legends of the corresponding Supplementary Materials. Experimental timelines were created on biorender.com.Pharmacokinetic and Pharmacodynamic studies in hamster

Table S1. Experimental conditions for *in vivo* efficacy studies in SARS-CoV-2 hamster models.

Drug administered	Route of administration	Formulation	Total dose (mg/kg/day)	Frequency/ Duration
Ambroxol hydrochloride	Oral gavage	30% PEG 400,70% MilliQ water	100	BID / 3 days
Amodiaquine	Oral gavage; Sub-cutaneous	1% methylcellulose in water or 10% PEG400 / 5% Tween80 aqueous solution	50; 75; 100	QD / 4 and 5 days

AT-527	Oral gavage	0.5% CMC-Na and 0.5% Tween-80 in water	300; 500	BID / 3 days
Atazanavir (ritonavir-boosted)	Oral gavage	1% citric acid solution (37°C pH 1.7 or pH 2.2)	96 (32)	BID / 3 and 4 days
Camostat mesylate	Oral gavage	100% MilliQ water (pH of the formulation 5.5-6)	200	BID / 4 days
Cepharantine	Oral gavage	30% PEG 400,70% MilliQ water	100	QD / 4 days
Clofazimine	Oral gavage	Corn oil	25	QD / 4 days
Daclatasvir	Oral gavage	0.09% Sodium chloride solution	50	BID / 3 days
Favipiravir	Intraperitoneal	3% sodium bicarbonate in water	600; 925	BID / 3 and 4 days
Fluoxetine hydrochloride	Oral gavage	10% w/v hydroxypropyl-beta-cyclodextrin (HP-β-CD) in water	10; 50; 100	QD / 3 and 4 days
Fluvoxamine maleate	Oral gavage	Physiologic saline acidified with 5% (v/v) solution of hydrochloric acid 1N at a final pH of 4.0	100, 400	QD, BID / 3 and 4 days
Ivermectin	Oral gavage, Subcutaneous	10% PEG400 / 5% Tween80 aqueous solution	0.4	Single dose, QD / 4 days
Molnupiravir	Oral gavage	10% PEG400 and 2.5% Kolliphor-EL in water	150; 300; 400	BID / 3 and 4 days
Nelfinavir	Intraperitoneal	5% DMSO, 95% PBS containing 5% PEG-400 and 5% Tween-80	100	BID / 4 days
Nitazoxanide	Oral gavage	10% [Tween 80, 80% EtOH (70:30 v/v)] and 90% distilled water	500	BID / 3 and 4 days
Nirmatrelvir	Oral gavage	40%PEG400 (v/v) in water	250; 500	BID / 3 and 4 days
Sofosbuvir	Oral gavage	5% ethanol, 55% PEG400, 40% 50 mM citrate buffer (pH 3)	100	QD / 3 days

Table S2. Experimental conditions for pharmacokinetic studies in female Golden Syrian hamster.

Drug administered	Drug assayed	RoA[a]	Food	Formulation	Dose (mg/kg)[b]	Frequency/ duration	PK sampling time points (h)	PK samples per hamster[c]	Hamster total (per dose; replicates)	Matrix
Ambroxol · HCL	Ambroxol	Oral gavage	Fasted	30% PEG 400,70% MilliQ water	3; 30; 100	Single dose	0, 0.5, 1,2,6,12, 24	6	9 (3; triplicate)	Plasma
Amodiaquine	Amodiaquine; N-desethylamodiaquine	Oral gavage; Subcutaneous	Fasted	2 mg/mL in water (pH 3.23), 10 mg/mL in water (pH 3.46)	10; 50	Single dose	0, 0.25, 0.5, 1, 2, 4, 8, 12, 24, 48	9	12 (3; triplicate)	Plasma
AT-527	AT-273	Oral gavage	Fasted	0.5% CMC-Na and 0.5% Tween-80	10; 50; 250	Single dose	0.25, 0.5, 1, 2, 4, 8, 12, 24	8	12 (3; triplicate)	Plasma
Atazanavir (ritonavir-boosted)	Atazanavir	Oral gavage	Fasted	1% citric acid solution (37℃ pH 1.7 or pH 2.2)	24/8; 48/16; 96/32	Single & multiple doses (BID)[d]	0.25, 0.5, 1, 3, 4, 6, 8, 12, 24	5-7	12 (3; triplicate)	Plasma

Drug administered	Drug assayed	RoA[a]	Food	Formulation	Dose (mg/kg) [b]	Frequency/duration	PK sampling time points (h)	PK samples per hamster[c]	Hamster total (per dose; replicates)	Matrix
Cepharantine	Cepharantine	Oral gavage	Fasted	30% PEG 400,70% MilliQ water	3; 30; 100	Single dose	0, 0.5, 1,2,6,12, 24	6	9 (3; triplicate)	Plasma
Clofazimine	Clofazimine	Oral gavage	ad libitum	70:30 Tween20/EtOH mixture (10%) dissolved in Milli-Q water (v/v)	5;25; 125	Single dose	0.5,1,3,6,24, 72,144,240	1	72 (24; triplicate)	Plasma (Lung)
Daclatasvir	Daclatasvir	Oral gavage	Fasted	Saline	1; 5; 25	Single dose	0.25, 0.5, 1, 2, 3, 6, 12, 24	5-7	12 (3; triplicate)	Plasma
Favipiravir	Favipiravir	Oral gavage	Fasted	0.4% Carboxymethyl cellulose in water	241; 481; 753	Single dose and multiple doses (BID) [d]	0.1, 0.25, 0.4, 0.5, 1, 2, 3, 4, 6, 8, 12, 24	5-8	12 (6 for 100 mg/kg QD and 3 for other; triplicate)	Plasma
Fluoxetine · HCL	Fluoxetine; Norfluoxetine	Oral gavage	Fasted	10% w/v hydroxypropyl-beta-cyclodextrin (HP-β-CD) in water	1; 10; 100	Single dose	0.083; 0.25; 0.5; 1; 2; 4; 8; 24; 30; 48	5	18 (6; triplicate)	Plasma (Lung)
Fluvoxamine maleate	Fluvoxamine	Oral gavage	ad libitum	0.9% NaCl (pH 4.06) in water	4; 20; 100	Single dose	0.5, 1,3,6,12, 24	1	54 (18; triplicate)	Plasma (Lung)
Ivermectin	Ivermectin	Oral gavage, Subcutaneous administration	Fasted	DMSO: PEG400: Water (1:7:2)	0.1; 0.4	Single dose	0.25, 0.5, 1, 2, 6, 12, 24	7	12 (3; triplicate)	Plasma
Molnupiravir	EIDD-1931	Oral gavage	ad libitum	10% PEG400 and 2.5% Kolliphor-EL in water	20; 75; 200	Single dose	0.5, 1,2,6,12, 24	1	54 (18; triplicate)	Plasma
Nelfinavir	Nelfinavir	Intraperitoneal administration	Fasted	5% DMSO, 95% PBS containing 5% PEG-400 and 5% Tween-80	10; 50; 50 BID	Single and multiple doses (BID) [d]	0.5, 1, 2, 6, 12, 12.5, 13, 24, 48	6	12 (3 single dose, 6 multiple doses, triplicate)	Plasma
Nitazoxanide	Tizoxanide	Oral gavage	Fasted	10% [Tween 80, 80% EtOH (70:30 v/v)] and 90% distilled water	Nitazoxanide 25; 100; 500 Tizoxanide 100	Single dose	0.25, 0.5, 1, 2, 4, 6, 12	5-7	12 (3; triplicate)	Plasma

Drug administered	Drug assayed	RoA[a]	Food	Formulation	Dose (mg/kg)[b]	Frequency/ duration	PK sampling time points (h)	PK samples per hamster[c]	Hamster total (per dose; replicates)	Matrix
Sofosbuvir	Sofosbuvir; GS-331007	Oral gavage	Fasted	5% ethanol, 55% PEG400, 40% 50 mM citrate buffer (pH 3)	10; 50; 100	Single dose	0.25, 0.5, 1, 2, 4, 8, 12, 24	5-8	12 (6 for 10 mg/kg, 3 for other; triplicate)	Plasma

[a] Route of administration.

[b] Dose (mg/kg) refers to free drug.

[c] excluding pre-dose.

[d] BID dosing (0 and 12h) for one day.

Table S3. Bioanalytical conditions for pharmacokinetic studies in hamster.

Drug assayed	Blood sampling	Sampling volume	Blood thinner	Detection Platform	Sample cleanup	LC Column	Flow [mL/min]	Mobile Phase	Injection Vol. [µL]	LLOQ (ng/mL)
Ambroxol	pricking lateral saphenous vein with a 26G needle	~ 80 µL blood	Heparin	API4000 integrated to Shimadzu LC & CTC-PAL autosampler	Protein precipitation (ACN, 1:6) & aqueous dilution (1:1) techniques	Kinetex Biphenyl, 2.1*30mm	1	A – 0.1% FA in water B/C - 0.1% FA in 40:40:20:MeCN:MeOH:water	10	0.61
Amodiaquine	pricking lateral saphenous vein with a 26G needle	~ 80 µL blood	K_2EDTA	LC-MS/MS - BI_Triple Quad 6500 plus	Protein precipitation (ACN), centrifugation (3220g, for 15+5 min at 4°C) & aqueous dilution (1:1) techniques	ACQUITY UPLC HSS T3 1.8 µm 2.1 × 50 mm	0.65	A:0.1% FA in water; B:0.1% FA in ACN	3	1.0
AT-273	pricking lateral saphenous vein with a 26G needle	~ 80 µL blood	K_2EDTA	LC-MS/MS -AK_Q-Trap 6500	Protein precipitation (ACN/MeOH (25:75, v:v), centrifugation (3220g, for 15+5 min at 4°C) & aqueous dilution (1:1) , process done on ice	ACQUITY UPLC HSS T3 1.8 µm 2.1 × 50 mm	0.65	A:0.1% FA in water; B:0.1% FA in ACN	?	2.0
Atazanavir	pricking lateral saphenous vein	~ 80 µL blood	K_2EDTA	LC-MS/MS -	Protein precipitation (ACN), centrifugation (3220g, for	ACQUITY UPLC HSS	0.6	A:0.1% FA & 2mM HCOONH4 in water/ACN (95:5);	3	1.0

Drug assayed	Blood sampling	Sampling volume	Blood thinner	Detection Platform	Sample cleanup	LC Column	Flow [mL/min]	Mobile Phase	Injection Vol. [µL]	LLOQ (ng/mL)
	with a 26G needle			AU_Triple Quad 6500 Plus	15+5 min at 4°C) & aqueous dilution (1:10 or 1:5)	T3 1.8 µm 2.1 × 50 mm		B:0.1% FA & 2mM HCOONH4 in ACN/water (95:5)		
Cepharantine	pricking lateral saphenous vein with a 26G needle	~ 80 µL blood	Heparin	API4000 integrated to Shimadzu LC & CTC-PAL autosampler	Protein precipitation (ACN, 1:6) & aqueous dilution (1:1) techniques	Kinetex Biphenyl, 2.1*30mm	0.8	A – 0.1% FA in water B/C - 0.1% FA in 40:40:20:MeCN:MeOH:water	20	0.61
Clofazimine	Aorta puncture following inhalation anesthesia	1 mL	K_2EDTA	API-4000-4 MS/MS (quadrupoles)	Centrifugation (3000g for 10 min at 5°C)	ACQUITY UPLC C18-BEH Column, 100Å, 1.7 µm, 2.1 mm X 50 mm	0.25	A: 0.1% Formic acid in UP (ultra-pure water) and B: 0.1% Formic acid in acetonitrile (ACN)	3	2.5
Daclatasvir	pricking lateral saphenous vein with a 26G needle	~ 80 µL blood	K_2EDTA	LC-MS/MS-AU_Triple Quad 6500 Plus	Protein precipitation (ACN), centrifugation (3220g, for 15+5 min at 4°C) & aqueous dilution (1:2 or 1:20)	ACQUITY UPLC HSS T3 1.8 µm 2.1 × 50 mm	0.6	A:0.1% FA & 2mM HCOONH4 in water/CAN (95:5); B:0.1% FA & 2mM HCOONH4 in ACN/water (95:5)	3	1.0
Favipiravir	pricking lateral saphenous vein with a 26G needle	~ 80 µL blood	K_2EDTA	LC-MS/MS-AK_Q-Trap 6500	Protein precipitation (ACN), centrifugation (3220g, for 15+5 min at 4°C) & aqueous dilution (1:5 up to 1:200)	ACQUITY UPLC HSS T3 1.8 µm 2.1 × 50 mm	0.65	A:0.1% FA in water; B:0.1% FA in ACN	4	1.0
Fluoxetine	pricking lateral saphenous vein with a 26G needle	~ 80 µL blood	Heparin	API4500 integrated to Shimadzu LC & CTC-PAL	Protein precipitation (ACN, 1:6) & aqueous dilution (1:1) techniques	YMC Triart, 2.0*30mm	1	A – 0.1% FA in water B/C - 0.1% FA in 40:40:20:MeCN:MeOH:water	20	0.61

Drug assayed	Blood sampling	Sampling volume	Blood thinner	Detection Platform	Sample cleanup	LC Column	Flow [mL/min]	Mobile Phase	Injection Vol. [μL]	LLOQ (ng/mL)
				autosampler						
Fluvoxamine	Aorta puncture following inhalation anesthesia	1 mL	K_2EDTA	API-4000-4 MS/MS (quadrupoles)	Centrifugation (3000g for 10 min at 5°C)	ACQUITY UPLC HSS T3 Column, 100Å, particle size :1.8 μm, 2.1 mm X 50 mm	0.4	A: 0.1% Formic acid in UP (ultra-pure water) and B: acetonitrile (ACN)	4	0.5
Ivermectin	pricking lateral saphenous vein with a 26G needle	~ 80 μL blood	K_2EDTA	LC-MS/MS - AR_Triple Quad 6500 plus	Protein precipitation (ACN), centrifugation (3220g, for 15+5 min at 4°C)	ACQUITY UPLC Protein BEH C4 300Å 1.7 μm 2.1 × 50 mm	0.65	A:0.1% FA & 2mM HCOONH4 in water/ACN (95:5); B:0.1% FA & 2mM HCOONH4 in ACN/water (95:5)	5	1.0
Molnupiravir metabolite (EIDD-1931)	Aorta puncture following inhalation anesthesia	1 mL	K_2EDTA	API-4000-4 MS/MS (quadrupoles)	Centrifugation (3000g for 10 min at 5°C)	ACQUITY UPLC HSS T3 Column, 100Å, particle size :1.8 μm, 2.1 mm X 50 mm	0.4	A: 10 mM ammonium acetate in UP (ultra-pure water) and B: acetonitrile (ACN)	2	1.0
Nelfinavir	pricking lateral saphenous vein with a 26G needle.	~ 80 μL blood	Heparin	API4000 integrated to Shimadzu LC & CTC-PAL autosampler	Protein precipitation (ACN, 1:6) & aqueous dilution (1:1) techniques	YMC Triart, 2.0*30mm	1	A – 0.1% FA in water<br>B/C - 0.1% FA in 40:40:20:MeCN:MeOH:water	20	0.61
Nitazoxanide metabolite (Tizoxanide)	pricking lateral saphenous vein with a 26G needle	~ 80 μL blood	K_2EDTA	LC-MS/MS - AI_Triple Quad 5500	Protein precipitation (ACN), centrifugation (12000 or 3220g, for 15min+3220g for 5 min at 4°C) & aqueous dilution (1:10 or 1:100)	ACQUITY UPLC HSS T3 1.8 μm 2.1 × 50 mm	0.65	A:0.1% FA in water; B:0.1% FA in ACN	3	1.0

Drug assayed	Blood sampling	Sampling volume	Blood thinner	Detection Platform	Sample cleanup	LC Column	Flow [mL/min]	Mobile Phase	Injection Vol. [µL]	LLOQ (ng/mL)
Pentoxifylline	Aorta puncture following inhalation anesthesia	1 mL	K_2EDTA	API-4000-4 MS/MS (quadrupoles)	Centrifugation (3000g for 10 min at 5°C)	ACQUITY UPLC HSS T3 Column, 100Å, particle size :1.8 µm, 2.1 mm X 50 mm	0.4	A: 0.1% Formic acid in UP (ultra-pure water) and B: acetonitrile (ACN)	4	1.25
Sofosbuvir	pricking lateral saphenous vein with a 26G needle	~ 80 µL blood	K_2EDTA	LC-MS/MS - AU_Triple Quad 6500 Plus	Protein precipitation (ACN), centrifugation (3220g, for 15+5 min at 4°C) & aqueous dilution (1:5)	ACQUITY UPLC HSS T3 1.8 µm 2.1 × 50 mm	0.6	A:0.1% FA in water; B:0.1% FA in ACN	4	1.0

Drug concentrations were quantified by HPLC-MS/MS operated in positive ionization mode.,FA, formic acid.; ACN , acetonitril ; MeOH, methanol; LLOQ, lower limit of quantification.

S1.6. Population pharmacokinetic modeling

S1.6.1. Population pharmacokinetic modeling in hamster

For each drug, plasma concentration – time profiles from satellite pharmacokinetic (PK) studies in hamster were pooled and analyzed using a nonlinear mixed-effects modeling approach in NONMEM, v7.4 (Icon Development Solution, Ellicott City, MD, USA). Throughout the model development process, the first-order conditional estimation method with interactions (FOCE-I) was used. Automation and diagnostics were facilitated by the use of Pirana v2.9.9, Pearl-speaks-NONMEM (PsN, v5.2) and R v4.0.3. Concentrations measured to be below the lower limit of quantification (LLOQ) were omitted. The objective function value (OFV), calculated by NONMEM, was used to discriminate between two competing hierarchical models. A significant improvement of a model was indicated by a decrease in OFV of > -3.84 and > -10.84 at a p-value of < 0.05 and < 0.001, respectively (1 degree of freedom difference).

Parent and metabolite (if available) data were analyzed simultaneously. Complete *in vivo* conversion of parent to metabolite was assumed to maintain structural identifiability. One-, two-, and three compartment models were evaluated to describe the structural disposition model for each drug. Different absorption models were explored, including first-order and zero-order absorption models with and without lag time. If sufficient PK data in the absorption phase was available, transit compartment absorption models were evaluated as a more mechanistic description of delayed absorption [19]. For fluoxetine, a model incorporating a first-pass effect was evaluated. Generally, intravenous data in hamster were not available and hence relative bioavailability (F) was fixed to unity. If multiple PK samples per hamster were collected, inter-individual variability (IIV) in PK parameters was included and estimated with an exponential model where appropriate. If only one PK sample per hamster was collected, IIV was fixed to zero and only residual variability was estimated. Residual unexplained variability was implemented as an additive error on the log-transformed observed concentrations. Examples of structural models, including model parameterizations, are provided in the Supplementary Materials (Fig. S18 and S19).

In order to improve the translational aspect of the model, body weight (centered on the median value of each hamster cohort) was implemented a priori as an allometric function on all clearance (exponent 0.75) and volume (exponent 1) parameters. Dose was evaluated as a covariate (centered on the median dose) on all absorption and elimination clearance parameters. If PK data at more than two dose levels was available, exponential and power PK parameter-dose relationships were explored. A categorical function was applied if only two dose levels were tested. The effect of different routes of administration (subcutaneous vs oral) was modelled as a categorical variable. The covariate evaluation was performed stepwise: in the forward step, covariates were included at a statistical significance level of $p = 0.05$ ($\Delta OFV = -3.84$), followed by a more stringent backward elimination step ($p = 0.001$, $\Delta OFV= -10.83$).

Basic goodness-of-fit diagnostics were used to evaluate descriptive performances of the models and to identify potential model misspecifications. Predictive performances of the final models were evaluated by prediction-corrected visual predictive checks (n = 1000). Parameter precision (relative standard errors and 95% confidence intervals) were obtained using the sampling importance resampling procedure [20].

S1.6.2. Population pharmacokinetics in human

For each investigated drug, a literature search was performed to identify available pharmacokinetic information in human [21]. Briefly, if a population pharmacokinetic model was published, the structural PK parameters as well as IIV and covariates were used as reported for simulations (see below). When more than one population pharmacokinetic model was available, the model based on a dense sampling schedule and/or a greater number of participants was selected. If a population PK model was not available, results from a non-compartmental PK analysis were used to derive key PK parameters, namely absorption rate constant (approximated by using the time to peak concentration), elimination clearance and total volume of distribution. To this purpose, a one-compartment disposition model with first order-absorption was assumed. Alternatively, published PK data in human was digitized (WebPlotDigitizer 4.5) and modelled in NONMEM as described for hamster.

S2. Results

S2.1. Selected repurposed drug candidates for further evaluation

Table S4. Summary of literature data for prioritized compounds selected for preclinical assessments.

Compound	SARS-CoV-2 EC_{50} (μM)	Host cell	Drug status (Indication)	References
**Initial list of approved compounds selected**				
Nitazoxanide	1.0	Vero E6	Approved (Diarrhea caused by Giardia lamblia)	[22]
	2.1	Vero E6		[22]
Atazanavir	2.0	Vero E6	Approved (HIV -in combination with other medications)	[23]
	0.2	A549		[23]
	9.36	Vero E6		[24]
	>50	Vero		[25]
Sofosbuvir	>10	Vero	Approved (Chronic hepatitis C -in combination)	[26]
	5.1	HuH-7		[26]
	7.3	Calu-3		[26]
Daclatasvir	0.8	Vero	Approved (Chronic hepatitis C)	[26]
	0.6	HuH-7		[26]

Compound	SARS-CoV-2 EC_{50} (μM)	Host cell	Drug status (Indication)	References
	1.1	Calu-3		[26]
Amodiaquine	1.6	Vero CCL81	Approved (Malaria -in combination with Artesunate)	[27]
	0.6	Huh7.5		[27]
	5.2	Vero		[25]
	>50	Calu-3		[28]
	10	Vero E6		[29]
	4.9	Vero E6		[30]
Favipiravir	>100	Vero E6	Approved (Influenza)	[31]
	446	Vero E6		[17]
	>500	Vero		[25]
	>50	Vero E6		[32]
	>64	VeroE6/TMPRSS2		[33]
	117	Vero E6		[34]
	62	Vero E6		[35]
	>20	Vero		[36]
Ivermectin	2.0		Approved (Intestinal strongyloidiasis and onchocerciasis, head lice and rosacea)	[37]
	1.7	Vero E6		[22]
	2.3	Vero E6		[38]
Clofazimine	0.31	Vero E6	Approved (Leprosy)	[39]
Nelfinavir	0.048	Vero	Approved (HIV -in combination)	[40]
	0.77	VeroE6/TMPRSS2		[33]
	2.89	Vero E6		[41]
Ritonavir	>100	Vero E6	Approved (HIV)	[31]
	>50	Vero E6		[32]
	8.6	VeroE6/TMPRSS2		[40]
**Additional approved or experimental compounds selected**				
AT-527*	0.47 (EC_{90})	HAE	Clinical	[42]
Cepharanthine	0.35	VeroE6/TMPRSS2	Approved (Radiation-induced leukopenia, alopecia areata, and alopecia pityrodes)	[33] [43]
Fluoxetine	1.25	Vero	Approved (Acute and maintenance treatment of Major Depressive Disorder, obsessive Compulsive Disorder, Bulimia Nervosa, acute treatment of Panic Disorder, with or without agoraphobia)	[44]
	0.69	Vero E6		[45]
	0.82	Calu-3		[45]
	6.0	HEK293T-ACE2-TMPRSS2		[46]
Fluvoxamine	10.5	HEK293T-ACE2-TMPRSS2	Approved (Obsessions and compulsions in patients with obsessive compulsive disorder)	[46]
Colchicine	Not reported		Approved (Familial Mediterranean fever and acute gout flares)	Literature source reporting SARS-

Compound	SARS-CoV-2 EC_{50} (μM)	Host cell	Drug status (Indication)	References
				CoV-2 EC_{50} not found
Ambroxol	>30	Vero E6	Approved (Secretolytic therapy, Prophylaxis and treatment of Infant respiratory distress syndrome, Prophylaxis and treatment of postoperative bronchopulmonary complications, Pain relief in acute sore throat)	[47]
Bromhexine	21.7	Vero E6	Approved (Secretolytic therapy, Alteration in the production or elimination of mucus – acute sinusitis, chronic sinusitis, Sjögren's syndrome)	[47]
Camostat	n.a.	Vero	Approved (chronic pancreatitis and postoperative reflux esophagitis)	[48]
	0.083	Calu-3		[48]
Molnupiravir	0.1	Calu-3	Emergency use authorization (EUA) (Treatment of mild-to-moderate coronavirus disease / COVID-19 in adults with positive results of direct SARS-CoV-2 viral testing, and who are at high risk for progression to severe COVID-19, including hospitalization or death, and for whom alternative COVID-19 treatment options authorized by the FDA are not accessible or clinically appropriate)	[49]
Nirmatrelvir		MRC-5	Emergency use authorization (EUA) Paxlovid (nirmatrelvir tablets and ritonavir tablets, co-packaged for oral use) Treatment of mild-to-moderate coronavirus disease (COVID-19) in adults and pediatric patients (12 years of age and older weighing at least 40 kilograms or about 88 pounds) with positive results of direct SARS-CoV-2 testing, and who are at high risk for progression to severe COVID-19, including hospitalization or death)	[50]
Brequinar	0.050	A549-ACE	Clinical	[49]
	>50	Caco-2		[49]
	0.5	Huh7.5		[49]
	0.12	Vero E6		[51]
Thioguanine	2.13	Vero E6	Approved (Acute nonlymphocytic leukemias and chronic phase of chronic myelogenous leukemia)	[52]
Proxalutamide	0.097	LNCaP Cells	Originally preclinical candidate anti-androgen compound developed for oncology Approved in Paraguay for the treatment of COVID-19 in July 2021	[53]
Probenecid	0.75	Vero E6	Approved (Hyperuricemia associated with gout and gouty arthritis)	[54]
	0.0013	NHBE		[54]
	n.a.	Vero E6		[55]
Pentoxifylline	Not reported		Approved (Intermittent claudication caused by chronic occlusive arterial disease of the limbs)	Literature source reporting SARS-CoV-2 EC_{50} not found

*AT-511, free base of AT-527 used.

For a number of compounds antiviral in vivo activity in mice or hamster SARS-CoV-2 infection models has been reported, which was considered during the selection: amodiaquine [29], daclatasvir [56], favipiravir [13,17], molnupiravir [57], mefloquine [58], nelfinavir [58], thioguanine [58], ivermectin [59], clofazimine [39], nirmatrelvir [50], and probenecid [54]. Of note, molnupiravir and nirmatrelvir are novel targeted agents showing clinical efficacy.2.2. In vitro activity for repurposed drug candidates

Table S5. Summary of generated *in vitro* data for prioritized compounds selected for preclinical assessments.

**Compound**	**VERO cells Average IC_{50} (+/- SD) µM**		**Selectivity Index (Vero cells)**		**Calu-3 cells Average IC_{50} (+/- SD) µM**	**SI (Calu-3 cells)**	**A549-Dual™ hACE2-TMPRSS2 cells Average EC_{50} (+/- SD) µM**	**SI (A549 cells)**
	**Without Pgp pump inhibitor**	**With Pgp pump inhibitor**	**Without Pgp pump inhibitor**	**With Pgp pump inhibitor**				
**Bemnifosbuvir (AT-527/AT-511) (experimental cpd)**	**>25**	**>25**	**ND**	ND	>50	ND	>50	ND
Atazanavir	45.5 (+/-7.9)	>50	>2.2	ND	*21	>2.4	24.0 (+/-3.7)	>2.1
Daclatasvir	19.8 (+/-4.1)	*7.6	>2.5	>6.6	*24.4	[T]1.5	>50	[T] <0.1
Favipiravir (FAV)	>50	>50	ND	ND	>50	ND	>100	ND
Molnupiravir	26.0 (+/-11.6)	2.6 (+/-1.2)	>1.9	>19.4	27.8	>1.8	3.6 (+/-1.5)	>13.9
EIDD-1931 (molnupiravir metabolite)	1.5 (+/-0.8)	1.3 (+/-0.4)	>32	>37	3.6 (+/-3.7)	>14	0.47 (+/-0.10)	>106
Nelfinavir	5.5 (+/-2.7)	*1.6	>9	>32	12.8 (+/-1.2)	>3.9	3.2 (+/-0.4)	[T]2.1
Nirmatrelvir	3.4 (+/-1.5)	<0.05 (+/-0.0)	>7.4	>500	0.3 (+/-0.1)	>76	0.11** (+/-0.60)	>2000**
Sofosbuvir	>50	>50	ND	ND	>50	ND	>50	ND
Ambroxol	>50	>50	ND	ND	>50	ND	>50	ND
Amodiaquine	*8.8	*2.6	>5.7	>19	>50	ND	>50	ND
Cepharanthine	4.5	1.9	9.7	17.2	>50	ND	> 50	[T]<0.1
Camostat mesylate	>50	>50	ND	ND	*3.0	>16.9	>50	ND
Clofazimine	5.9 (+/-0.5)	0.2	8.4	208	8.4 (+/-0.06)	3	2.4 (+/-0.43)	[T]2.1
Colchicine	8.4	3.7	[T]2	[T]2.8	[+]NA	[T,+]NA	[+]NA	[T,+]N.A
Fluoxetine	7.7 (+/-4.0)	5.6 (+/-2.4)	3.7	>4.4	21 (+/-5.7)	2.3	> 50	[T]0.1
Fluvoxamine maleate	*26	*32.6	>1.9	>1.5	>50	ND	39.9 (+/-10.1)	>1.2
Ivermectin	4.3 (+/-2.4)	1.1 (+/-0.08)	[T]2.6	[T]5.4	8.9 (+/-4.1)	3.9	[+]NA	[T,+]NA
Mefloquine	*5.7	*2.8	[T]3.6	[T]6.2	13.2 (+/-3.2)	>3.8	>50	[T]<0.1
Nitazoxanide	6.3 (+/-0.5)	3.4 (+/-2.0)	>7.9	>14.7	13.6 (+/-5.3)	3.1	>100	ND

Compound	VERO cells Average IC_{50} (+/- SD) µM		Selectivity Index (Vero cells)		Calu-3 cells Average IC_{50} (+/- SD) µM	SI (Calu-3 cells)	A549-Dual™ hACE2-TMPRSS2 cells Average EC_{50} (+/- SD) µM	SI (A549 cells)
	Without Pgp pump inhibitor	With Pgp pump inhibitor	Without Pgp pump inhibitor	With Pgp pump inhibitor				
Tizoxanide (Nitazoxanide metabolite)	7.2 (+/-0.74)	*2.3	>7	>21.7	14.3 (+/-2.4)	[T]1.4	>100	ND
Pentoxyfilline	>50	>50	ND	ND	>50	ND	>50	ND
Probenecid	>50	>50	ND	ND	>50	ND	>50	ND
Proxalutamide	30.3	21.5	[T]<1.6	[T]<2.3	*14.5	ND	>50	[T]<0.4

**One replicate only; [T]: Toxicity observed; +NA: no sigmoidal curve; ND: not determined; ** Abdelnabi et al., 2022 [60].2.3. Ex vivo activity for repurposed drug candidates*

Table S6. Summary of generated *ex vivo* data for prioritized compounds selected for preclinical assessments.

Compound	Compound concentration (uM)	HAEC/ALI (alpha variant) Log Viral Load Reduction at 4 dpi (Compared to Virus Control and Background)		HAEC/ALI (BavPat variant) Log Viral titer Reduction at 4 dpi (Compared to Virus Control)	
		Average (+/-SD) n>2	Activity	Average (+/-SD) n>2	Activity
**Bemnifosbuvir (AT-527/AT-511) (experimental cpd)**	1	-0.35 (+-1.2)	NO	-0,36 (+-0.85)	NO
	0.3	/	/	-0.38 (+-0.18)	NO
	0.1	/	/	-0.12 (+-0.4)	NO
Atazanavir	10	/	/	0.09 (+-0.64)	NO
Daclatasvir	10	0.17 (+-0.84)	NO	/	/
Favipiravir (FAV)	300	-0.4 (+/-0.2)	NO	/	/
Molnupiravir	10	5.4 (+-1.5)	YES	/	/
	3	1.9 (+-1.3)	[§]YES	-4.62 (+-0)	YES
	1	/	/	-2.37 (+-0.26)	YES
	0.3	/	/	-0.39 (+-0.48)	NO
EIDD-1931 (molnupiravir metabolite)	10	[+]Inc	/	/	/
	3	[+]Inc	/	* -2.89 (+-0.45)	YES
	1	[+]Inc	/	* -0.26 (+-0.69)	NO
	0.3	/	/	* -0.29 (+-0.44)	NO
Nelfinavir	10	3.2 (+-1.4)	YES	/	/
M8 (nelfinavir metabolite)	5	/	/	* -1.56 (+-0.22)	YES
	2.5	/	/	* -0.93(+-0.41)	NO
	1.25	/	/	* -0.68 (+-0.56)	NO
Nirmatrelvir [60]	1	>3.8	YES	/	/
	0.1	1.9 (+-1.6)	[§]YES	/	/
	0.03	-0.30 (+-0.41)	NO	/	/
Sofosbuvir	10	-0.56 (+-0.10)	NO	/	/
Ambroxol	10	* –1.0 (+-0.71)	NO	/	/
Amodiaquine	10	/	/	-0.51 (+-1.60)	NO
Cepharanthine	10	*0.92 (+-0.97)	NO		
Camostat mesylate	10	*>1.7	YES		

Clofazimine	10	/	/	-0.54(+-014)	NO
Colchicine	10	* -0.2 (+-0.44)	NO		
Fluoxetine	10	-0.12 (+-0.10)	NO	0.68 (+-0.11)	NO
Fluvoxamine maleate	10	$^{+}$Inc	/	0.2(+-0.1)	NO
Ivermectin	10	^{T}>4.09	Toxic	/	/
Mefloquine	10	0.45 (+-2.02)	NO	/	/
Nitazoxanide	5	/	/	-1.59 (+-0.72)	Yes
Pentoxyfilline	10	/	/	0.17(+-0.32)	NO
Probenecid	10	0.34 (+-1.0)	NO	/	/
Proxalutamide	10	/	/	0.11 (+-0.6)	NO

**Readout at 3 dpi; $^{+}$Inc: Inconclusive; ^{T}: Toxicity observed; §Variability observed (concentrations likely to be at the efficacy threshold).2.4. Protein binding and in vitro activity for repuposed drug candidates*

Table S7. Protein binding and *in vitro* activity for repurposed drug candidates.

	Protein binding					*in vitro* activity (A549-ACE2TMPRSS2 cells)			
**Drug**	**Fraction unbound in assay medium[a] (%)**	**Fraction unbound in plasma, hamster (%)**	**Fraction unbound in plasma, human (%)**	**Assay**	**Reference, plasma protein binding**	**IC_{50} [uM], medium[b]**	**IC_{50} [uM], unbound[c]**	**IC_{50} [uM], bound, hamster plasma[d]**	**IC_{50} [uM], bound, human plasma[d]**
Ambroxol	95.4	12.5	23.8	ED	DND*i* in-house	>50	>47.4	> 382	>200
Amodiaquine	87.8	9.6	6.7	ED	DND*i* in-house	>50	> 43.9	> 457	>655
Amodiaquine metabolite (N-desethylamodiaquine)	Not available	7.5 (5-10) [e]	7.5 (5–10)	Not specified	Literature [61]	>100	>100	> 108	> 108
AT-273	99.1	95.5	57.8	ED	DND*i* in-house	>50 (AT-511)	> 49.6	>52	>86
Atazanavir (alone)	34.8	20.0	86	ED	DND*i* in-house & literature (human) [62]	24	8.35	42	60
Cepharantine	64.3	1.80	2	ED	DND*i* in-house	>50	>32.2	>1786	>1608
Clofazimine	0.3	<0.1[f]	<0.1[f]	ED	DND*i* in-house	2.37	0.0071	7.11	7.11
Daclatasvir	20.9	5.5	1	ED	DND*i* in-house & literature (human) [63]	>50	>10.5	>190	>1045
Favipiravir	99.3	82.1	46	ED	DND*i* in-house & literature (human) [64]	>100	>99.3	>121	>216
Fluoxetine	68.2	5.1	4.8	ED	DND*i* in-house	>50	>34.1	> 669	>710.4

	Protein binding					in vitro activity (A549-ACE2TMPRSS2 cells)			
**Drug**	**Fraction unbound in assay medium[a] (%)**	**Fraction unbound in plasma, hamster (%)**	**Fraction unbound in plasma, human (%)**	**Assay**	**Reference, plasma protein binding**	**IC_{50} [uM], medium[b]**	**IC_{50} [uM], unbound[c]**	**IC_{50} [uM], bound, hamster plasma[d]**	**IC_{50} [uM], bound, human plasma[d]**
Fluvoxamine	77.1	28.1	25.4	ED	DND*i* in-house	39.9	30.8	> 109	112.1
Ivermectin	Not available	7[e]	7	Not specified	Literature [65]	2.8 (Vero-hSLAM cells, [37])	2.8	40	40
Molnupiravir metabolite (EIDD-1931)	99.7	92.7	92.7[g]	ED	DND*i* in-house	0.47	0.47	0.51	0.51
Nelfinavir	24.3	0.1	0.1	ED	DND*i* in-house	3.2	0.78	778	778
Nirmatrelvir (PF-07321332)	89.8	62.1	45.5	ED	DND*i* in-house	0.025	0.024	0.039	0.044
Nitazoxanide metabolite (Tizoxanide)	Not available	1[e]	1	Not specified	Literature [66]	>100	>100	>10000	>10000
Sofosbuvir	71.6	35 [e]	35	Not specified	DND*i* in-house & literature (human) [67]	>50	> 35.8	>102	>102

ED, equilibrium dialysis.
[a] DND*i* in-house data;
[b] IC_{50} in presence of medium protein (2% serum DMEM media). DND*i* in-house data if not otherwise indicated;.
[c] unbound IC_{50}, that is IC_{50} corrected for binding to medium protein according to: $IC50_{,unbound} = IC50_{medium} \times f_{u, medium;.}$
[d] IC_{50} corrected for protein binding in plasma according to: $IC50_{bound,human} = IC_{50, unbound} / f_{u, plasma, human}$ and $IC50_{bound,hamster} = IC50_{unbound} / f_{u, plasma, hamster}$;.
[e] Protein binding only available for human. It was assumed that species differences in protein binding were negligible;.
[f] Protein binding was reported as 100% (clofazimine). For the calculation of $IC_{50,total}$ 99.9 % plasma protein binding was assumed;.
[g] Protein binding only available for hamster. It was assumed that species differences in protein binding were negligible.

S2.5. Pharmacokinetic profiles of repurposed drug candidates in Golden Syrian hamster

Plasma concentration time profiles of repurposed drug candidates in female Golden Syrian hamster are shown below (for experimental details see Table S2). Briefly, groups of 3 Golden Syrian hamsters were dosed, the average plasma concentration for each time point and each dose group is reported on a logarithmic scale. The error bars correspond to the standard deviation.

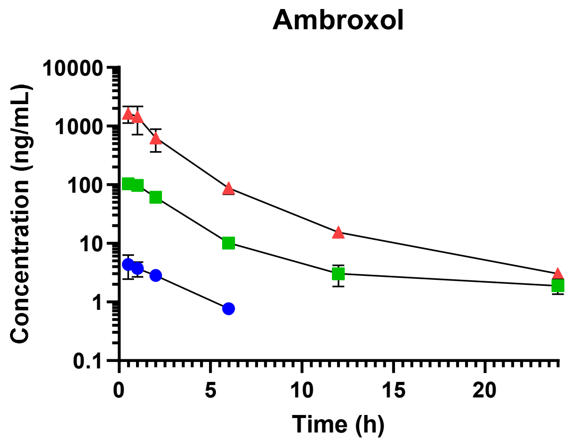

Figure S1. Plasma concentration time profile of ambroxol following oral administration (single dose) of 100 mg/kg (▲), 30 mg/kg (■) and 3 mg/kg (●).

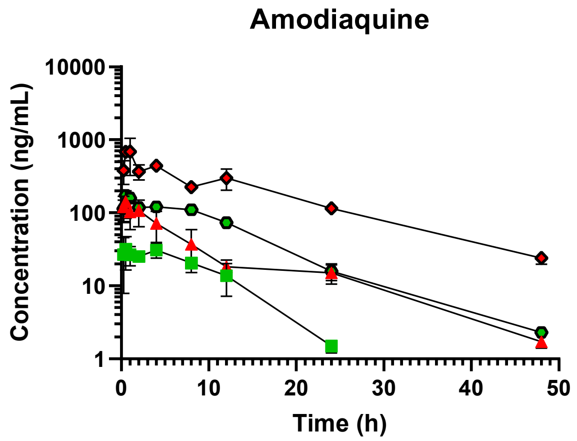

Figure S2. Plasma concentration time profile of amodiaquine following oral administration (single dose) of 50 mg/kg (▲) and 10 mg/kg (■). Plasma concentration time profiles following subcutaneous administration of 50 mg/kg (◆) and 10 mg/kg (⬢).

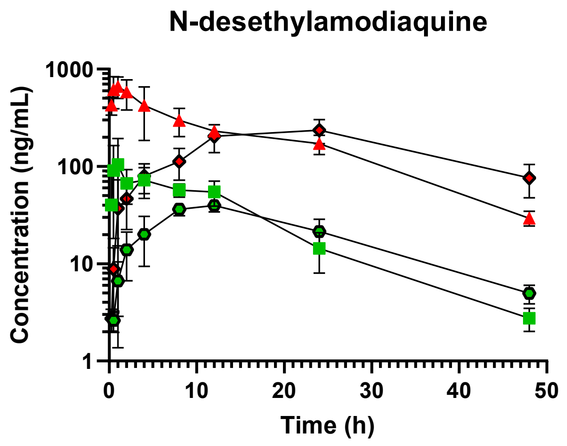

Figure S3. Plasma concentration time profile of N-desethylamodiaquine following oral administration of amodiaquine (single dose) 50 mg/kg (▲) and 10 mg/kg (■). Plasma concentration time profiles following subcutaneous administration of amodiaquine (single dose) 50 mg/kg (◆) and 10 mg/kg (⬢).

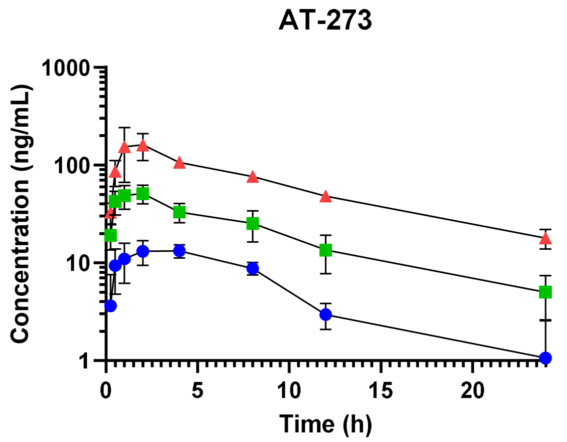

Figure S4. Plasma concentration time profile of a AT-273 following oral administration (single dose) of 250 mg/kg per (▲), 50 mg/kg (■), and 10 mg/kg (●) AT-527.

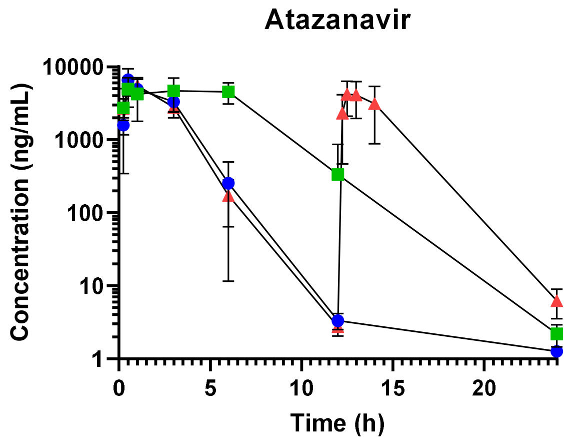

Figure S5. Plasma concentration time profile of atazanavir following oral administration of 48 /16 mg/kg atazanavir/ritonavir at 0 and 12h (▲). Single dose PK profiles following administration of 96/32 mg/kg (■) and 48/16 mg/kg (●) atazanavir/ritonavir.

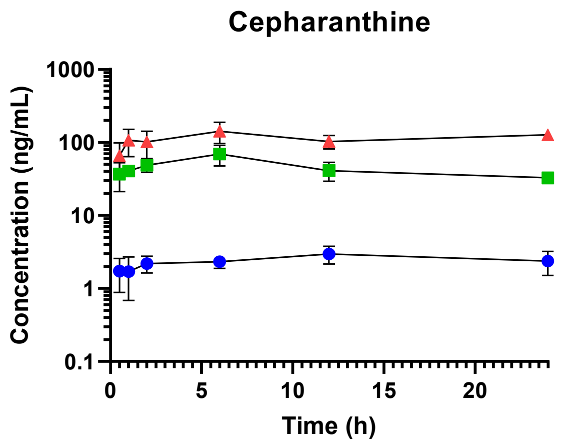

Figure S6. Plasma concentration time profile of cepharantine following oral administration of 100 mg/kg (▲), 30 mg/kg (■) and 3 mg/kg (●).

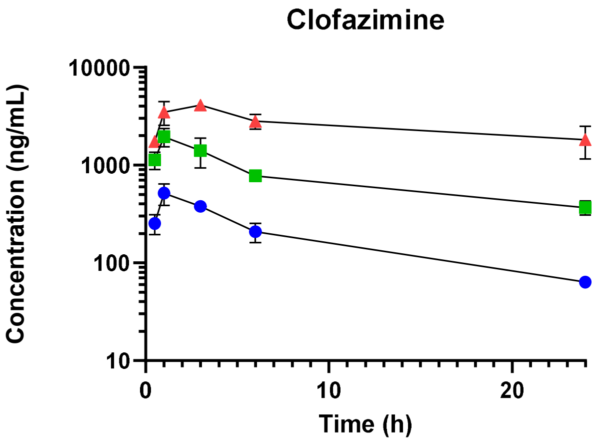

Figure S7. Plasma concentration time profile of clofazimine following oral administration (single dose) of 125 mg/kg per (▲), 25 mg/kg (■), and 5 mg/kg (●).

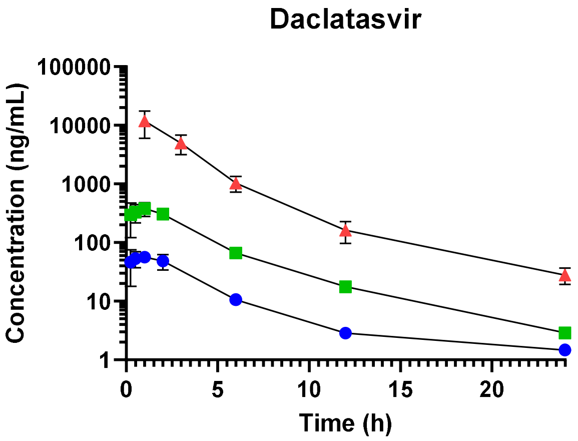

Figure S8. Plasma concentration time profile of daclatasvir following oral administration (single dose) of 25 mg/kg per (▲), 5 mg/kg (■), and 1 mg/kg (●).

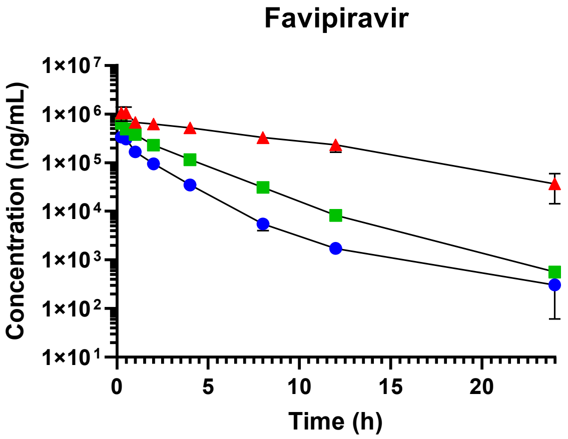

Figure S9. Plasma concentration time profile of favipiravir following intraperitoneal administration (single dose) of 753 mg/kg per (▲), 481 mg/kg (■), and 241 mg/kg (●).

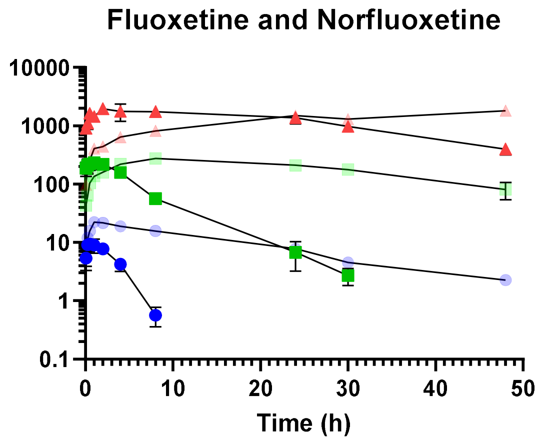

Figure S10. Plasma concentration time profile of fluoxetine / norfluoxetine following PO administration (single dose) of fluoxetine 1 mg/kg (▲/▲), 10 mg/kg (■/■) and 100 mg/kg (●/●).

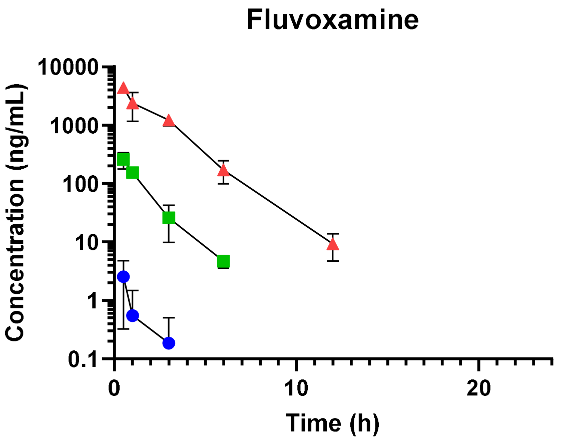

Figure S11. Plasma concentration time profile of fluvoxamine following oral administration (single dose) of fluvoxamine maleate 100 mg/kg per (▲), 20 mg/kg (■), and 4 mg/kg (●).

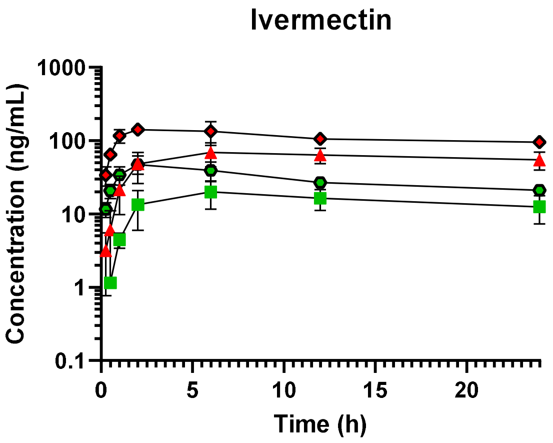

Figure S12. Plasma concentration time profile of ivermectin following oral administration (single dose) of 0.4 mg/kg per (▲), 0.1 mg/kg (■). Plasma concentration time profiles following subcutaneous administration of 0.4 mg/kg (◆) and 0.1 mg/kg (⬢).

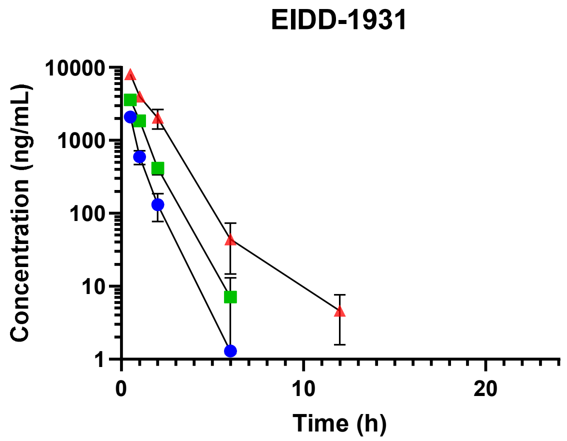

Figure S13. Plasma concentration time profile of EIDD1931 following oral administration of molnupiravir (single dose) 200 mg/kg per (▲), 75 mg/kg (■), and 20 mg/kg (●).

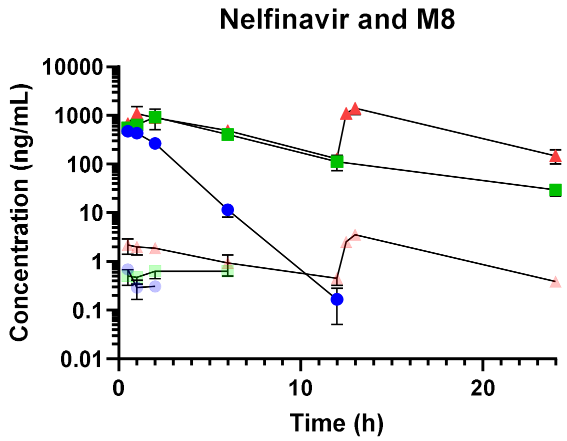

Figure S14. Plasma concentration time profile of nelfinavir / nelfinavir metabolite (M8) following intraperitoneal administration of nelfinavir maleate 10 mg/kg, single dose (▲/▲), 50 mg/kg, single dose (■/■) and 50 mg/kg, BID (●/●).

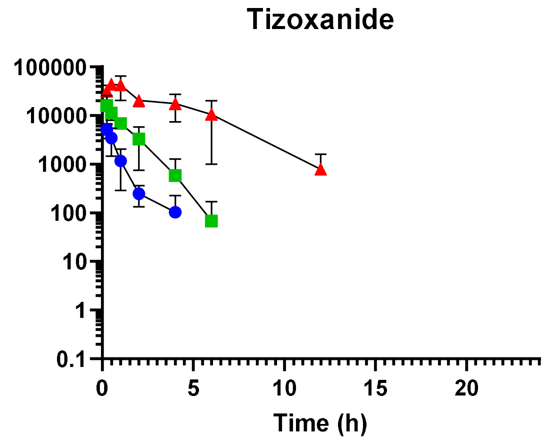

Figure S15. Plasma concentration time profile of tizoxanide following oral administration of nitazoxanide (single dose) 500 mg/kg per (▲), 100 mg/kg (■), and 25 mg/kg (●).

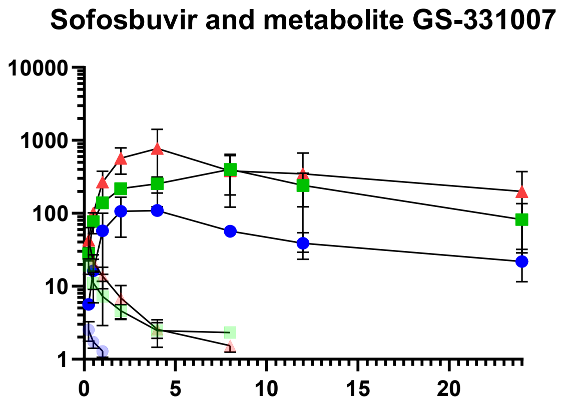

Figure S16. Plasma concentration time profile of sofosbuvir / sofosbuvir metabolite (GS-331007) following oral administration of sofosbuvir 100 mg/kg (▲/▲), 50 mg/kg (■/■) and 10 mg/kg (●/●).

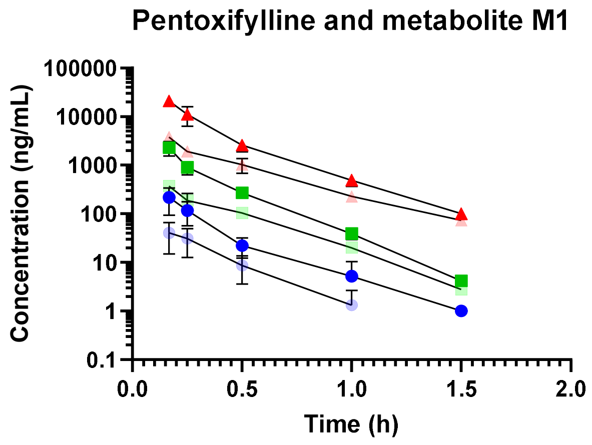

Figure S17. Plasma concentration time profile of pentoxifylline / 1-(5-hydroxyhexyl)-3,7-dimethylxanthine (M1).Oral administration of pentoxifylline 60 mg/kg (▲/▲), 12 mg/kg (■/■) and 2.5 mg/kg (●/●).

S2.6. Pharmacokinetic models in hamster and human

Table S8. Overview of pharmacokinetic models in hamster and human.

Compound	Hamster			Human				Simulation
	Structural PK model	Covariate effects[a]	Table	Structural PK model	Covariate effects	Reference	Table	
Ambroxol	1-cmp, first order absorption and elimination	Allometry Dose on F (power) Dose on CL (power)	S9	2-cmp, transit compartment (n=1) absorption, first order elimination	Allometry	[68]	S10[b]	Fig.S20
Amodiaquine	1-cmp of AQ to 2-cmp of DEAQ, first order absorption and elimination	Allometry Route of administration effect CL_{AQ} and K_A	S11	2-cmp of AQ to 3-cmt of DEAQ, first order absorption and elimination	Allometry, Age on CL_{AQ}, pregnancy on absorption lag-time	[69]	Published model	Fig.S21

Compound	Hamster			Human				Simulation
	**Structural PK model**	**Covariate effects[a]**	**Table**	**Structural PK model**	**Covariate effects**	**Reference**	**Table**	
AT-273	2-cmp, transit compartment absorption (n=1), first order elimination	Saturated absorption model	S12	No human PK data available				Fig.S22
Atazanavir / ritonavir	2-cmp, first order absorption and elimination	Allometry<br>Dose on K_A (exp)	S13	1-cmp, first order absorption and elimination	Sex on CL<br>Body weight on V_C	[70]	Published model	Fig.S23
Cepharantine	1-cmp, first order absorption and elimination	Allometry	S14	3-cmp, first order absorption or infusion, first order elimination	Allometry	[71-73]	S15 [b]	Fig.S24
Clofazimine	3-cmp, first order absorption and elimination	Allometry<br>Dose on K_A (exp)<br>Dose on F (power)	S16	2-cmp, lag time, first order absorption and elimination	Allometry	[74]	Published model	Fig.S25
Daclatasvir	2-cmt, first order absorption and elimination	Allometry	S17	2-cmp, zero-order followed by first order absorption, first order elimination	Body weight, gender, and race on Vc<br>Gender, race, ALT, CrCL on CL	[75]	Published model	Fig.S26
Favipiravir	2-cmp, first order absorption and elimination	Allometry	S18	1-cmp of favipiravir to 1-cmp of M1 metabolite, first order absorption, time-dependent CL	CrCL on CL of M1 metabolite	[76]	Published model	Fig.S27
Fluoxetine	1-cmp fluoxetine, first order absorption and elimination<br>1-cmp norfluoxetine, first pass metabolism, first order elimination	Allometry<br>Dose on CL of fluoxetine (power)<br>Dose on CL of norfluoxetine (power)<br>Dose on FFP (power)	S19	1-cmp fluoxetine, first order absorption and elimination<br>1-cmp norfluoxetine, first pass metabolism, first order elimination	-	[77]	Published model	Fig.S28
Fluvoxamine	1-cmp, first order absorption and elimination	Allometry<br>Dose on F(power)	S20	1-cmp, transit compartment (n=2) absorption, first order elimination	-	[78]	S21 [b]	Fig.S29
Ivermectin	1-cmp, first order absorption and elimination	Allometry<br>Route of administration on F, K_A, and MTT (linear)	S22	2-cmp, transit absorption (n=2), first order elimination	Allometry	[79]	Published model	Fig.S30

Compound	Hamster			Human				Simulation
	Structural PK model	Covariate effects[a]	Table	Structural PK model	Covariate effects	Reference	Table	
Molnupiravir	2-cmp, first order absorption and elimination	Allometry<br>Dose on K_A (exp)	S23	1-cmp, first order absorption and elimination	Allometry<br>Dose on CL (exp)<br>Dose on MTT (power)	[80]	S24[b]	Fig.S31
Nelfinavir	2-cmp, first order absorption and elimination	Allometry<br>Dose on F (linear)<br>Dose on K_A (linear)	S25	1-cmp nelfinavir, lag time, first order absorption and elimination<br>1-cmp M8, linear elimination	Allometry (body weight on the clearance of M8)	[81]	Published model	Fig.S32
Nitazoxanide	2-cmp, first order absorption and elimination	Allometry<br>Dose effect on K_A (power)	S26	1-cmp, first order absorption and elimination	Allometry	[82,83]	Published PBPK model[c]	Fig.S33
Sofosbuvir	2-cmp of SOF to 1-cmp of G007, first order absorption and elimination	Allometry	S27	1-cmp of SOF to 1-cmp of G500 to 2-cmp of G007, first order absorption with first pass metabolism, first order elimination	Sex on CL_{SOF}, food on Ka, CrCL on CL_{G500}, Sex and CrCL on CL_{G007}, Sex on Vc_{G007}, methadone co-administration on Ka_{G007}	[84]	Published model	Fig.S34

Abbreviations: ALT; alanine transaminase, AQ: amodiaquine, CrCL: creatinine clearance, DEAQ: N-desethylamodiaquine, G007: GS-331007, G500: GS-566500, SOF: sofosbuvir

[a] body weight was taken into account by allometric scaling (standardized to median body weight of hamster for the individual studies) on all clearance (exponent fixed to 0.75) and volume (exponent fixed to 1) parameters. No allometric scaling was implemented for AT-273 due to model convergence problems.

[b] data were digitized and modelled using nonlinear mixed-effects modeling in NONMEM.

[c] a one-compartment model was used with pharmacokinetic parameters from an established PBPK model, K_A (0.45 h^{-1}) was assumed in order to generate the mean concentration-time profile with a T_{MAX} at approximately 2 hours, as reported in healthy volunteers.

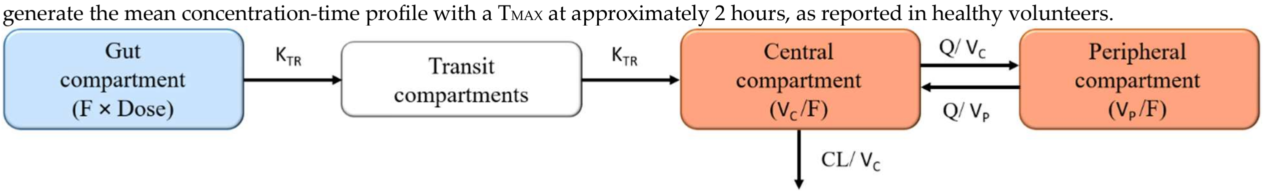

Figure S18. Graphical representation of a 2-compartment disposition model (at the example of ambroxol in human). Absorption from the gut compartment is described by a 1-transit-compartment absorption model. **F** is the relative oral bioavailability. $\mathbf{K_{TR}}$ is the rate constant between absorption compartments. $\mathbf{V_C}$ is the apparent volume of distribution of the central compartment, $\mathbf{V_P}$ is the apparent volume of distribution of the peripheral compartment, **Q** is the inter-compartmental clearance between the central and peripheral compartments, and **CL** is the apparent elimination clearance.

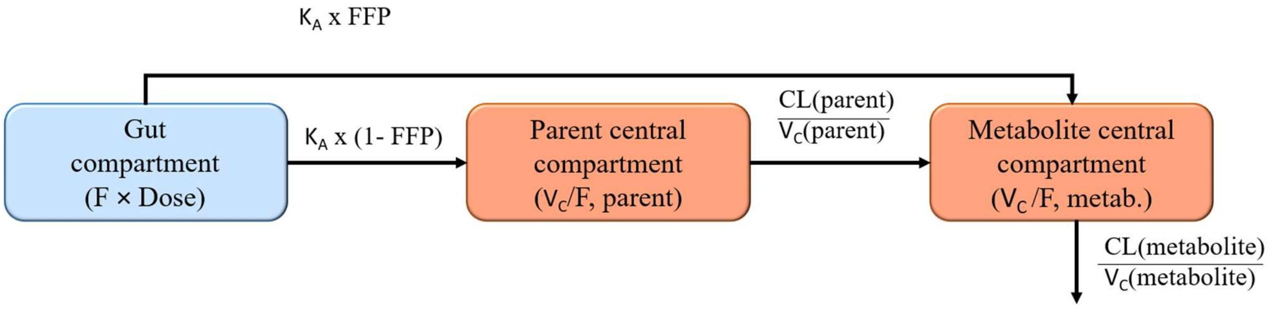

Figure S19. Graphical representation of a 1-compartment disposition model for both parent and metabolite (at the example of fluoxetine and its metabolite norfluoxetine). The model incorporates a first-pass effect where an estimated fraction of the absorbed oral fluoxetine (FFP) was converted into norfluoxetine during the first-pass hepatic metabolism, while the remaining fraction of fluoxetine (1-FFP) was absorbed unchanged. F is the relative oral bioavailability, KA is the first-order absorption rate constant, VC is the apparent central volume of distribution, and CL is the apparent elimination clearance.

Table subscriptions used in the following tables showing pharmacokinetic (PK) parameters for each drug:

[a]Abbreviations used in the following tables showing pharmacokinetic (PK) parameters for each drug.

Pharmacokinetic parameter	Description
F	Relative extravascular bioavailability
K_A	Absorption rate constant
MTT	Mean transit time
FFP	Fraction undergoing first-pass metabolism
CL/F	Elimination clearance
V_C/F	Central volume of distribution
Q/F	Inter-compartment clearance between the central and peripheral compartments
V_P/F	Peripheral volume of distribution
σ	Variance of the residual error
SC	Subcutaneous administration
PO	Oral administration

[b]Population mean parameter estimates and inter-individual variability (IIV) calculated by NONMEM. The coefficient of variation (% CV) for the IIV was calculated as $100\times\sqrt{(\exp(\omega^2)-1)}$.
[c] Relative standard errors (RSE, %) and 95% confidence intervals (95% CIs) were calculated based on sampling importance resampling of the final pharmacokinetic model.

Table S9. Parameter estimates of the final population pharmacokinetic model for ambroxol in hamster.

Parameter	Population estimate (%RSE)	95% CI	IIV, %CV (%RSE) [c]	95% CI
Pharmacokinetics				
F	1 *fixed*	-	32.1 (23.4)	22.3 – 50.1
K_A (h^{-1})	4.76 (15.1%)	3.40 – 6.18	-	-
CL/F (mL/h)	4860 (11.3%)	3815 - 6032	-	-
V_C/F (mL)	12000 (10.9%)	9740 – 14814	-	-
Q/F (mL/h)	1180 (22.5%)	736 – 1795	65.2 (14.3)	44.9 – 87.7

V_P/F (mL)	16100 (31.6%)	8394 - 28559	167 (19.6)	96.3 – 369
σ	0.022 (14%)	0.014 – 0.037		
Covariates				
Dose effect on F (power)	0.689 (11.1%)	0.524 – 0.832	**-**	**-**
Dose effect on CL/F (power)	0.289 (17.7%)	0.202 – 0.400	**-**	**-**

Population estimates are given for a hamster weighting 85g (median weight). Allometric scaling on all clearance (exponent fixed to 0.75) and volume (exponent fixed to 1) parameters.

Table S10. Parameter estimates of the final population pharmacokinetic model for ambroxol in human.

**Parameter**	**Population estimate (%RSE)**	**95% CI**	**IIV, %CV (%RSE)** [c]	**95% CI**
Pharmacokinetics				
F	1 *fixed*	-	-	-
MTT (h)	1.11 (1.8%)	1.07 – 1.15	-	-
CL/F (L/h)	41.4 (3.0%)	38.6 – 43.4	-	-
V_C/F (L)	381 (2.8%)	362 – 404	-	-
Q/F (L/h)	44.5 (7.1%)	39.0 – 51.4	-	-
V_P/F (L)	372 (10.3%)	320 – 466	-	-
σ	0.0005 (18.6%)	0.00032 – 0.00069		

Population estimates are given for an adult weighting 66.6 kg. Allometric scaling on all clearance (exponent fixed to 0.75) and volume (exponent fixed to 1) parameters.

Table S11. Parameter estimates of the final population pharmacokinetic model for amodiaquine (AQ) and N-desethylamodiaquine (DEAQ) in hamster.

**Parameter**	**Population estimate (%RSE)**	**95% CI**	**IIV, %CV (%RSE)** [c]	**95% CI**
Pharmacokinetics				
F	1 *fixed*	-	-	-
K_A (h^{-1})	0.0784 (6.52%)	0.0871 – 0.111	14.5 (12.5%)	12.3 – 18.4
CL/F_{AQ} (mL/h)	4390 (7.44%)	3840 – 5190	11.2 (18.4%)	6.32 – 14.9
V_C/F_{AQ} (mL)	158 (15.3%)	115 – 207	-	-
CL/F_{DEAQ} (mL/h)	804 (9.48%)	675 – 975	28.8 (13.3%)	20.3 – 36.0
V_C/F_{DEAQ} (mL)	1890 (42.2%)	1090 – 4280	802 (15.5%)	294 – 3740
Q_1/F_{DEAQ} (mL/hr)	229 (27.6%)	112 – 364	-	-
V_P/F_{DEAQ} (mL)	3050 (77.1%)	1800 – 9040	-	-
σ_{AQ}	0.144 (15.4%)	0.111 – 0.194		
σ_{DEAQ}	0.0952 (16.5%)	0.0721 – 0.132		
Covariates				
Route effect (SC) on CL/F_{AQ} (%)	-85.3 (1.81%)	(-88.0) – (-82.0)	**-**	**-**
Route effect (SC) on K_A (%)	-20.4 (19.8%)	(-27.9) – (-12.2)	**-**	**-**

Population estimates are given for a hamster weighting 117.18 g (median weight). Allometric scaling on all clearance (exponent fixed to 0.75) and volume (exponent fixed to 1) parameters.

Table S12. Parameter estimates of the final population pharmacokinetic model for AT-273 in hamster.

**Parameter**	**Population estimate (%RSE)**	**95% CI**	**IIV, %CV (%RSE)**
Pharmacokinetics			

F	1 *fixed*	-	41.2 (16.4)
K_{TR} (h^{-1})	12.3 (50.5)	5.25 – 29.0	-
CL/F (mL/h)	5.77 (33.1)	2.11 – 9.23	17.9 (17.3)
V_C/F (mL)	49200 (13.2)	38 200 – 64 500	15.3 (29.9)
Q/F (mL/h)	6730 (12.2)	5 420 – 8 550	-
V_P/F (mL)	84800 (27.3)	572 000 – 1 560 000	-
σ	0.0389 (22.5)	0.0269 – 0.0604	-
Covariates			
K_M (mol)	23.0 (22.2)	14.9 – 34.3	48.6 (18.7)
V_{MAX} (mol/hr)	60.8 (30.5)	40.1 – 114	-

Population estimates are given for a hamster weighting 119 g (median weight). Covariates were added as an enzyme saturation function, i.e. $\frac{d}{dt}A(1) = -\frac{V_{MAX}\cdot A(1)}{(K_M+A(1))}$, where $A(1)$ is amount of drug in gut compartment.

Table S13. Parameter estimates of the final population pharmacokinetic model for atazanavir/ritonavir in hamster.

Parameter	Population estimate (%RSE)	95% CI	IIV, %CV (%RSE) [c]	95% CI
Pharmacokinetics				
F	1 *fixed*	-	25.8 (17.9)	19.3-36.8
K_A (h^{-1})	1.14 (14.9)	0.87-1.54	17.8 (19.2)	12.2-24.9
CL/F (mL/h)	353 (8.51)	299-415	-	-
V_C/F (mL)	435 (11.8)	337-539	**-**	-
Q/F (mL/h)	2.55 (16.8)	1.70-3.31	-	-
V_P/F (mL)	69.4 (42.4)	23.1-134	**-**	-
σ	0.407 (9.06)	0.301-0.584		
Covariates				
Dose effect on K_A (exp)	-0.0222 (18.1)	(-0.0305)-(-0.0142)	**-**	-

Population estimates are given for a hamster weighting 112 g (median weight). Allometric scaling on all clearance (exponent fixed to 0.75) and volume (exponent fixed to 1) parameters.

Table S14. Parameter estimates of the final population pharmacokinetic model for cepharantine in hamster.

Parameter	Population estimate (%RSE)	95% CI	IIV, %CV (%RSE) [c]	95% CI
Pharmacokinetics				
F	1 *fixed*	-	**-**	-
K_A (h^{-1})	1.52 (27.0)	0.91 – 2.53	89.7 (18.1)	51.9 – 135.8
CL/F (mL/h)	804 (25.0)	417 – 1202	-	-
V_C/F (mL)	66400 (12.5)	51540 - 85340	34.6 (11.8)	26.3 – 43.2
σ	0.0507 (11.2)	0.035 – 0.079		

Population estimates are given for a hamster weighting 87g (median weight). Allometric scaling on all clearance (exponent fixed to 0.75) and volume (exponent fixed to 1) parameters.

Table S15. Parameter estimates of the final population pharmacokinetic model for cepharanthine in human.

**Parameter**	**Population estimate (%RSE)**	**95% CI**	**IIV, %CV (%RSE)** [c]	**95% CI**
Pharmacokinetics				
F	0.116 (7.3%)	0.099 – 0.132	-	-
K_A (h^{-1})	0.187 (11.0%)	0.149 – 0.230	-	-
CL/F (L/h)	74.4 (5.5%)	66.5 – 82.5	-	-
V_C/F (L)	110 (10.8%)	85.9 – 132	-	-
Q_1/F (L/h)	258 (7.6%)	226 – 303	-	-
V_{P1}/F (L)	9190 (7.3%)	7888 – 10605	-	-
Q_2/F (L/h)	131 (14.2%)	101 – 171	-	-
V_{P2}/F (L)	438 (26.5%)	315 - 774	-	-
σ	0.102 (7.1%)	0.083 – 0.139		

Data [71-73] was digitized and modeled using nonlinear mixed-effects modeling in NONMEM. All data was included, except for plasma concentration-time data at time < 190 h (poor resolution) following a multiple intravenous dose of 100mg of cepharanthine [73]. Population estimates are given for an adult weighting 70 kg. Allometric scaling on all clearance (exponent fixed to 0.75) and volume (exponent fixed to 1) parameters.

Table S16. Parameter estimates of the final population pharmacokinetic model for clofazimine in hamster.

**Parameter**	**Population estimate (%RSE)**	**95% CI**	**IIV, %CV (%RSE)** [c]	**95% CI**
Pharmacokinetics				
F	1 *fixed*	-	-	-
K_A (h^{-1})	0.38 (11.0)	0.29 – 0.45	-	-
CL/F (mL/h)	49.4 (7.2)	41.9 – 56.0	-	-
V_C/F (mL)	356 (11.9)	287 – 442	-	-
Q_1/F (mL/h)	235 (10.4)	184 – 280	-	-
V_P/F (mL)	2250 (28.0)	1247 – 3740	-	-
Q_2/F (mL/h)	90.5 (20.6)	59.6 – 131	-	-
V_{P2}/F (mL)	7390 (14.9)	5627 – 9707	-	-
σ	0.047 (16.2)	0.037 – 0.068		
Covariates				
Dose effect on K_A (exp)	-0.0094 (20.4)	(-0.0116) – (-0.0071)	-	-
Dose effect on F (power)	-0.109 (16.2)	(-0.153) – (-0.059)	-	-

Population estimates are given for a hamster weighting 120g (median weight). Allometric scaling on all clearance (exponent fixed to 0.75) and volume (exponent fixed to 1) parameters.

Table S17. Parameter estimates of the final population pharmacokinetic model for daclatasivir in hamster.

**Parameter**	**Population estimate (%RSE)**	**95% CI**	**IIV, %CV (%RSE)** [c]	**95% CI**
Pharmacokinetics				
F	1 *fixed*	-	89 (15.1)	56.2 – 127
K_A (h^{-1})	0.415 (5.84)	0.372 – 0.465	-	-
CL/F (mL/h)	239 (19.6)	166 – 343	21.7 (21.3)	10.5 – 30.0
V_C/F (mL)	86.3 (27.0)	46.1 – 135	79 (13.1)	52.1 – 104
Q_1/F (mL/hr)	33.4 (25.5)	19.0 – 51.3	80.4 (14.8)	48.9 – 110
V_P/F (mL)	438 (25.6)	283 – 754	-	-
σ	0.0436 (25.3)	0.0286 – 0.0702		

Population estimates are given for a hamster weighting 109.94 g (median weight). Allometric scaling on all clearance (exponent fixed to 0.75) and volume (exponent fixed to 1) parameters.

Table S18. Parameter estimates of the final population pharmacokinetic model for favipiravir in hamster.

**Parameter**	**Population estimate (%RSE)**	**95% CI**	**IIV, %CV (%RSE)** [c]	**95% CI**
Pharmacokinetics				
K_A (h^{-1})	0.774 (7.71)	0.662 – 0.905	23.7 (11.6)	17.4 – 40.3
CL/F (mL/h)	58.2 (6.06)	51.6 – 65.7	17.6 (29.7)	13.7 – 21.8
V_C/F (mL)	11.7 (14.7)	8.56 – 15.3	-	-
Q_1/F (mL/hr)	1.80 (12.7)	1.43 – 2.28	-	-
V_P/F (mL)	11.6 (8.7)	9.91 – 13.8	-	-
σ	0.0503 (21.2)	0.0370 – 0.0776		

Population estimates are given for a hamster weighting 109.32 g (median weight). Allometric scaling on all clearance (exponent fixed to 0.75) and volume (exponent fixed to 1) parameters.

Table S19. Parameter estimates of the final population pharmacokinetic model for fluoxetine in hamster.

**Parameter**	**Population estimate (%RSE)**	**95% CI**	**IIV, %CV (%RSE)** [c]	**95% CI**
Pharmacokinetics (fluoxetine)				
F	1 fixed	-	17.8 (38.8)	10.8 – 21.5
K_A (h-1)	5.34 (11.7)	4.24 – 6.68	38.5 (40.1)	24.3 – 55.9
CL/F (mL/h)	447 (7.0)	392 – 515	20.2 (45.2)	11.46 – 26.16
V_C/F (mL)	3530 (6.5)	3114 – 3994	-	-
Σ	0.0585 (18.2)	0.0399 – 0.0825	-	-
Pharmacokinetics (norfluoxetine)				
FFP	0.323 (11)	0.256 – 0.395	43.3 (38.9)	30.2 – 64.2
CL/F (mL/h)	93.3 (6.3)	81.6 – 104	10.7 (51.8)	5.47 – 16.1
V_C/F (mL)	2740 (5.7)	2459 – 3059	-	-
σ	0.0174 (18.9)	0.0118 – 0.0249		
Covariates				
Dose effect on FFP (power)	-0.0175 (13.7)	(-0.022) – (-0.012)	-	-

Dose effect on CL/F fluoxetine (power)	-0.511 (5.5)	(-0.561) – (-0.453)	-	-
Dose effect on CL/F norfluoxetine (power)	-0.265 (13)	(-0.337) – (-0.200)	-	-

Population estimates are given for a hamster weighting 97 g (median weight). Allometric scaling on all clearance (exponent fixed to 0.75) and volume (exponent fixed to 1) parameters.

Table S20. Parameter estimates of the final population pharmacokinetic model for fluvoxamine in hamster.

**Parameter**	**Population estimate (%RSE)**	**95% CI**	**IIV, %CV (%RSE)** [c]	**95% CI**
Pharmacokinetics				
F	1 *fixed*	-	-	-
K_A (h^{-1})	4.5 (fixed)	-	-	-
CL/F (mL/h)	9500 (10.8)	7753 - 11644	-	-
V_C/F (mL)	16500 (12.8)	12920 - 21118	-	-
σ	0.257 (27.4)	0.175 - 0.451		
Covariates				
Dose effect on F (power)	1.21 (7.4)	1.04 – 1.38	-	-

Population estimates are given for a hamster weighting 131 g (median weight). Allometric scaling on all clearance (exponent fixed to 0.75) and volume (exponent fixed to 1) parameters.

Table S21. Parameter estimates of the final population pharmacokinetic model for fluvoxamine in human.

**Parameter**	**Population estimate (%RSE)**	**95% CI**	**IIV, %CV (%RSE)** [c]	**95% CI**
Pharmacokinetics				
F	1 *fixed*	-	-	-
MTT (h)	3.37 (3.5)	3.15 – 3.16	-	-
CL/F (L/h)	0.148 (3.0)	0.139 – 0.157	-	-
V_C/F (L)	2.44 (4.6)	2.23 – 2.67	-	-
σ	0.0089 (51.7)	0.0055 - 0.0233		

Population estimates are given for an adult weighting 79 kg (median weight). Allometric scaling on all clearance (exponent fixed to 0.75) and volume (exponent fixed to 1) parameters.

Table S22. Parameter estimates of the final population pharmacokinetic model for ivermectin in hamster.

**Parameter**	**Population estimate (%RSE)**	**95% CI**	**IIV, %CV (%RSE)** [c]	**95% CI**
Pharmacokinetics				
F	1 *fixed*	-	-	-
K_A (h^{-1})	0.531 (8.35)	0.404-0.730	-	-

MTT (h)	0.478 (7.42)	0.329-0.622	53.4 (17.4)	36.6-76.2
CL/F (mL/h)	13.3 (0.160)	10.3-16.6	-	-
V_C/F (mL)	543 (5.48)	450-663	27.8 (12.6)	18.9-33.5
σ	0.0317 (9.67)	0.0228-0.0463		
Covariates				
Effect of SC administration on F	0.656 (15.7)	0.356-0.967	-	-
Effect of SC administration on K_A	2.05 (50.0)	1.23-3.18	-	-
Effect of SC administration on MTT	-0.85 (5.61)	(-0.91) - (-0.71)	-	-

Population estimates are given for a hamster weighting 120 g (median weight). Allometric scaling on all clearance (exponent fixed to 0.75) and volume (exponent fixed to 1) parameters. Effect of SC administration was compared to PO administration.

Table S23. Parameter estimates of the final population pharmacokinetic model for molnupiravir in hamster.

**Parameter**	**Population estimate (%RSE)**	**95% CI**	**IIV, %CV (%RSE)** [c]	**95% CI**
Pharmacokinetics				
F	1 *fixed*	-	-	-
K_A (h^{-1})	1.45 (7.5)	1.27-1.69	-	-
CL/F (mL/h)	1340 (7.6)	1157 – 1533	-	-
V_C/F (mL)	172 (21.6)	81.1 – 229	-	-
Q_1/F (mL/h)	15.8 (23.1)	9.42 – 23.6	-	-
V_P/F (mL)	75.3 (23.0)	52.0 - 118	-	-
σ	0.163 (8.1)	0.119 – 0.220		
Covariates				
Dose effect on K_A (exp)	-0.00247 (23.7)	(-0.0036) – (-0.0014)	-	-

Population estimates are given for a hamster weighting 114 g (median weight). Allometric scaling on all clearance (exponent fixed to 0.75) and volume (exponent fixed to 1) parameters.

Table S24. Parameter estimates of the final population pharmacokinetic model for molnupiravir in human.

**Parameter**	**Population estimate (%RSE)**	**95% CI**	**IIV, %CV (%RSE)** [c]	**95% CI**
Pharmacokinetics				
F	1 *fixed*	-	-	-
MTT (h)	1.0 (3.3%)	0.94 – 1.07	-	-
CL/F (L/h)	80.9 (3.9%)	74.4 – 86.4	-	-
V_C/F (L)	128 (5.7%)	115 – 142	-	-
σ	0.136 (7.9%)	0.106 – 0.189		
Covariates				
Dose effect on CL/F (exp)	-0.00021 (16.2%)	(-0.00028) – (-0.00015)	-	-

Dose effect on MTT (power)	0.127 (10.8%)	0.098 – 0.153	-	-

Population estimates are given for an adult weighting 75 kg (median weight). Allometric scaling on all clearance (exponent fixed to 0.75) and volume (exponent fixed to 1) parameters. For model development, only data from administration of molnupiravir as a capsule and on the first day of dosing (SAD and MAD study) was included.

Table S25. Parameter estimates of the final population pharmacokinetic model for nelfinavir in hamster.

Parameter	Population estimate (%RSE)	95% CI	IIV, %CV (%RSE) [c]	95% CI
Pharmacokinetics				
F	1 *fixed*	-	-	-
K_A (h^{-1})	1.25 (20.7)	0.90 – 1.94	9.6 (121)	1.37 – 20.4
CL/F (mL/h)	613 (4.2)	569 – 665	-	-
V_C/F (mL)	547 (15.3)	394 – 715	-	-
Q/F (mL/h)	130 (13.3)	100 – 166	-	-
V_P/F (mL)	2980 (31.1)	2071 – 5509	-	-
σ	0.0484 (21.5)	0.0364 – 0.0763	-	-
Covariates				
Dose effect on K_A (linear)	-0.801 (3.7)	(-0.859) – (-0.743)	-	-
Dose effect on F (linear)	0.384 (32)	0.172 – 0.638	-	-

Population estimates are given for a hamster weighting 80 g (median weight). Allometric scaling on all clearance (exponent fixed to 0.75) and volume (exponent fixed to 1) parameters.

Table S26. Parameter estimates of the final population pharmacokinetic model for nitazoxanide in hamster.

Parameter	Population estimate (%RSE)	95% CI	IIV, %CV (%RSE) [c]	95% CI
Pharmacokinetics				
F	1 *fixed*	-	-	-
K_A (h^{-1})	2.24 (29.6)	1.41-3.96	33.4 (28.7)	16.2-55.0
CL/F (mL/h)	626 (11.9)	492-771	43.2 (18.1)	30.2-61.3
V_C/F (mL)	188 (38.6)	46.9-337	-	-
Q/F (mL/h)	375 40.7)	215-784	58.8 (31.3)	29.9-111
V_P/F (mL)	243 (17.9)	166-337	-	-
σ	0.145 (9.49)	0.104-0.210	-	-
Covariates				
Dose effect on K_A (power)	-0.767 (17.1)	(-1.022) – (-0.531)	-	-

Population estimates are given for a hamster weighting 120 g (median weight). Allometric scaling on all clearance (exponent fixed to 0.75) and volume (exponent fixed to 1) parameters.

Table S27. Parameter estimates of the final population pharmacokinetic model for sofosbuvir (SOF) and GS-331007 (G007) in hamster.

**Parameter**	**Population estimate (%RSE)**	**95% CI**	**IIV, %CV (%RSE)** [c]	**95% CI**
Pharmacokinetics				
F	1 *fixed*	-	11.5 (19.2)	5.65 – 14.7
K_A (h^{-1})	0.622 (15.9)	0.478 – 0.861	-	-
CL/F_{SOF} (mL/h)	122000 (14.4)	90300 – 157000	-	-
V_C/F_{SOF} (mL)	46600 (29.9)	25300 – 81200	-	-
Q_1/F_{SOF} (mL/h)	139000 (17.2)	96300 – 188000	-	-
V_P/F_{SOF} (mL)	1490000 (27.9)	888000 – 2460000	-	-
CL/F_{G007} (mL/hr)	390 (17.4)	249 – 510	44.8 (16.8)	29.4 – 58.2
V_C/ F_{G007} (mL)	2750 (17.0)	1890 – 3660	20.7 (26.9)	10.1 – 29.5
σ_{SOF}	0.226 (22.6)	0.154 – 0.354		
σ_{G007}	0.148 (21.3)	0.096 – 0.222		

Population estimates are given for a hamster weighting 113.11 g (median weight). Allometric scaling on all clearance (exponent fixed to 0.75) and volume (exponent fixed to 1) parameters.2.7. Simulated pharmacokinetic parameters of repurposed drugs hamster and human

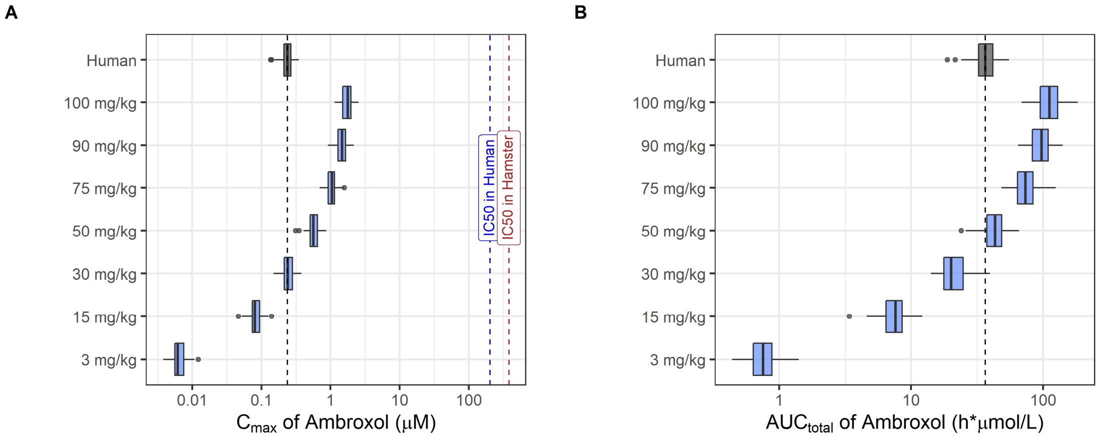

Figure S20. Simulated pharmacokinetic parameters of ambroxol in human and hamster. Predicted pharmacokinetic parameters, i.e., C_{max} (**A**) and AUC_{total} (**B**), in human receiving 30 mg of ambroxol twice daily for 10 days (grey box) were compared with pharmacokinetic parameters of hamster receiving 3 to 100 mg/kg of ambroxol twice daily (blue boxes) for same number of days. Boxes and whiskers represent the median with inter-quantile range and the 95% prediction intervals, respectively. Vertical lines denote the IC_{50} (A549-ACE2TMPRSS2 cells), corrected for plasma protein binding in hamster (red line) and human (blue line).

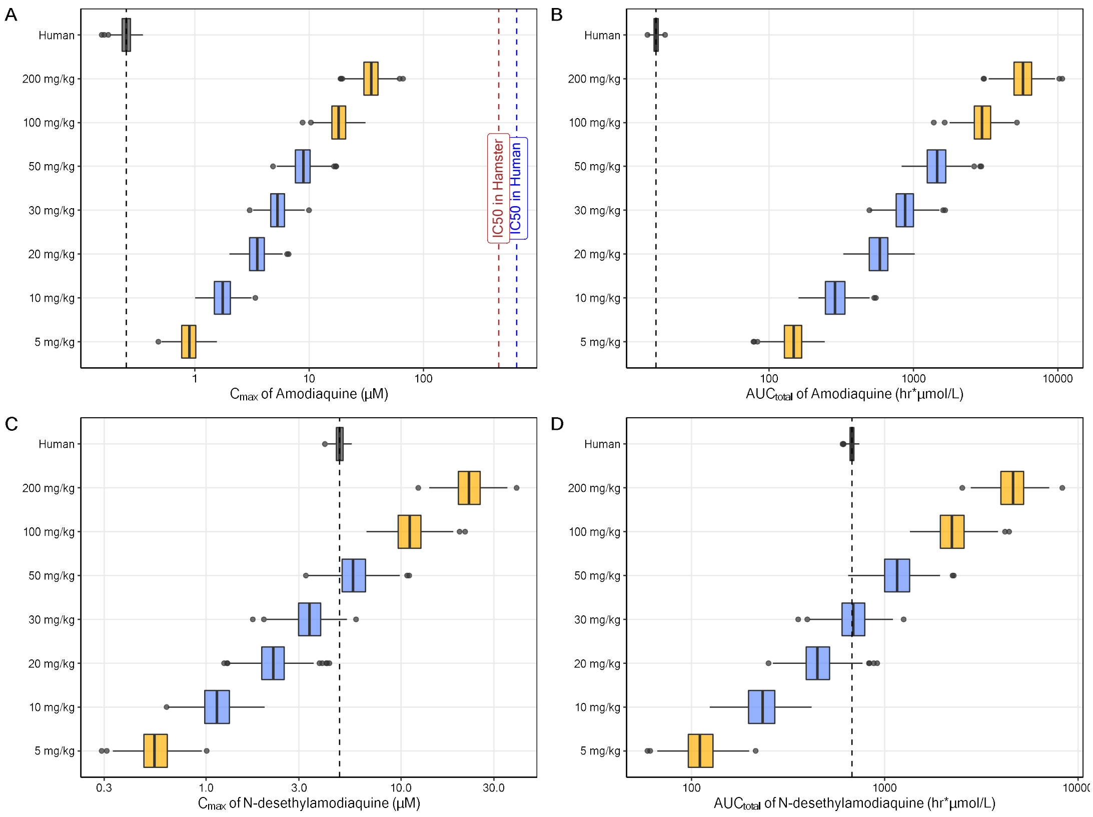

Figure S21. Simulated pharmacokinetic parameters for amodiaquine and N-desethylamodiaquine in human and hamster. Predicted pharmacokinetic parameters, i.e. C_{max} of amodiaquine (**A**) and AUC_{total} of amodiaquine (**B**), C_{max} of N-desethylamodiaquine (**C**) and AUC_{total} of N-desethylamodiaquine (**D**), in human associated with receiving 540 mg of amodiaquine once daily for 10 days (grey box) were compared with pharmacokinetic parameters of hamster receiving 5 to 200 mg/kg amodiaquine twice daily (coloured boxes, yellow box represents the extrapolation beyond the doses tested in satellite PK studies in hamsters). Boxes and whiskers represent the median with inter-quantile range and the 95% prediction intervals, respectively. Vertical lines denote the IC_{50} (A549-ACE2TMPRSS2 cells), corrected for plasma protein binding in hamster (red line) and human (blue line).

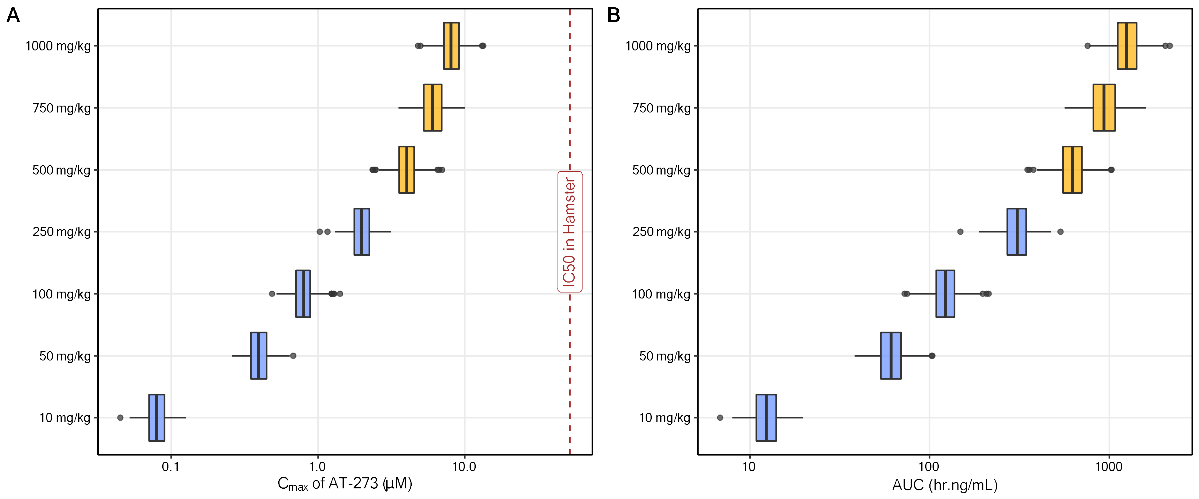

Figure S22. Simulated pharmacokinetic parameters of AT-273 in hamster. Predicted pharmacokinetic parameters, i.e., C_{max} (**A**) and AUC_{total} (**B**), in hamster receiving 10 to 1000 mg/kg of AT-527

twice daily (blue boxes) for 10 days of dosing. Boxes and whiskers represent the median with inter-quantile range and the 95% prediction intervals, respectively. Vertical lines denote the IC_{50} (for AT-511 in A549-ACE2TMPRSS2 cells), corrected for plasma protein binding in hamster (red line).

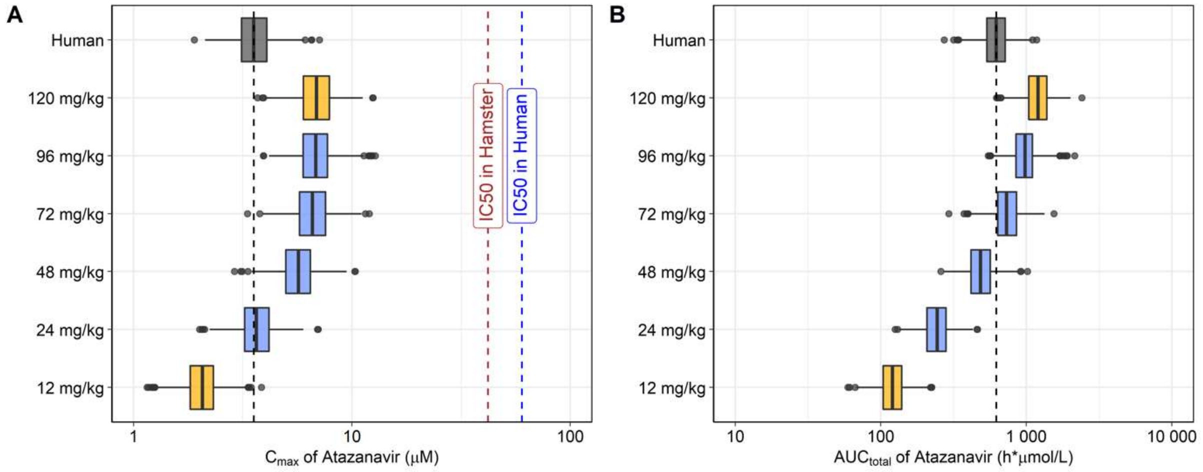

Figure S23. Simulated pharmacokinetic parameters for atazanavir in human and hamster. Predicted pharmacokinetic parameters, i.e. C_{max} (**A**) and AUC_{total} (**B**), in human associated with receiving 300 mg of atazanavir once daily for 10 days (grey box) were compared with pharmacokinetic parameters of hamster receiving 12 up to 120 mg/kg atazanavir twice daily (coloured boxes, yellow box represents the extrapolation beyond the doses tested in satellite PK studies in hamsters). Boxes and whiskers represent the median with inter-quantile range and the 95% prediction intervals, respectively. Vertical lines denote the IC_{50} (A549-ACE2TMPRSS2 cells), corrected for plasma protein binding in hamster (red line) and human (blue line).

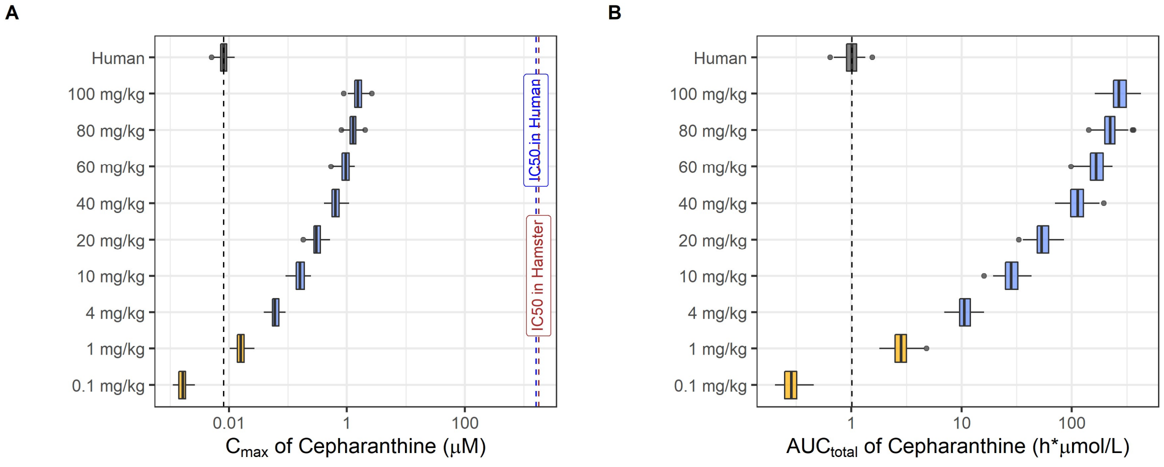

Figure S24. Simulated pharmacokinetic parameters of cepharantine in human and hamster. Predicted pharmacokinetic parameters, i.e., C_{max} (**A**) and AUC_{total} (**B**), in human receiving 60 mg of cepharantine once daily for 10 days (grey box) were compared with pharmacokinetic parameters of hamster receiving 0.1 to 100 mg/kg of cepharantine twice daily for same number of days (coloured boxes, yellow box represents the extrapolation beyond the doses tested in satellite PK studies in hamsters). Boxes and whiskers represent the median with inter-quantile range and the 95% prediction intervals, respectively. Vertical lines denote the IC_{50} (A549-ACE2TMPRSS2 cells), corrected for plasma protein binding in hamster (red line) and human (blue line).

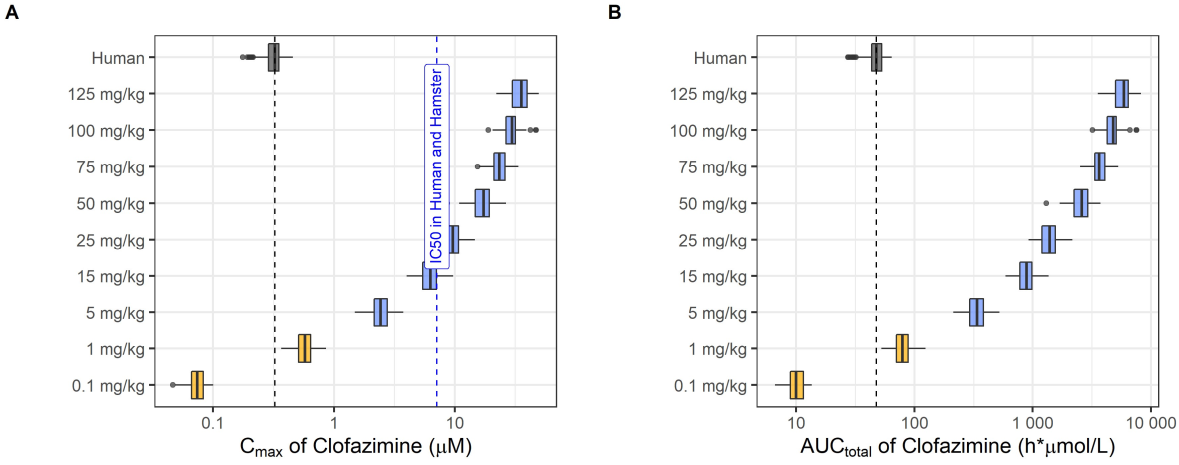

Figure S25. Simulated pharmacokinetic parameters of clofazimine in human and hamster. Predicted pharmacokinetic parameters, i.e., C_{max} (**A**) and AUC_{total} (**B**), in human receiving 100 mg of clofazimine once daily for 10 days (grey box) were compared with pharmacokinetic parameters of hamster receiving 0.1 to 125 mg/kg of clofazimine twice daily for same number of days (coloured boxes, yellow box represents the extrapolation beyond the doses tested in satellite PK studies in hamsters). Boxes and whiskers represent the median with inter-quantile range and the 95% prediction intervals, respectively. Blue vertical line denotes the IC_{50} (A549-ACE2TMPRSS2 cells), corrected for plasma protein binding in hamster and human.

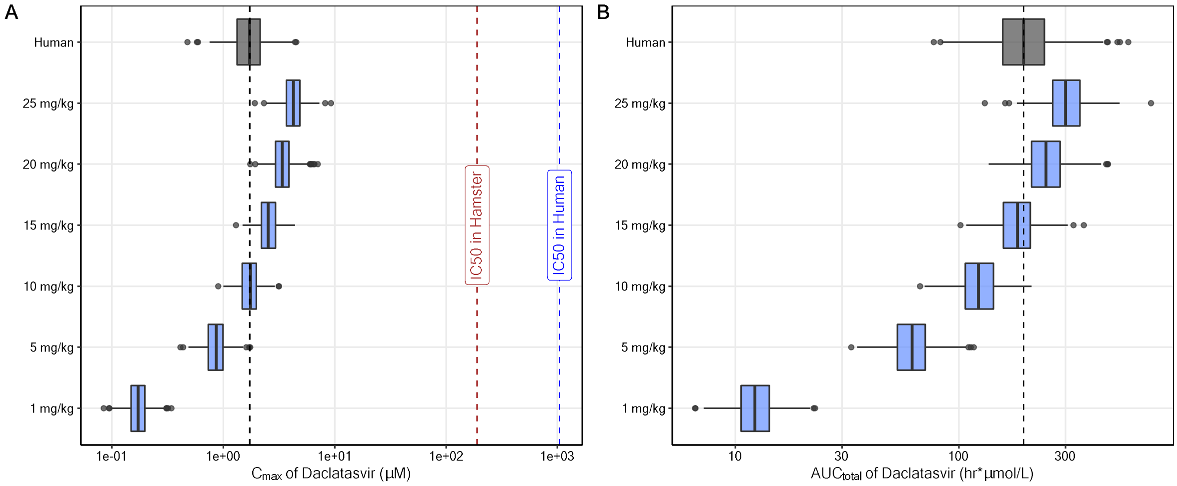

Figure S26. Simulated pharmacokinetic parameters for daclatasvir in human and hamster. Predicted pharmacokinetic parameters, i.e. C_{max} (**A**) and AUC_{total} (**B**), in human associated with receiving 60 mg of daclatasvir once daily for 10 days (grey box) were compared with pharmacokinetic parameters of hamster receiving 1 up to 25 mg/kg daclatasvir twice daily. Boxes and whiskers represent the median with inter-quantile range and the 95% prediction intervals, respectively. Vertical lines denote the IC_{50} (A549-ACE2TMPRSS2 cells), corrected for plasma protein binding in hamster (red line) and human (blue line).

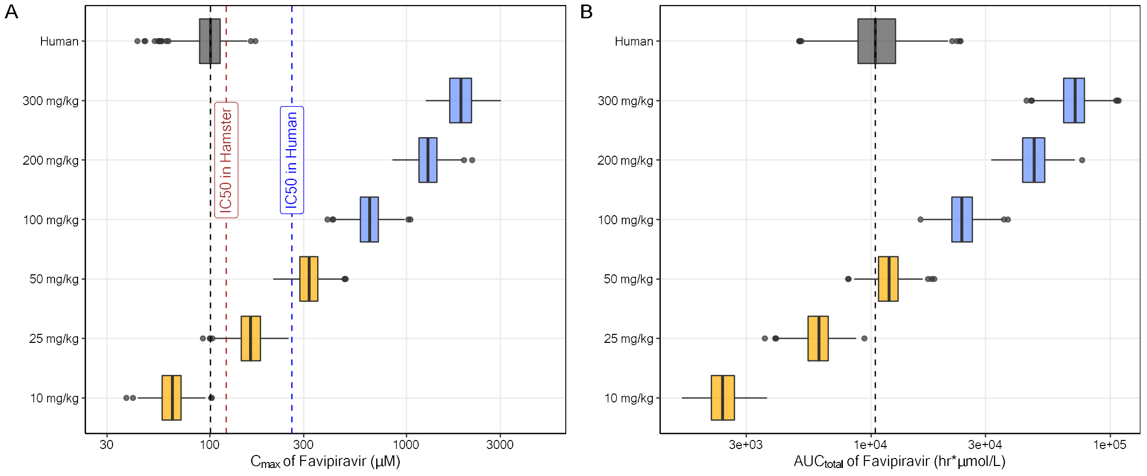

Figure S27. Simulated pharmacokinetic parameters for favipiravir in human and hamster. Predicted pharmacokinetic parameters, i.e. C_{max} (**A**) and AUC_{total} (**B**), in human associated with receiving 600 mg of favipiravir once daily for 10 days (grey box) were compared with pharmacokinetic parameters of hamster receiving 10 up to 300 mg/kg favipiravir twice daily (coloured boxes, yellow box represents the extrapolation beyond the doses tested in satellite PK studies in hamsters). Boxes and whiskers represent the median with inter-quantile range and the 95% prediction intervals, respectively. Vertical lines denote the IC_{50} (A549-ACE2TMPRSS2 cells), corrected for plasma protein binding in hamster (red line) and human (blue line).

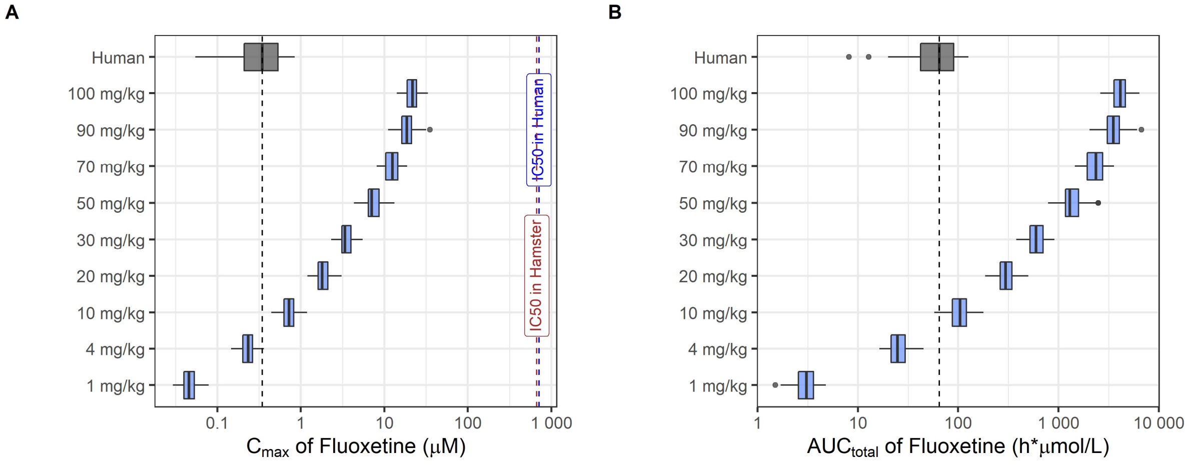

Figure S28. Simulated pharmacokinetic parameters of fluoxetine in human and hamster. Predicted pharmacokinetic parameters, i.e., C_{max} (**A**) and AUC_{total} (**B**), in human receiving 20 mg of fluoxetine once daily for 10 days (grey box) were compared with pharmacokinetic parameters of hamster receiving 1 to 100 mg/kg of fluoxetine twice daily (blue boxes) for same number of days. Boxes and whiskers represent the median with inter-quantile range and the 95% prediction intervals, respectively. Vertical lines denote the IC_{50} (A549-ACE2TMPRSS2 cells), corrected for plasma protein binding in hamster (red line) and human (blue line).

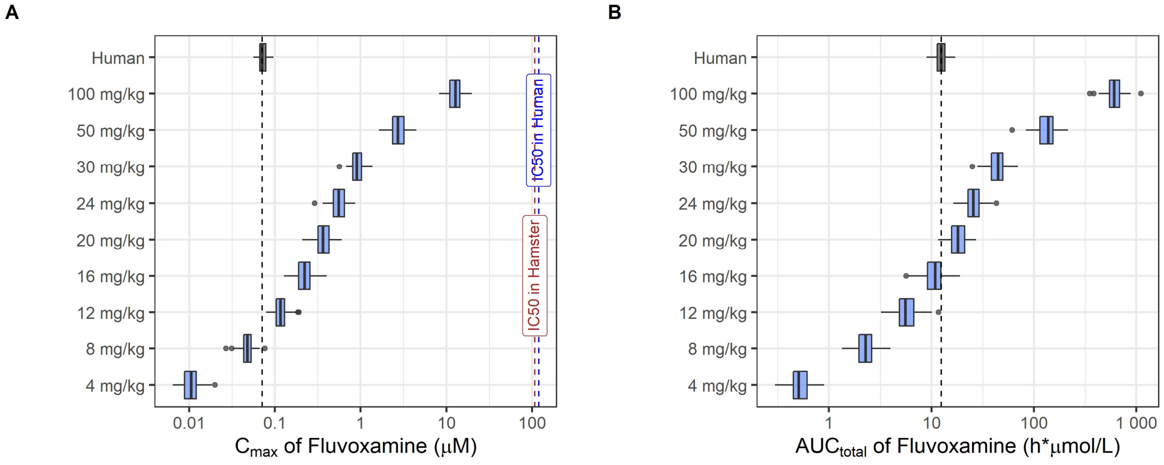

Figure S29. Simulated pharmacokinetic parameters of fluvoxamine in human and hamster. Predicted pharmacokinetic parameters, i.e., C_{max} (**A**) and AUC_{total} (**B**), in human receiving 50 mg of fluvoxamine once daily for 10 days (grey box) were compared with pharmacokinetic parameters of hamster receiving 4 to 100 mg/kg of fluvoxamine twice daily (blue boxes) for same number of days. Boxes and whiskers represent the median with inter-quantile range and the 95% prediction intervals, respectively. Vertical lines denote the IC_{50} (A549-ACE2TMPRSS2 cells), corrected for plasma protein binding in hamster (red line) and human (blue line).

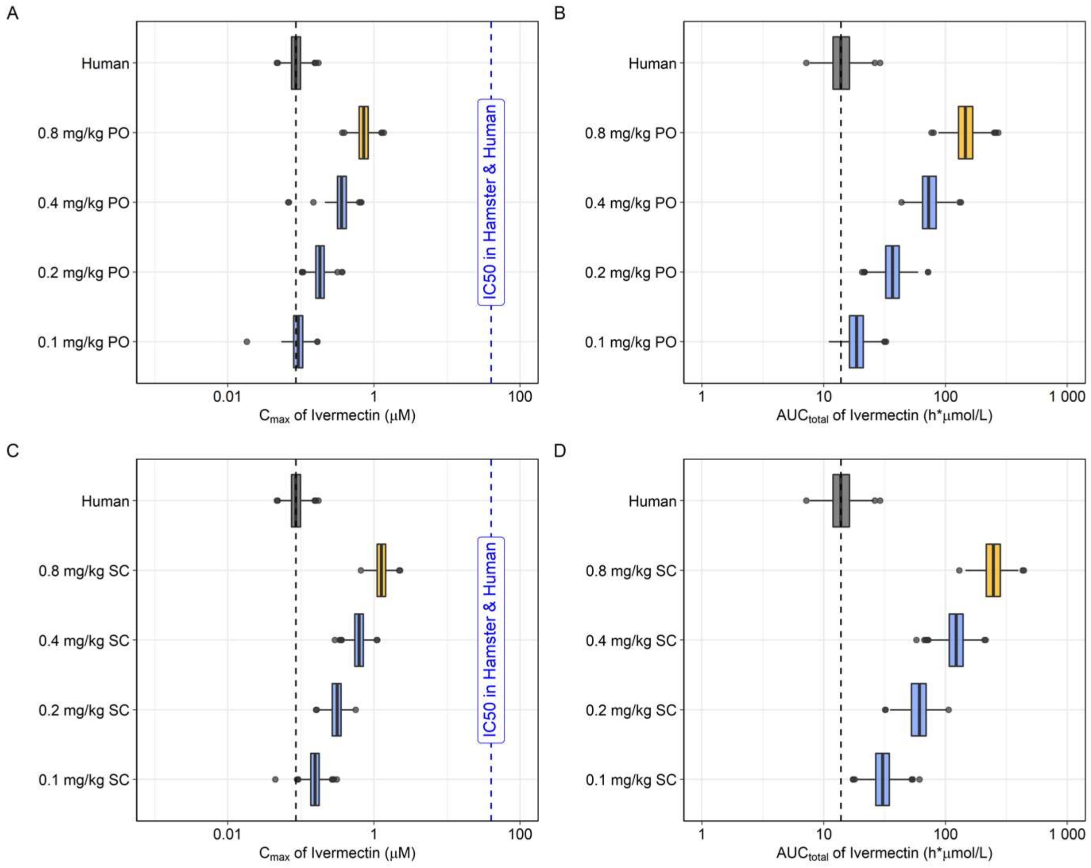

Figure S30. Simulated pharmacokinetic parameters of ivermectin in human and hamster. Predicted pharmacokinetic parameters, i.e. C_{max} (**A**) and AUC_{total} (**B**) following PO administration, C_{max} (**C**) and AUC_{total} (**D**) following subcutaneous administration, in human associated with receiving 0.2 mg/kg of ivermectin once daily for 10 days (grey box) were compared with pharmacokinetic parameters of

hamster receiving 0.1 up to 0.8 mg/kg of ivermectin twice daily (coloured boxes, yellow box represents the extrapolation beyond the study doses in hamsters). Boxes and whiskers represent the median with inter-quantile range and the 95% prediction intervals, respectively. Vertical lines denote the IC_{50} (Vero-hSLAM cells), corrected for plasma protein binding in hamster and human (blue line; hamster plasma protein binding was assumed to be equal to human).

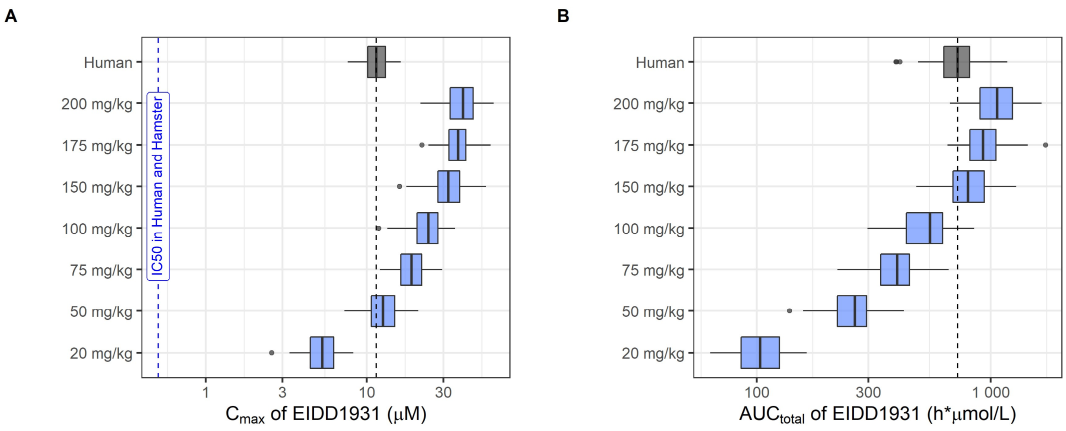

Figure S31. Simulated pharmacokinetic parameters of molnupiravir in human and hamster. Predicted pharmacokinetic parameters, i.e., C_{max} (**A**) and AUC_{total} (**B**), in human receiving 800 mg of molnupiravir twice daily for 10 days (grey box) were compared with pharmacokinetic parameters of hamster receiving 20 to 200 mg/kg of molnupiravir twice daily (blue boxes) for same number of days. Boxes and whiskers represent the median with inter-quantile range and the 95% prediction intervals, respectively. Blue vertical line denotes the IC_{50} (A549-ACE2TMPRSS2 cells), corrected for plasma protein binding in hamster and human.

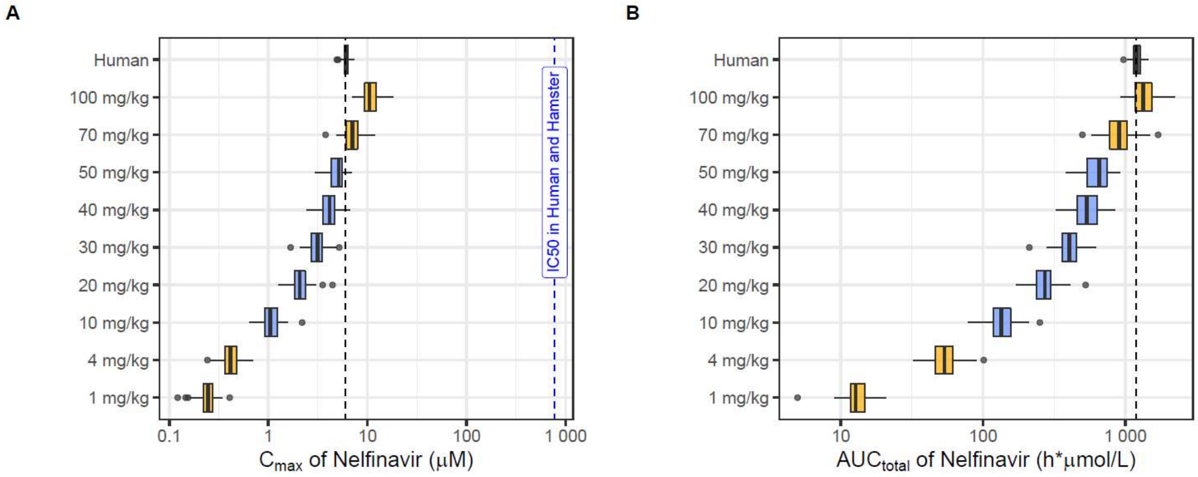

Figure S32. Simulated pharmacokinetic parameters of nelfinavir in human and hamster. Predicted pharmacokinetic parameters, i.e., C_{max} (**A**) and AUC_{total} (**B**), in human receiving 1250 mg of nelfinavir twice daily for 10 days (grey box) were compared with pharmacokinetic parameters of hamster receiving 1 to 100 mg/kg of nelfinavir twice daily for same number of days. Boxes and whiskers represent the median with inter-quantile range and the 95% prediction intervals, respectively. Yellow boxes represent extrapolation beyond the study-doses in hamster. Blue vertical line denotes the IC_{50} (A549-ACE2TMPRSS2 cells), corrected for plasma protein binding in hamster and human.

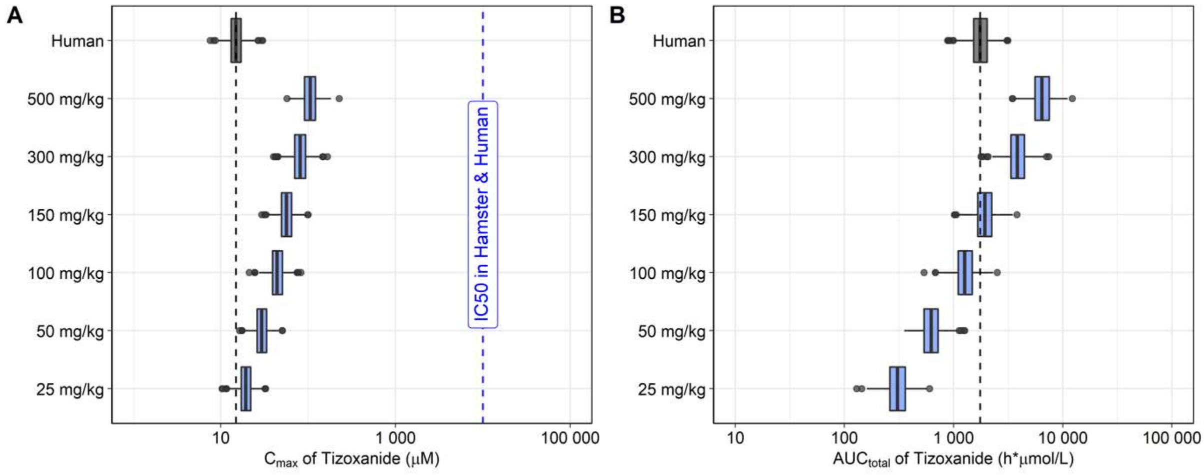

Figure S33. Simulated pharmacokinetic parameters for nitazoxanide in human and hamster. Predicted pharmacokinetic parameters, i.e. C_{max} (**A**) and AUC_{total} (**B**), in human associated with receiving 500 mg of nitazoxanide twice daily for 10 days (grey box) were compared with pharmacokinetic parameters of hamster receiving 25 up to 500 mg/kg nitazoxanide twice daily (coloured boxes, yellow box represents the extrapolation beyond the doses tested in satellite PK studies in hamsters). Boxes and whiskers represent the median with inter-quantile range and the 95% prediction intervals, respectively. Vertical lines denote the IC_{50} (A549-ACE2TMPRSS2 cells), corrected for plasma protein binding in hamster and human (blue line; hamster plasma protein binding was assumed to be equal to human).

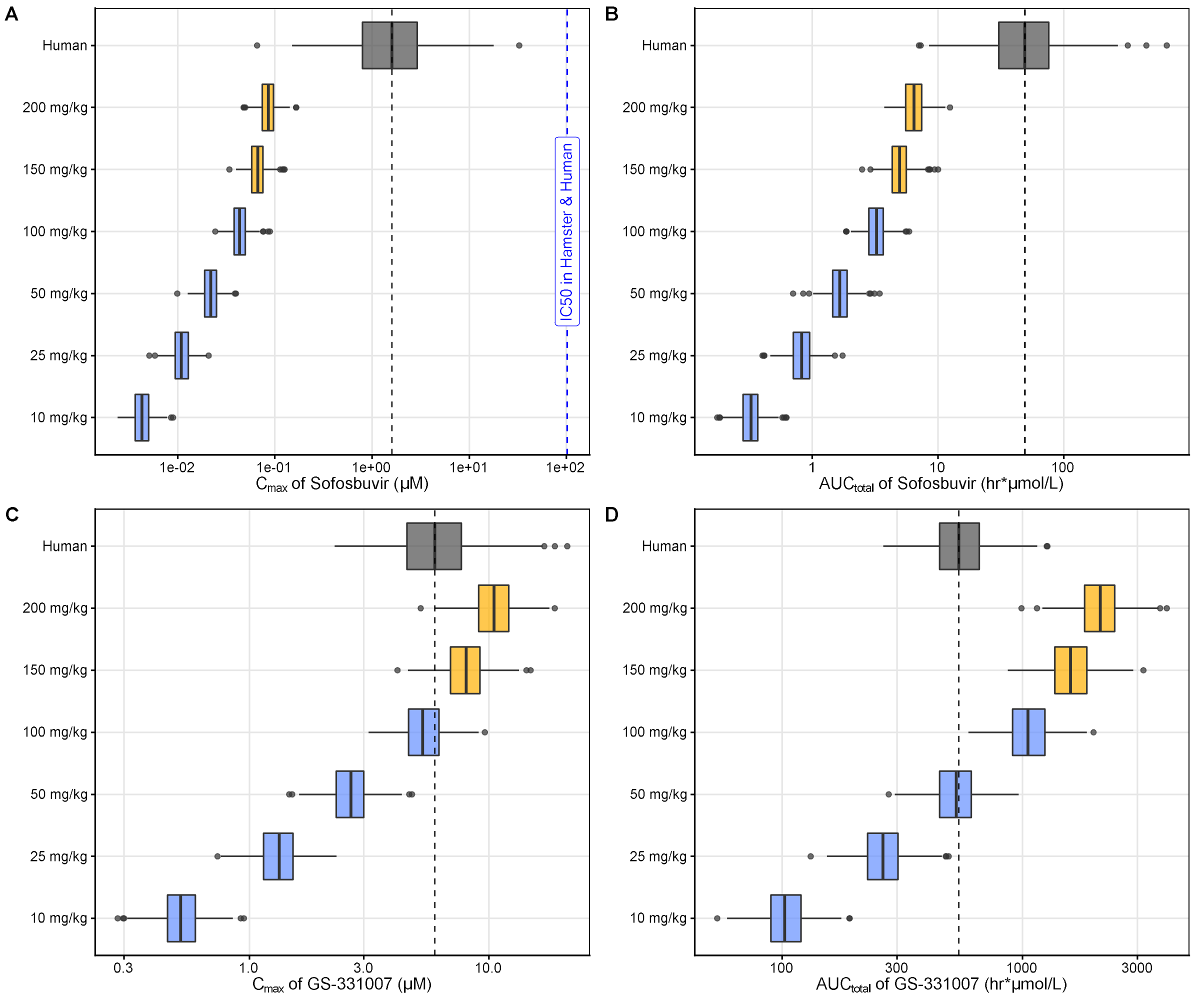

Figure S34. Simulated pharmacokinetic parameters for sofosbuvir and GS-331007 in human and hamster. Predicted pharmacokinetic parameters, i.e. C_{max} of sofosbuvir (**A**) and AUC_{total} of sofosbuvir (**B**), C_{max} of GS-331007 (**C**) and AUC_{total} of GS-331007 (**D**), in human associated with receiving 400 mg of sofosbuvir once daily for 10 days (grey box) were compared with pharmacokinetic parameters of hamster receiving 10 to 200 mg/kg sofosbuvir twice daily (coloured boxes, yellow box represents the extrapolation beyond the doses tested in satellite PK studies in hamsters). Boxes and whiskers represent the median with inter-quantile range and the 95% prediction intervals, respectively. Vertical lines denote the IC_{50} (A549-ACE2TMPRSS2 cells), corrected for plasma protein binding in hamster (red line) and human (blue line).

Table S28. Exposure in human at clinical dose and doses in hamster matching human pharmacokinetic parameters.

Dosing regimen			C_{max}		AUC_{total}[c]	
**Compound**	**Clinical dose / dosing frequency**[a]	**Doses simulated in hamster (mg/kg)**[b]	**Median C_{max} in human at clinical dose [uM]**	**Dose range in hamster reaching C_{max} in human (mg/kg)**[d]	**Median AUC_{total} in human at clinical dose (h*µmol/L)**	**Dose range in hamster reaching AUC_{total} in human (mg/kg)**[d]
Ambroxol	30 mg / twice daily	3; 15; 30; 50; 75; 90; 100	0.228	~30	34.5	30 – 50
Amodiaquine	540 mg / once daily	5; 10; 20; 30; 50; 100; 200	0.257	< 5	16.5	< 5
AT-273	NA	10; 50; 100; 250; 500; 750; 1000	NA	NA	NA	NA
Atazanavir / ritonavir-boosted	300 mg / once daily	12; 24; 48; 72; 96; 120	3.55	~24	620	48 - 72
Cepharantine	60 mg / once daily [85]	0.1; 1; 4; 10; 20; 40; 60; 80; 100	0.00816	0.1 – 1	1.015	0.1 – 1
Clofazimine	100 mg / once daily	0.1; 1; 5; 15; 25; 50; 75; 100; 125	0.322	0.1 – 1	48.6	0.1 – 1
Daclatasvir	60 mg / once daily	1; 5; 10; 15; 20; 25	1.14	~10	132	~15
Favipiravir	600 mg / once daily	10; 25; 50; 100; 200; 300	97.7	10 - 25	10000	25 - 50
Fluoxetine	20 mg / once daily	1; 4; 10; 20; 30; 50; 70; 90; 100	0.333	4 – 10	62.6	4 – 10
Fluvoxamine	50 mg / once daily	4; 8; 12; 16; 20; 24; 30; 50; 100	0.0712	8 – 12	12.020	16 – 20
Ivermectin	0.2 mg/kg / once daily	0.1; 0.2; 0.4; 0.8	0.0853	~0.1	13.9	~0.1
Molnupiravir	800 mg / twice daily	20; 50; 75; 100; 150; 175; 200	12.074	~50	743	~150
Nelfinavir	1250 mg / twice daily	1; 4; 10; 20; 30; 40; 50; 70; 100	6.210	50 – 70	1215	~100
Nitazoxanide	500 mg / twice daily	25; 50; 100; 150; 300; 500	14.9	~25	1751	~150
Sofosbuvir	400 mg / once daily	10; 25; 50; 100; 150; 200	1.67	> 200	51.9	> 200

[a]Simulated pharmacokinetic profiles in human were based on standard daily dosing (oral) according to the product information unless otherwise stated. Simulations were performed for a total treatment duration of 10 days.
[b]Simulated pharmacokinetic profiles in hamster were based on twice daily dosing and a total treatment duration of 10 days.
[c]AUC_{total} refers to cumulative AUC from time 0 to12 h after the last dose (AUC_{0-252h}).
[d]If the median of C_{max} or AUC_{total} in human is falling into the interquartile range of C_{max} or AUC_{total} in hamster at a particular dose, then this dose is selected. Otherwise a dose range is given.

S2.8. In vivo efficacy in hamster

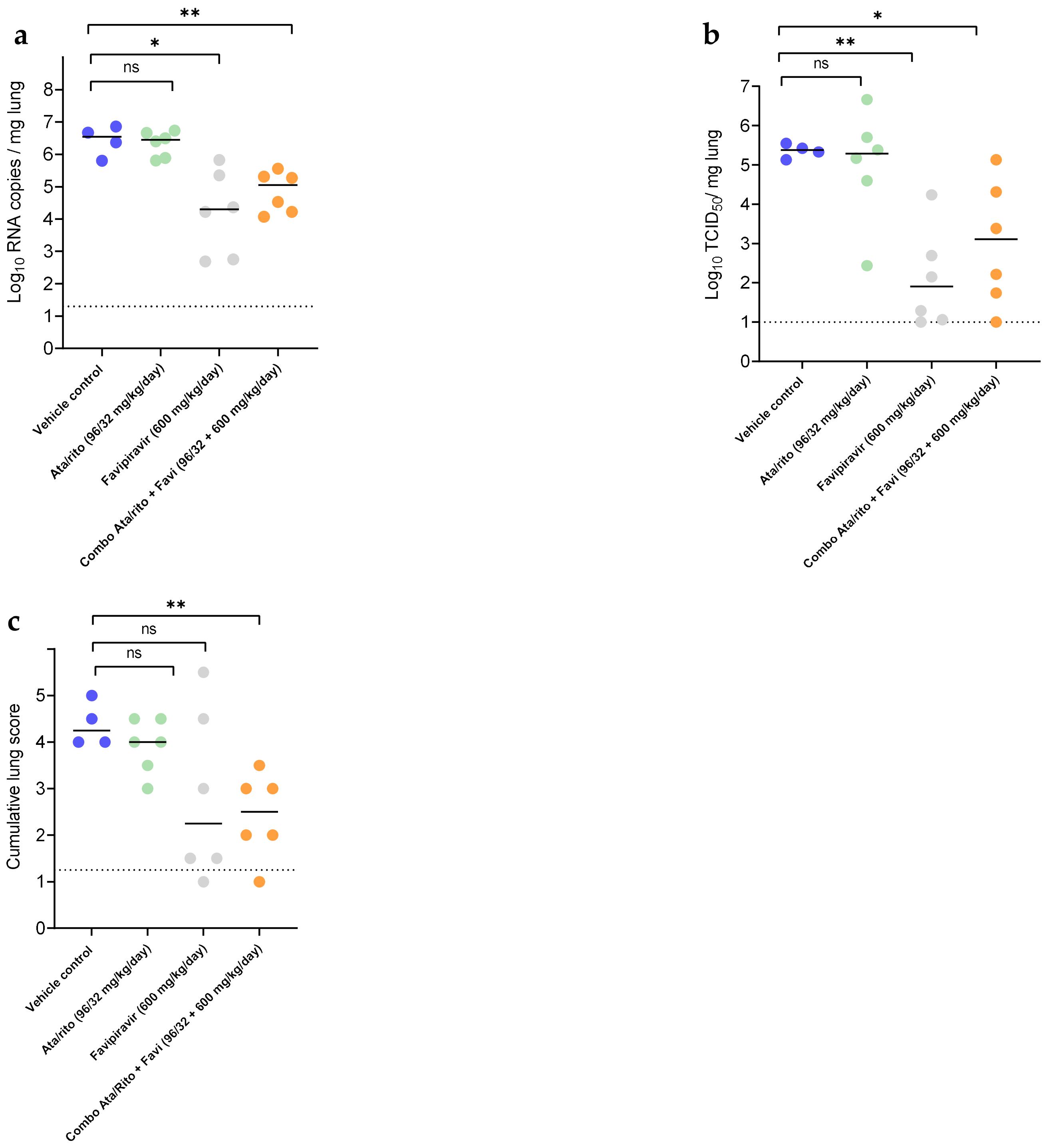

Figure S35. *In vivo* efficacy of ritonavir-boosted atazanavir, favipiravir and combination thereof in SARS-CoV-2 hamster model.

In vivo efficacy of ritonavir-boosted atazanavir, favipiravir and combination thereof against SARS-CoV-2 (Wuhan) wild type strain in Syrian hamsters. **a**) Viral RNA levels in the lungs of control (vehicle-treated) and ritonavir-boosted atazanavir, favipiravir and combination -treated (at 48/16 or 300 mg/kg, BID) SARS-CoV-2–infected hamsters at day 4 post-infection (pi). Individual data and median values (indicated by bars) are presented and are expressed as log_{10} SARS-CoV-2 RNA copies per mg lung tissue. Data were

analyzed with the Mann–Whitney U test (two-sided). ns: not significant, *P = 0.0190, **P = 0.0095. **b**) Infectious viral loads in the lungs of control (vehicle-treated), ritonavir-boosted atazanavir, favipiravir and combination -treated SARS-CoV-2-infected hamsters at day 4 pi (expressed as $\log_{10}$ $TCID_{50}$ per mg lung tissue). Individual data and median values (indicated by bars) are presented. Data were analyzed with the Mann–Whitney U test (two-sided). ns: not significant, **P = 0.0095, *P = 0.0143. **c**) Cumulative severity score from H&E staining slides of lungs from control (vehicle-treated) and ritonavir-boosted atazanavir, favipiravir and combination -treated hamsters. Individual data and median values (indicated by bars) are presented and the dotted line represents the median score of untreated non-infected hamsters. Data were analysed with the Mann–Whitney U test (two-sided). ns: not significant , **P = 0.0048.

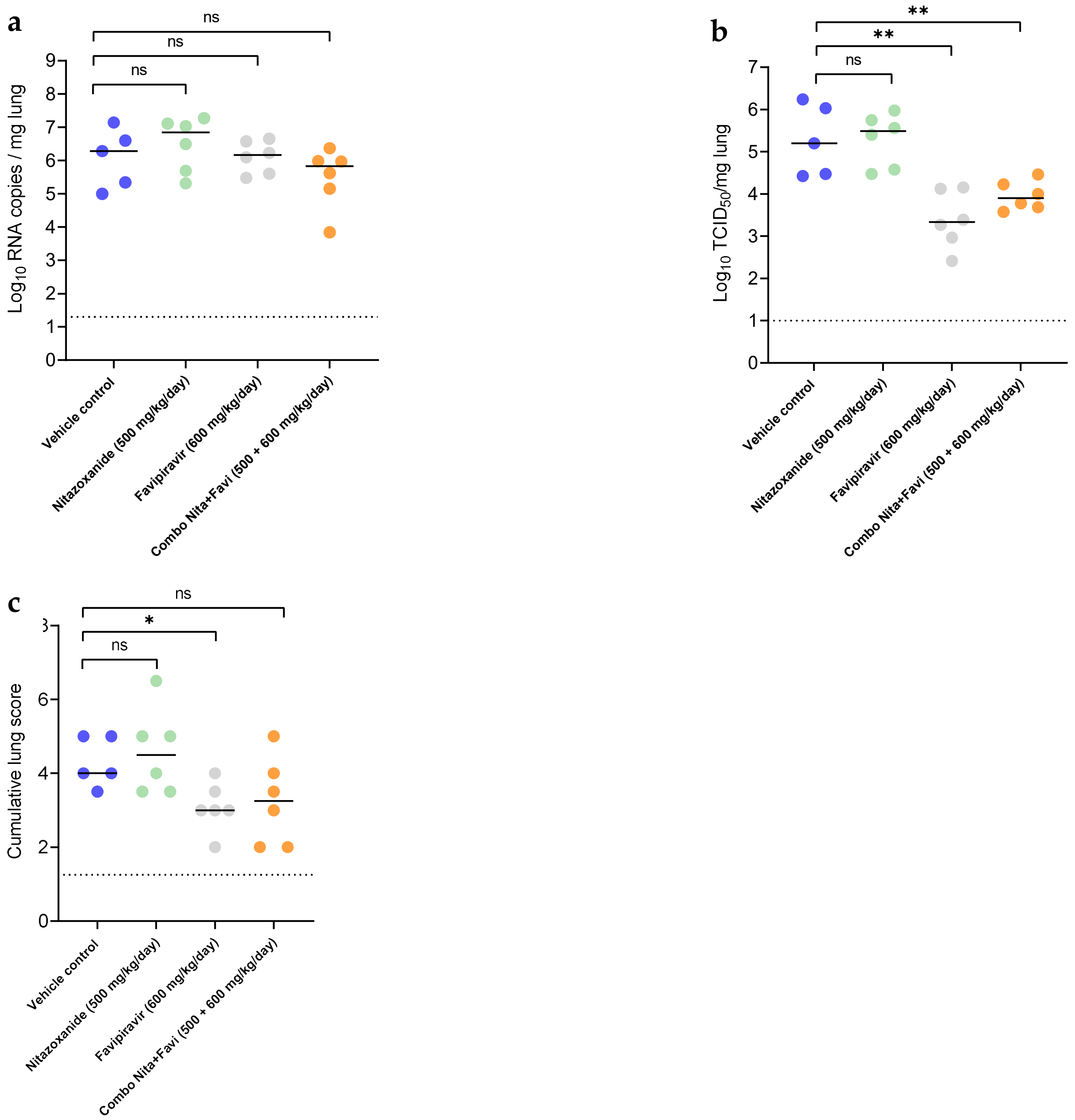

Figure S36. *In vivo* efficacy of nitazoxanide, favipiravir and combination thereof in SARS-CoV-2 hamster model.

In vivo efficacy of nitazoxanide, favipiravir and combination thereof against SARS-CoV-2 (Wuhan) wild type strain in Syrian hamsters. **a**) Viral RNA levels in the lungs of control (vehicle-treated) and nitazoxanide, favipiravir and combination thereof -treated (at 250 and 300 mg/kg, BID) SARS-CoV-2–infected hamsters at day 4 post-infection (pi). Individual data and median values (indicated by bars) are presented and are expressed as log_{10} SARS-CoV-2 RNA copies per mg lung tissue. Data were analysed with the Mann–Whitney U test (two-sided). ns: not significant. **b**) Infectious viral loads in the lungs of control (vehicle-treated), nitazoxanide, favipiravir and combination thereof -treated SARS-CoV-2-infected hamsters at day 4 pi (expressed as log_{10} $TCID_{50}$ per mg lung tissue). Individual data and median values (indicated by bars) are presented. Data were analyzed with the Mann–Whitney U test (two-sided). ns: not significant, **P = 0.0043, 0.0087. **c**) Cumulative severity score from H&E staining slides of lungs from control (vehicle-treated) and nitazoxanide, favipiravir and combination thereof -treated hamsters. Individual data and median values (indicated by bars) are presented and the dotted line represents the median score of untreated non-infected hamsters. Data were analysed with the Mann–Whitney U test (two-sided). ns: not significant, *P = 0.0195.

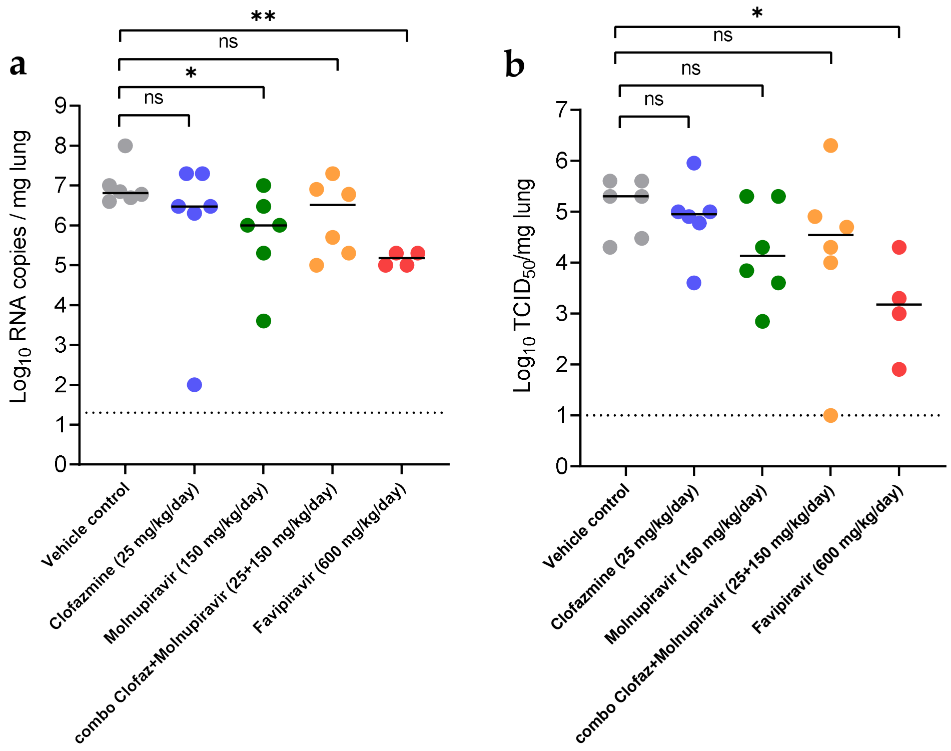

Figure S37. *In vivo* efficacy of clofazimine, molnupiravir and combination thereof in SARS-CoV-2 hamster model.

In vivo efficacy of clofazimine, molnupiravir and combination thereof against SARS-CoV-2 (Wuhan) wild type strain in Syrian hamsters. **a**) Viral RNA levels in the lungs of control (vehicle-treated) and clofazimine, molnupiravir and combination thereof - treated (at 25 mg/kg QD and 75 mg/kg, BID) SARS-CoV-2–infected hamsters at day 4 post-infection (pi). Individual data and median values (indicated by bars) are presented and are expressed as log_{10} SARS-CoV-2 RNA copies per mg lung tissue. Data were analysed with the Mann–Whitney U test (two-sided). ns: not significant, *P = 0.0303, **P = 0.0095. **b**) Infectious viral loads in the lungs of control (vehicle-treated), clofazimine, molnupiravir and

combination thereof -treated SARS-CoV-2-infected hamsters at day 4 pi (expressed as log_{10} $TCID_{50}$ per mg lung tissue). Individual data and median values (indicated by bars) are presented. Data were analysed with the Mann–Whitney U test (two-sided). ns: not significant, *P = 0.0143.

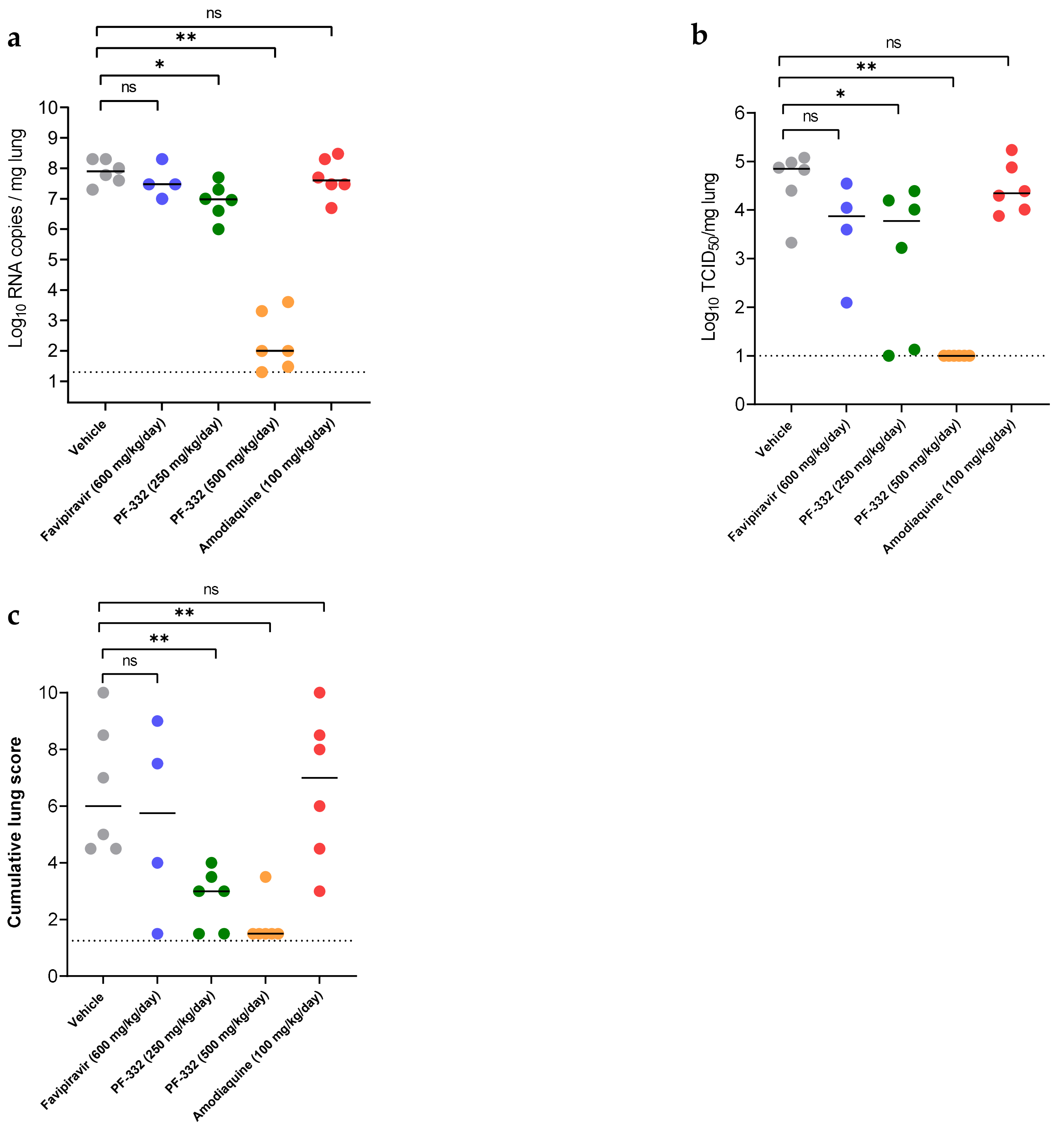

Figure S38. *In vivo* efficacy of nirmatrelvir (PF-332) and amodiaquine in SARS-CoV-2 hamster model.

In vivo efficacy of nirmatrelvir (PF-332) and amodiaquine against Beta SARS-CoV-2 (B.1.351) variant in Syrian hamsters. **a**) Viral RNA levels in the lungs of control (vehicle-treated) and nirmatrelvir (PF-332) and amodiaquine - treated (at 125 or 250 mg/kg, BID

and 100 mg/kg QD) SARS-CoV-2−infected hamsters at day 4 post-infection (pi). Individual data and median values (indicated by bars) are presented and are expressed as log_{10} SARS-CoV-2 RNA copies per mg lung tissue. Data were analysed with the Mann–Whitney U test (two-sided). ns: not significant,*P = 0.0108, **P = 0.0022. **b**) Infectious viral loads in the lungs of control (vehicle-treated), nirmatrelvir (PF-332) and amodiaquine -treated SARS-CoV-2-infected hamsters at day 4 pi (expressed as log_{10} $TCID_{50}$ per mg lung tissue). Individual data and median values (indicated by bars) are presented. Data were analysed with the Mann–Whitney U test (two-sided). ns: not significant, *P = 0.0152, **P = 0.0022. **c**) Cumulative severity score from H&E staining slides of lungs from control (vehicle-treated), nirmatrelvir (PF-332) and amodiaquine -treated hamsters. Individual data and median values (indicated by bars) are presented, and the dotted line represents the median score of untreated non-infected hamsters. Data were analysed with the Mann–Whitney U test (two-sided). ns: not significant, **P = 0.0022.

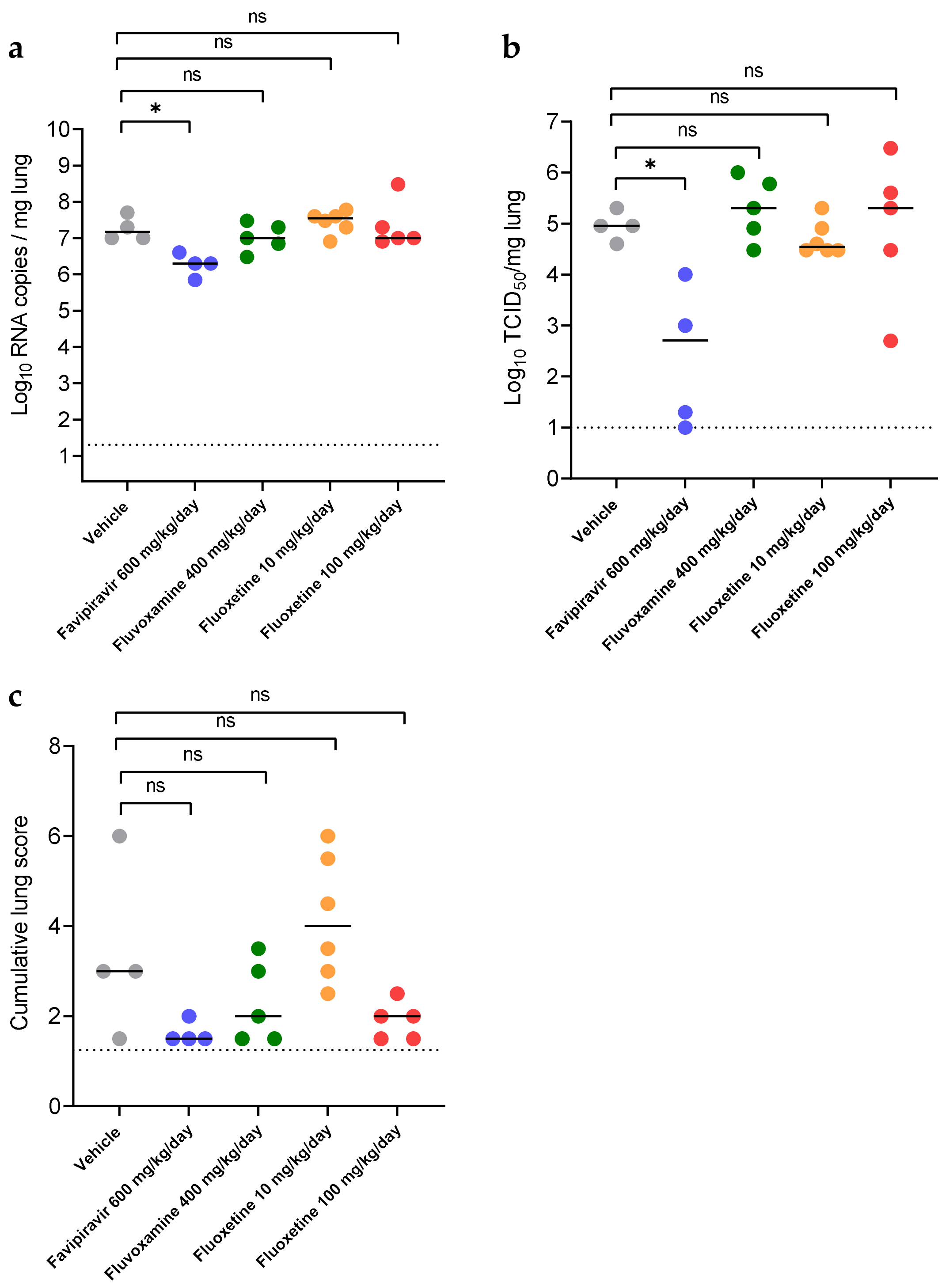

Figure S39. *In vivo* efficacy of fluvoxamine and fluoxetine in SARS-CoV-2 hamster model.

In vivo efficacy of fluvoxamine and fluoxetine against Beta SARS-CoV-2 (B.1.351) variant in Syrian hamsters. **a**) Viral RNA levels in the lungs of control (vehicle-treated), fluvoxamine and fluoxetine - treated (at 200 mg/kg BID and 10 or 100 mg/kg, QD) SARS-CoV-2–infected hamsters at day 4 post-infection (pi). Individual data and median values (indicated by bars) are presented and are expressed as log_{10} SARS-CoV-2 RNA copies per mg lung tissue. Data were analysed with the Mann–Whitney U test (two-sided). ns: not significant, *P = 0.0286. **b**) Infectious viral loads in the lungs of control (vehicle-treated),

fluvoxamine and fluoxetine -treated SARS-CoV-2-infected hamsters at day 4 pi (expressed as $\log_{10}$ $TCID_{50}$ per mg lung tissue). Individual data and median values (indicated by bars) are presented. Data were analysed with the Mann–Whitney U test (two-sided). ns: not significant, *P = 0.0286. **c**) Cumulative severity score from H&E staining slides of lungs from control (vehicle-treated), fluvoxamine and fluoxetine -treated hamsters. Individual data and median values (indicated by bars) are presented, and the dotted line represents the median score of untreated non-infected hamsters. Data were analysed with the Mann–Whitney U test (two-sided). ns: not significant.

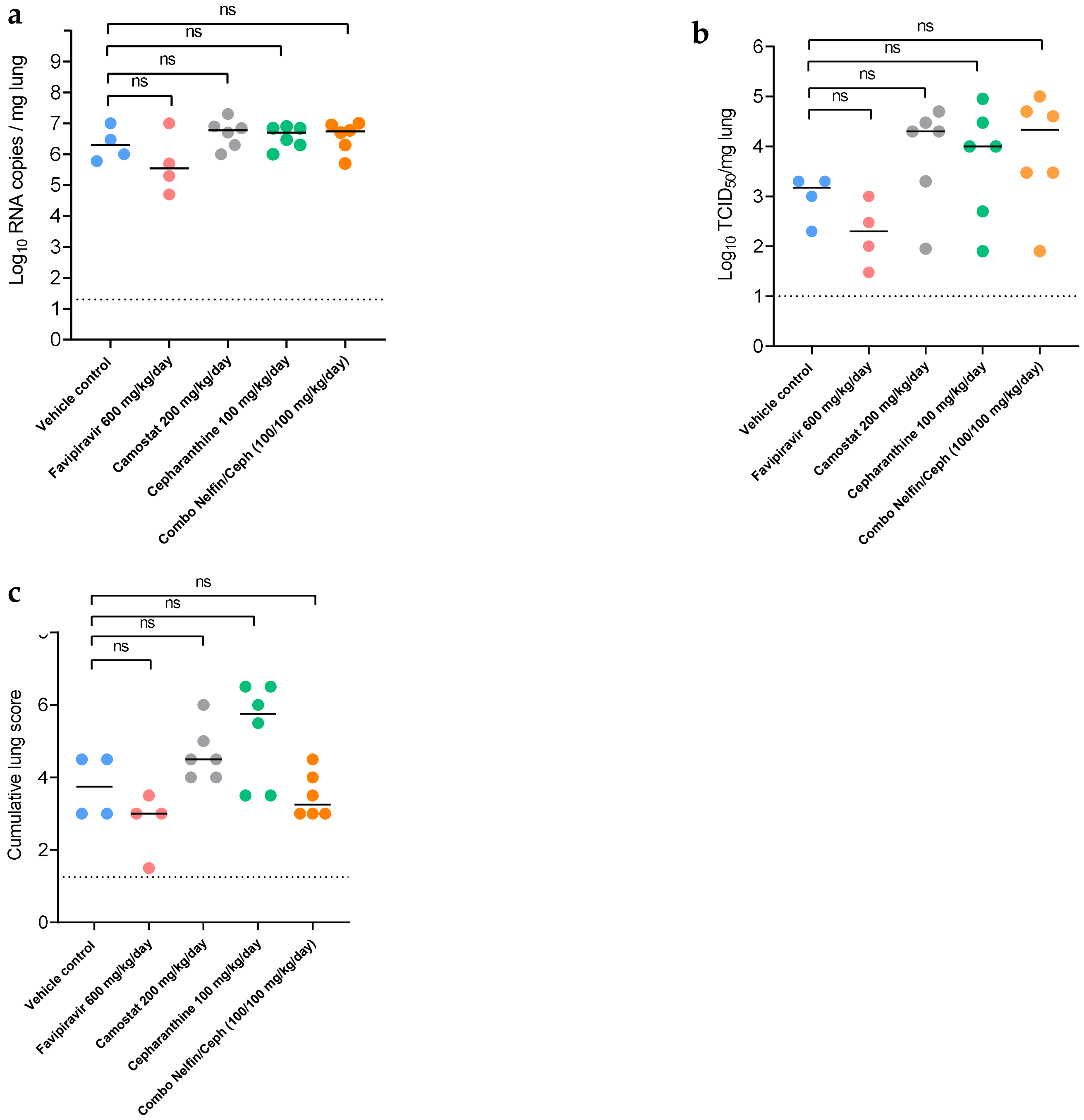

Figure S40. *In vivo* efficacy of camostat, cepharanthine and combination of nelfinavir/cepharanthine in SARS-CoV-2 hamster model.

In vivo efficacy of camostat, cepharanthine and combination of nelfinavir/cepharanthine against Beta SARS-CoV-2 (B.1.351) variant in Syrian hamsters. **a**) Viral RNA levels in the lungs of control (vehicle-treated), camostat, cepharanthine and combination of nelfinavir/cepharanthine - treated (at 100 mg/kg BID, 100 mg/kg QD and 50/100 mg/kg BID/QD) SARS-CoV-2–infected hamsters at day 4 post-infection (pi). Individual data and median values (indicated by bars) are presented and are expressed as $\log_{10}$ SARS-CoV-2 RNA copies per mg lung tissue. Data were analysed with the Mann–Whitney U test (two-sided). ns: not significant. **b**) Infectious viral loads in the lungs of control (vehicle-treated), camostat, cepharanthine and combination of nelfinavir/cepharanthine -treated SARS-CoV-2-infected hamsters at day 4 pi (expressed as $\log_{10}$ $TCID_{50}$ per mg lung tissue). Individual data and median values (indicated by bars) are presented. Data were analysed with the Mann–Whitney U test (two-sided). ns: not significant. **c**) Cumulative severity score from H&E staining slides of lungs from control (vehicle-treated), camostat, cepharanthine and combination of nelfinavir/cepharanthine - treated hamsters. Individual data and median values (indicated by bars) are presented, and the dotted line represents the median score of untreated non-infected hamsters. Data were analysed with the Mann–Whitney U test (two-sided). ns: not significant.

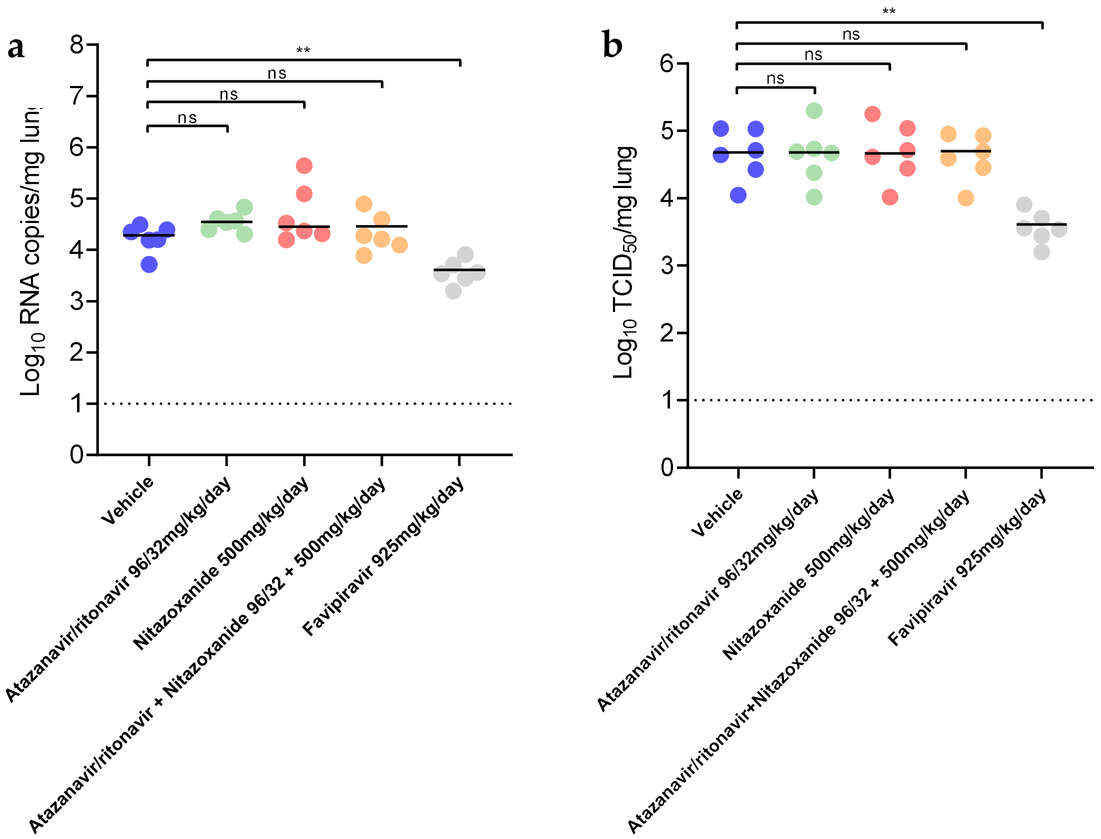

Figure S41. *In vivo* efficacy of ritonavir-boosted atazanavir, nitazoxanide and combination thereof in SARS-CoV-2 hamster model.

In vivo efficacy of ritonavir-boosted atazanavir, nitazoxanide and combination thereof against SARS-CoV-2 (BavPat1) wild type strain in Syrian hamsters. **a**) Viral RNA levels in the lungs of control (vehicle-treated) and ritonavir-boosted atazanavir, nitazoxanide and combination thereof -treated (at 48/16 or 250 mg/kg, BID) SARS-CoV-2–infected hamsters at day 3 post-infection (pi). Individual data and median values (indicated by bars) are presented and are expressed as $\log_{10}$ SARS-CoV-2 RNA copies per mg lung tissue. Data were analyzed with the Mann–Whitney U test (two-sided). Favipiravir is used

as positive control (at 925 mg/kg/day). ns: not significant, **P = 0.0043. **b**) Infectious viral loads in the lungs of control (vehicle-treated), ritonavir-boosted atazanavir, nitazoxanide and combination thereof -treated SARS-CoV-2-infected hamsters at day 3 pi (expressed as $\log_{10}$ TCID$_{50}$ per mg lung tissue). Individual data and median values (indicated by bars) are presented. Data were analyzed with the Mann–Whitney U test (two-sided). Favipiravir is used as positive control (at 925 mg/kg/day). ns: not significant, **P = 0.0022.

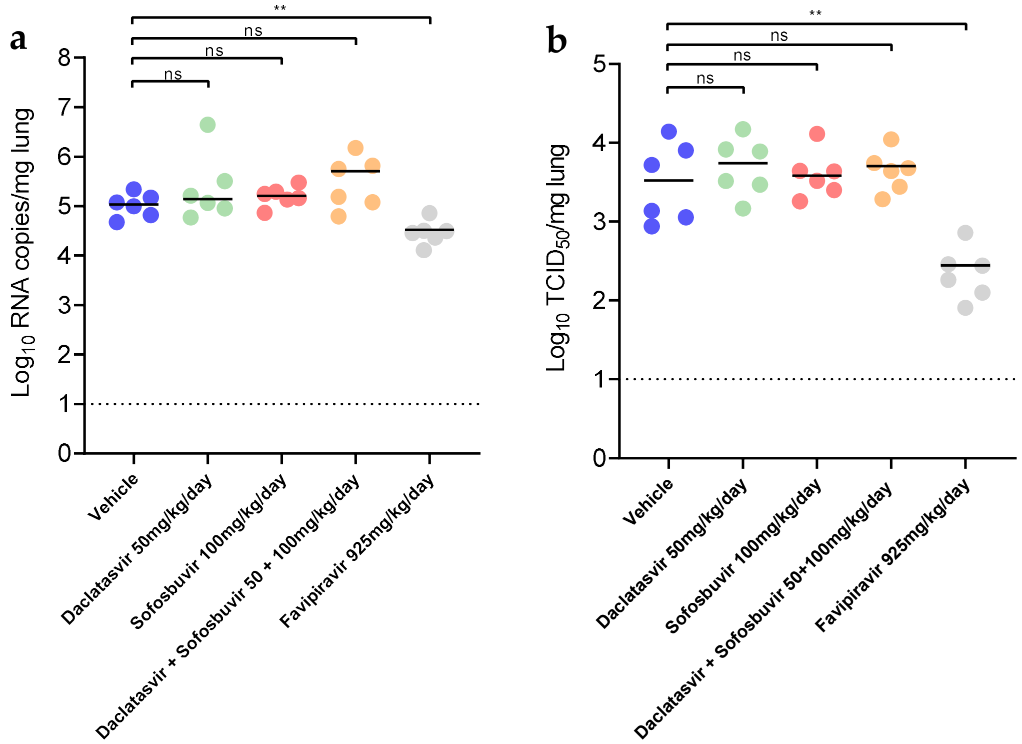

Figure S42. *In vivo* efficacy of sofosbuvir and daclatasvir and combination thereof in SARS-CoV-2 hamster model.

In vivo efficacy of sofosbuvir and daclatasvir and combination thereof against SARS-CoV-2 (BavPat1) wild type strain in Syrian hamsters. **a**) Viral RNA levels in the lungs of control (vehicle-treated), sofosbuvir and daclatasvir and combination thereof -treated (at 100 mg/kg QD or 25 mg/kg, BID) SARS-CoV-2–infected hamsters at day 3 post-infection (pi). Individual data and median values (indicated by bars) are presented and are expressed as $\log_{10}$ SARS-CoV-2 RNA copies per mg lung tissue. Data were analyzed with the Mann–Whitney U test (two-sided). Favipiravir is used as positive control (at 925 mg/kg/day). ns: not significant, **P = 0.0087. **b**) Infectious viral loads in the lungs of control (vehicle-treated), sofosbuvir and daclatasvir and combination thereof -treated SARS-CoV-2-infected hamsters at day 3 pi (expressed as $\log_{10}$ TCID$_{50}$ per mg lung tissue). Individual data and median values (indicated by bars) are presented. Data were analyzed with the Mann–Whitney U test (two-sided). Favipiravir is used as positive control (at 925 mg/kg/day). ns: not significant, **P = 0.0022.

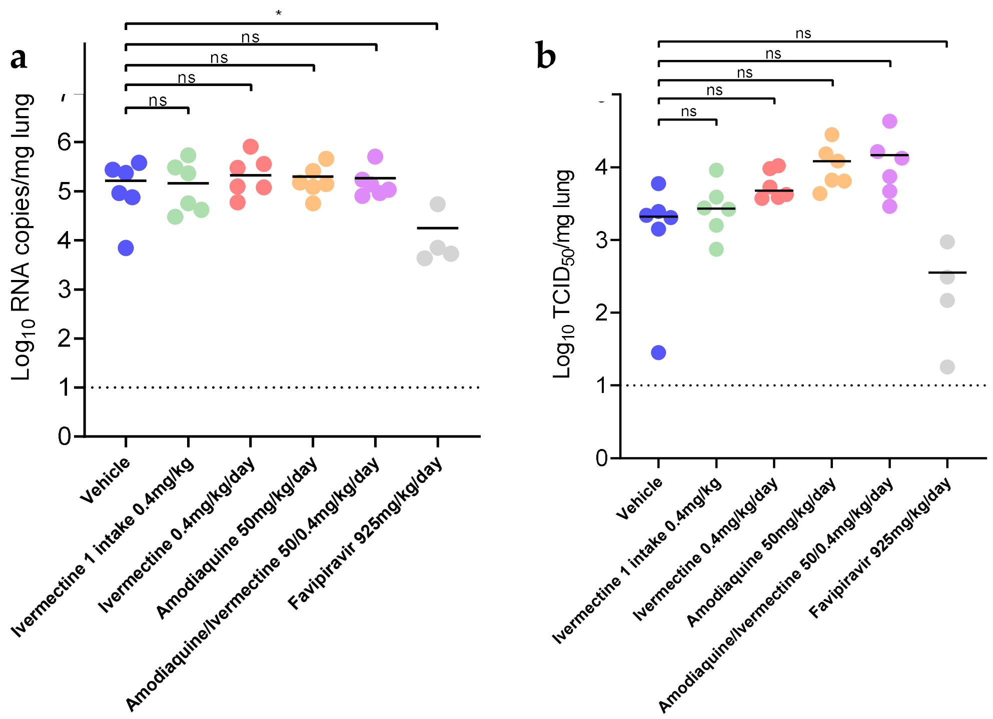

Figure S43. *In vivo* efficacy of ivermectin and amodiaquine and combination thereof in SARS-CoV-2 hamster model.

In vivo efficacy of ivermectin and amodiaquine and combination thereof against SARS-CoV-2 (BavPat1) wild type strain in Syrian hamsters. **a**) Viral RNA levels in the lungs of control (vehicle-treated), ivermectin and amodiaquine and combination thereof -treated (at 0.4 mg/kg 1 intake or QD and 50 mg/kg, QD) SARS-CoV-2–infected hamsters at day 4 post-infection (pi). Individual data and median values (indicated by bars) are presented and are expressed as log_{10} SARS-CoV-2 RNA copies per mg lung tissue. Data were analyzed with the Mann–Whitney U test (two-sided). Favipiravir is used as positive control (at 925 mg/kg/day). ns: not significant, *P = 0.0381. **b**) Infectious viral loads in the lungs of control (vehicle-treated), ivermectin and amodiaquine and combination thereof -treated SARS-CoV-2-infected hamsters at day 4 pi (expressed as log_{10} $TCID_{50}$ per mg lung tissue). Individual data and median values (indicated by bars) are presented. Data were analyzed with the Mann–Whitney U test (two-sided). Favipiravir is used as positive control (at 925 mg/kg/day). Amodiaquine treated groups (monotherapy and combination) started 1-day prior infection. ns: not significant.

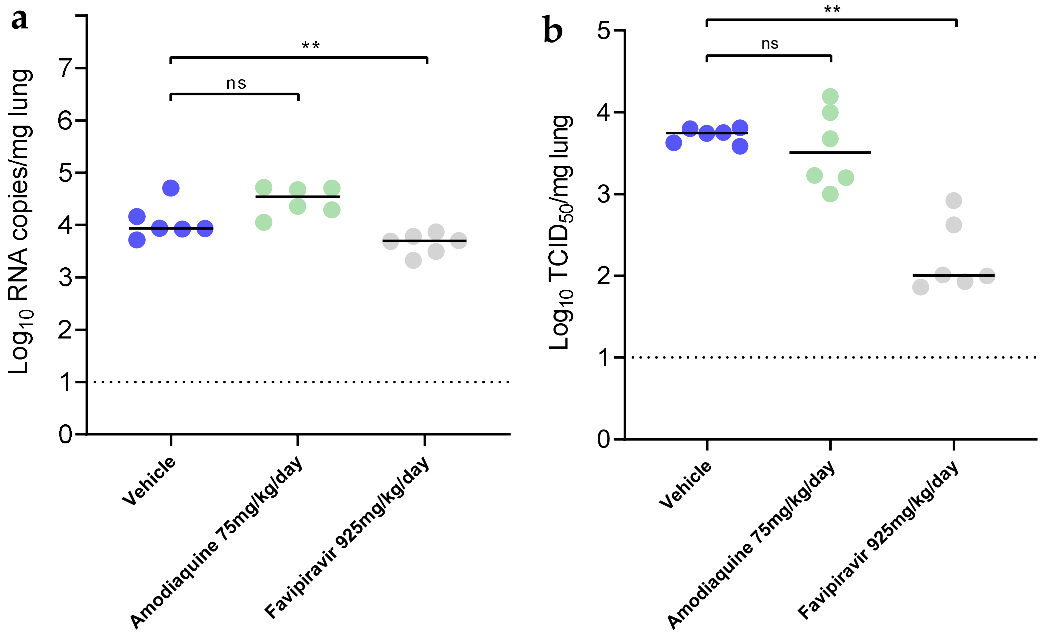

Figure S44. *In vivo* efficacy of amodiaquine in SARS-CoV-2 hamster model (repeat).

In vivo efficacy of amodiaquine against SARS-CoV-2 (BavPat1) wild type strain in Syrian hamsters. **a**) Viral RNA levels in the lungs of control (vehicle-treated), amodiaquine -treated (at 75 mg/kg, QD) SARS-CoV-2–infected hamsters at day 4 post-infection (pi). Individual data and median values (indicated by bars) are presented and are expressed as log_{10} SARS-CoV-2 RNA copies per mg lung tissue. Data were analyzed with the Mann–Whitney U test (two-sided). Favipiravir is used as positive control (at 925 mg/kg/day). ns: not significant, **P = 0.0087. **b**) Infectious viral loads in the lungs of control (vehicle-treated), amodiaquine-treated SARS-CoV-2-infected hamsters at day 4 pi (expressed as log_{10} $TCID_{50}$ per mg lung tissue). Individual data and median values (indicated by bars) are presented. Data were analyzed with the Mann–Whitney U test (two-sided). Favipiravir is used as positive control (at 925 mg/kg/day). ns: not significant, **P = 0.0022.

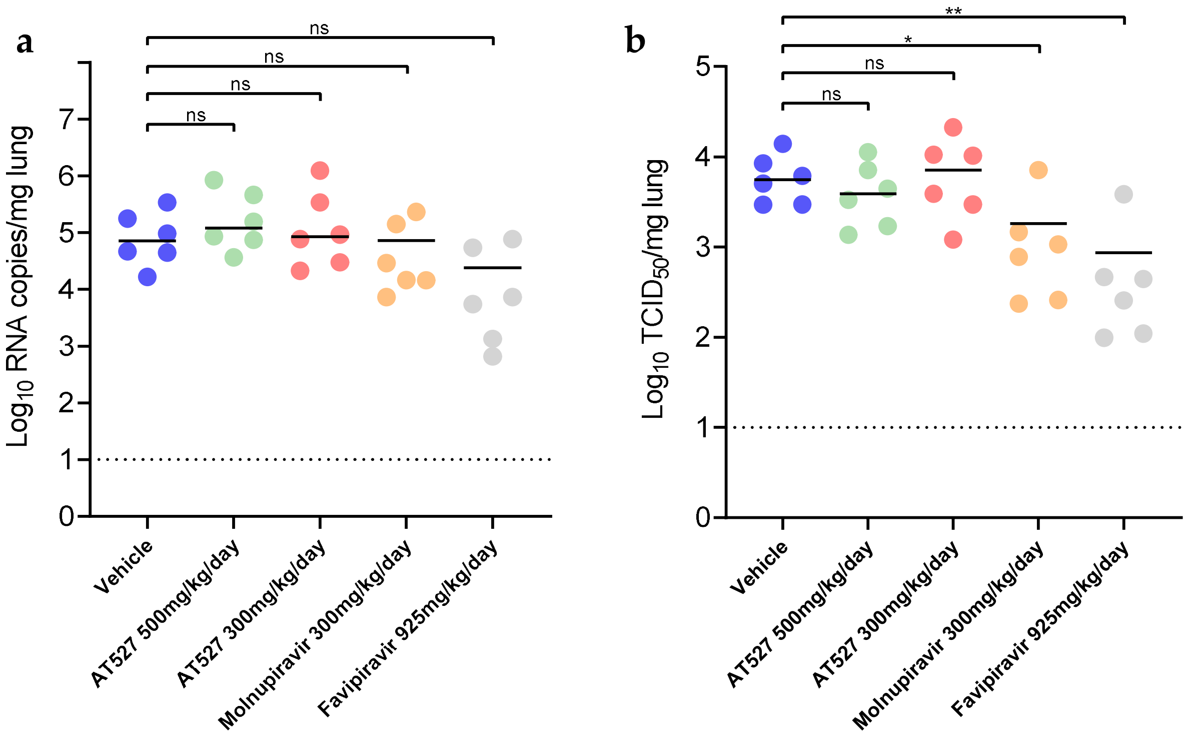

Figure S45. *In vivo* efficacy of bemnifosbuvir (AT-527) and molnupiravir in SARS-CoV-2 hamster model.

In vivo efficacy of AT-527 against SARS-CoV-2 (BavPat1) wild type strain in Syrian hamsters. **a**) Viral RNA levels in the lungs of control (vehicle-treated), AT-527 -treated (at 150 or 250 mg/kg, BID) SARS-CoV-2–infected hamsters at day 3 post-infection (pi). Individual data and median values (indicated by bars) are presented and are expressed as log_{10} SARS-CoV-2 RNA copies per mg lung tissue. Data were analyzed with the Mann–Whitney U test (two-sided). Favipiravir is used as positive control (at 925 mg/kg/day). ns: not significant. **b**) Infectious viral loads in the lungs of control (vehicle-treated), AT-527-treated SARS-CoV-2-infected hamsters at day 3 pi (expressed as log_{10} $TCID_{50}$ per mg lung tissue). Individual data and median values (indicated by bars) are presented. Data were analyzed with the Mann–Whitney U test (two-sided). Favipiravir is used as positive control (at 925 mg/kg/day). ns: not significant, *P = 0.026, **P = 0.0087.

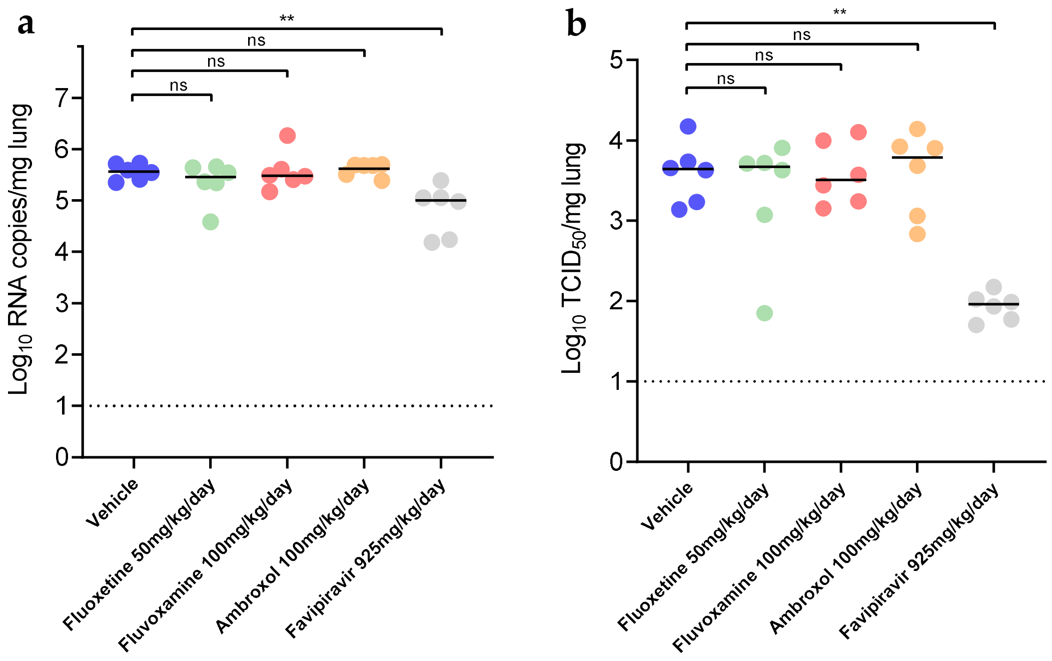

Figure S46. *In vivo* efficacy of fluoxetine, fluvoxamine and ambroxol in SARS-CoV-2 hamster model.

In vivo efficacy of fluoxetine, fluvoxamine and ambroxol against SARS-CoV-2 (Bav-Pat1) wild type strain in Syrian hamsters. **a**) Viral RNA levels in the lungs of control (vehicle-treated), fluoxetine, fluvoxamine and ambroxol -treated (at 50 and 100 mg/kg, QD and 50 mg/kg, BID) SARS-CoV-2–infected hamsters at day 3 post-infection (pi). Individual data and median values (indicated by bars) are presented and are expressed as $\log_{10}$ SARS-CoV-2 RNA copies per mg lung tissue. Data were analyzed with the Mann–Whitney U test (two-sided). Favipiravir is used as positive control (at 925 mg/kg/day). ns: not significant, **P = 0.043. **b**) Infectious viral loads in the lungs of control (vehicle-treated), fluoxetine, fluvoxamine and ambroxol -treated SARS-CoV-2-infected hamsters at day 3 pi (expressed as $\log_{10}$ $TCID_{50}$ per mg lung tissue). Individual data and median values (indicated by bars) are presented. Data were analyzed with the Mann–Whitney U test (two-sided). Favipiravir is used as positive control (at 925 mg/kg/day). ns: not significant, **P = 0.0022.

References

1. Jochmans, D.; Leyssen, P.; Neyts, J. A novel method for high-throughput screening to quantify antiviral activity against viruses that induce limited CPE. *J Virol Methods* **2012**, *183*, 176-179, doi:10.1016/j.jviromet.2012.04.011.
2. Do, T.N.D.; Donckers, K.; Vangeel, L.; Chatterjee, A.K.; Gallay, P.A.; Bobardt, M.D.; Bilello, J.P.; Cihlar, T.; De Jonghe, S.; Neyts, J.; et al. A robust SARS-CoV-2 replication model in primary human epithelial cells at the air liquid interface to assess antiviral agents. *Antiviral Res* **2021**, *192*, 105122, doi:10.1016/j.antiviral.2021.105122.
3. Touret, F.; Driouich, J.S.; Cochin, M.; Petit, P.R.; Gilles, M.; Barthélémy, K.; Moureau, G.; Mahon, F.X.; Malvy, D.; Solas, C.; et al. Preclinical evaluation of Imatinib does not support its use as an antiviral drug against SARS-CoV-2. *Antiviral Res* **2021**, *193*, 105137, doi:10.1016/j.antiviral.2021.105137.
4. Pizzorno, A.; Padey, B.; Julien, T.; Trouillet-Assant, S.; Traversier, A.; Errazuriz-Cerda, E.; Fouret, J.; Dubois, J.; Gaymard, A.; Lescure, F.X.; et al. Characterization and Treatment of SARS-CoV-2 in Nasal and Bronchial Human Airway Epithelia. *Cell Rep Med* **2020**, *1*, 100059, doi:10.1016/j.xcrm.2020.100059.
5. Gruber, A.D.; Firsching, T.C.; Trimpert, J.; Dietert, K. Hamster models of COVID-19 pneumonia reviewed: How human can they be? *Vet Pathol* **2022**, *59*, 528-545, doi:10.1177/03009858211057197.
6. Sia, S.F.; Yan, L.M.; Chin, A.W.H.; Fung, K.; Choy, K.T.; Wong, A.Y.L.; Kaewpreedee, P.; Perera, R.; Poon, L.L.M.; Nicholls, J.M.; et al. Pathogenesis and transmission of SARS-CoV-2 in golden hamsters. *Nature* **2020**, *583*, 834-838, doi:10.1038/s41586-020-2342-5.
7. Francis, M.E.; Goncin, U.; Kroeker, A.; Swan, C.; Ralph, R.; Lu, Y.; Etzioni, A.L.; Falzarano, D.; Gerdts, V.; Machtaler, S.; et al. SARS-CoV-2 infection in the Syrian hamster model causes inflammation as well as type I interferon dysregulation in both

respiratory and non-respiratory tissues including the heart and kidney. *PLoS Pathog* **2021**, *17*, e1009705, doi:10.1371/journal.ppat.1009705.
8. Sheahan, T.P.; Sims, A.C.; Zhou, S.; Graham, R.L.; Pruijssers, A.J.; Agostini, M.L.; Leist, S.R.; Schäfer, A.; Dinnon, K.H., 3rd; Stevens, L.J.; et al. An orally bioavailable broad-spectrum antiviral inhibits SARS-CoV-2 in human airway epithelial cell cultures and multiple coronaviruses in mice. *Sci Transl Med* **2020**, *12*, doi:10.1126/scitranslmed.abb5883.
9. Cox, R.M.; Wolf, J.D.; Plemper, R.K. Therapeutically administered ribonucleoside analogue MK-4482/EIDD-2801 blocks SARS-CoV-2 transmission in ferrets. *Nat Microbiol* **2021**, *6*, 11-18, doi:10.1038/s41564-020-00835-2.
10. Sheahan, T.P.; Sims, A.C.; Graham, R.L.; Menachery, V.D.; Gralinski, L.E.; Case, J.B.; Leist, S.R.; Pyrc, K.; Feng, J.Y.; Trantcheva, I.; et al. Broad-spectrum antiviral GS-5734 inhibits both epidemic and zoonotic coronaviruses. *Sci Transl Med* **2017**, *9*, doi:10.1126/scitranslmed.aal3653.
11. Pruijssers, A.J.; George, A.S.; Schäfer, A.; Leist, S.R.; Gralinksi, L.E.; Dinnon, K.H., 3rd; Yount, B.L.; Agostini, M.L.; Stevens, L.J.; Chappell, J.D.; et al. Remdesivir Inhibits SARS-CoV-2 in Human Lung Cells and Chimeric SARS-CoV Expressing the SARS-CoV-2 RNA Polymerase in Mice. *Cell Rep* **2020**, *32*, 107940, doi:10.1016/j.celrep.2020.107940.
12. Boudewijns, R.; Thibaut, H.J.; Kaptein, S.J.F.; Li, R.; Vergote, V.; Seldeslachts, L.; Van Weyenbergh, J.; De Keyzer, C.; Bervoets, L.; Sharma, S.; et al. STAT2 signaling restricts viral dissemination but drives severe pneumonia in SARS-CoV-2 infected hamsters. *Nat Commun* **2020**, *11*, 5838, doi:10.1038/s41467-020-19684-y.
13. Kaptein, S.J.F.; Jacobs, S.; Langendries, L.; Seldeslachts, L.; Ter Horst, S.; Liesenborghs, L.; Hens, B.; Vergote, V.; Heylen, E.; Barthelemy, K.; et al. Favipiravir at high doses has potent antiviral activity in SARS-CoV-2-infected hamsters, whereas hydroxychloroquine lacks activity. *Proc Natl Acad Sci U S A* **2020**, *117*, 26955-26965, doi:10.1073/pnas.2014441117.
14. Abdelnabi, R.; Boudewijns, R.; Foo, C.S.; Seldeslachts, L.; Sanchez-Felipe, L.; Zhang, X.; Delang, L.; Maes, P.; Kaptein, S.J.F.; Weynand, B.; et al. Comparing infectivity and virulence of emerging SARS-CoV-2 variants in Syrian hamsters. *EBioMedicine* **2021**, *68*, 103403, doi:10.1016/j.ebiom.2021.103403.
15. Cochin, M.; Luciani, L.; Touret, F.; Driouich, J.S.; Petit, P.R.; Moureau, G.; Baronti, C.; Laprie, C.; Thirion, L.; Maes, P.; et al. The SARS-CoV-2 Alpha variant exhibits comparable fitness to the D614G strain in a Syrian hamster model. *Commun Biol* **2022**, *5*, 225, doi:10.1038/s42003-022-03171-9.
16. Cochin, M.; Touret, F.; Driouich, J.S.; Moureau, G.; Petit, P.R.; Laprie, C.; Solas, C.; de Lamballerie, X.; Nougairède, A. Hydroxychloroquine and azithromycin used alone or combined are not effective against SARS-CoV-2 ex vivo and in a hamster model. *Antiviral Res* **2022**, *197*, 105212, doi:10.1016/j.antiviral.2021.105212.
17. Driouich, J.S.; Cochin, M.; Lingas, G.; Moureau, G.; Touret, F.; Petit, P.R.; Piorkowski, G.; Barthélémy, K.; Laprie, C.; Coutard, B.; et al. Favipiravir antiviral efficacy against SARS-CoV-2 in a hamster model. *Nat Commun* **2021**, *12*, 1735, doi:10.1038/s41467-021-21992-w.
18. Driouich, J.-S.; Cochin, M.; Touret, F.; Petit, P.-R.; Gilles, M.; Moureau, G.; Barthélémy, K.; Laprie, C.; Wattanakul, T.; Chotsiri, P.; et al. Pre-clinical evaluation of antiviral activity of nitazoxanide against Sars-CoV-2. *EBioMedicine* **2022,** 82:104148. doi: 10.1016/j.ebiom.2022.104148.
19. Savic, R.M.; Jonker, D.M.; Kerbusch, T.; Karlsson, M.O. Implementation of a transit compartment model for describing drug absorption in pharmacokinetic studies. *J Pharmacokinet Pharmacodyn* **2007**, *34*, 711-726, doi:10.1007/s10928-007-9066-0.
20. Dosne, A.G.; Bergstrand, M.; Karlsson, M.O. An automated sampling importance resampling procedure for estimating parameter uncertainty. *J Pharmacokinet Pharmacodyn* **2017**, *44*, 509-520, doi:10.1007/s10928-017-9542-0.
21. Wattanakul, T.; Chotsiri, P.; Scandale, I.; Hoglund, R.M.; Tarning, J. Pharmacometric approach to evaluate drug for potential repurposing as COVID-19 therapeutics. *Expert Rev Clin Pharmacol* **2022**, accepted.
22. Ferreira Sales-Medina, D.; Rodrigues Pinto Ferreira, L.; Romera, L.M.D.; Ribeiro Gonçalves, K.; Guido, R.V.C.; Courtemanche, G.; Buckeridge, M.S.; Durigon, E.L.; Moraes, C.B.; Freitas-Junior, L.H. Discovery of clinically approved drugs capable of inhibiting SARS-CoV-2 in vitro infection using a phenotypic screening strategy and network-analysis to predict their potential to treat covid-19. *bioRxiv* **2020**, doi:https://doi.org/10.1101/2020.07.09.196337.
23. Fintelman-Rodrigues, N.; Sacramento, C.Q.; Ribeiro Lima, C.; Souza da Silva, F.; Ferreira, A.C.; Mattos, M.; de Freitas, C.S.; Cardoso Soares, V.; da Silva Gomes Dias, S.; Temerozo, J.R.; et al. Atazanavir, Alone or in Combination with Ritonavir, Inhibits SARS-CoV-2 Replication and Proinflammatory Cytokine Production. *Antimicrob Agents Chemother* **2020**, *64*, doi:10.1128/aac.00825-20.
24. Yamamoto, N.; Matsuyama, S.; Hoshino, T.; Yamamoto, N. Nelfinavir inhibits replication of severe acute respiratory syndrome coronavirus 2 in vitro. *bioRxiv* **2020**, doi:10.1101/2020.04.06.026476.
25. Jeon, S.; Ko, M.; Lee, J.; Choi, I.; Byun, S.Y.; Park, S.; Shum, D.; Kim, S. Identification of Antiviral Drug Candidates against SARS-CoV-2 from FDA-Approved Drugs. *Antimicrob Agents Chemother* **2020**, *64*, doi:10.1128/AAC.00819-20.
26. Sacramento, C.Q.; Fintelman-Rodrigues, N.; Temerozo, J.R.; Da Silva, A.P.D.; Dias, S.; da Silva, C.D.S.; Ferreira, A.C.; Mattos, M.; Pao, C.R.R.; de Freitas, C.S.; et al. In vitro antiviral activity of the anti-HCV drugs daclatasvir and sofosbuvir against SARS-CoV-2, the aetiological agent of COVID-19. *J Antimicrob Chemother* **2021**, *76*, 1874-1885, doi:10.1093/jac/dkab072.
27. Dittmar, M.; Lee, J.S.; Whig, K.; Segrist, E.; Li, M.; Kamalia, B.; Castellana, L.; Ayyanathan, K.; Cardenas-Diaz, F.L.; Morrisey, E.E.; et al. Drug repurposing screens reveal cell-type-specific entry pathways and FDA-approved drugs active against SARS-Cov-2. *Cell Rep* **2021**, *35*, 108959, doi:10.1016/j.celrep.2021.108959.
28. Ko, M.; Jeon, S.; Ryu, W.S.; Kim, S. Comparative analysis of antiviral efficacy of FDA-approved drugs against SARS-CoV-2 in human lung cells. *J Med Virol* **2021**, *93*, 1403-1408, doi:10.1002/jmv.26397.

29. Si, L.; Bai, H.; Rodas, M.; Cao, W.; Oh, C.Y.; Jiang, A.; Moller, R.; Hoagland, D.; Oishi, K.; Horiuchi, S.; et al. A human-airway-on-a-chip for the rapid identification of candidate antiviral therapeutics and prophylactics. *Nat Biomed Eng* **2021**, *5*, 815-829, doi:10.1038/s41551-021-00718-9.
30. Weston, S.; Coleman, C.M.; Haupt, R.; Logue, J.; Matthews, K.; Li, Y.; Reyes, H.M.; Weiss, S.R.; Frieman, M.B. Broad Anti-coronavirus Activity of Food and Drug Administration-Approved Drugs against SARS-CoV-2 In Vitro and SARS-CoV In Vivo. *J Virol* **2020**, *94*, doi:10.1128/JVI.01218-20.
31. Choy, K.T.; Wong, A.Y.; Kaewpreedee, P.; Sia, S.F.; Chen, D.; Hui, K.P.Y.; Chu, D.K.W.; Chan, M.C.W.; Cheung, P.P.; Huang, X.; et al. Remdesivir, lopinavir, emetine, and homoharringtonine inhibit SARS-CoV-2 replication in vitro. *Antiviral Res* **2020**, *178*, 104786, doi:10.1016/j.antiviral.2020.104786.
32. Liu, S.; Lien, C.Z.; Selvaraj, P.; Wang, T.T. Evaluation of 19 antiviral drugs against SARS-CoV-2 Infection. *BioRxiv* **2020**.
33. Ohashi, H.; Watashi, K.; Saso, W.; Shionoya, K.; Iwanami, S.; Hirokawa, T.; Shirai, T.; Kanaya, S.; Ito, Y.; Kim, K.S.; et al. Potential anti-COVID-19 agents, cepharanthine and nelfinavir, and their usage for combination treatment. *iScience* **2021**, *24*, 102367, doi:10.1016/j.isci.2021.102367.
34. Shannon, A.; Selisko, B.; Le, N.T.; Huchting, J.; Touret, F.; Piorkowski, G.; Fattorini, V.; Ferron, F.; Decroly, E.; Meier, C.; et al. Rapid incorporation of Favipiravir by the fast and permissive viral RNA polymerase complex results in SARS-CoV-2 lethal mutagenesis. *Nat Commun* **2020**, *11*, 4682, doi:10.1038/s41467-020-18463-z.
35. Wang, M.; Cao, R.; Zhang, L.; Yang, X.; Liu, J.; Xu, M.; Shi, Z.; Hu, Z.; Zhong, W.; Xiao, G. Remdesivir and chloroquine effectively inhibit the recently emerged novel coronavirus (2019-nCoV) in vitro. *Cell Res* **2020**, *30*, 269-271, doi:10.1038/s41422-020-0282-0.
36. Zandi, K.; Amblard, F.; Musall, K.; Downs-Bowen, J.; Kleinbard, R.; Oo, A.; Cao, D.; Liang, B.; Russell, O.O.; McBrayer, T.; et al. Repurposing Nucleoside Analogs for Human Coronaviruses. *Antimicrob Agents Chemother* **2020**, *65*, doi:10.1128/AAC.01652-20.
37. Caly, L.; Druce, J.D.; Catton, M.G.; Jans, D.A.; Wagstaff, K.M. The FDA-approved drug ivermectin inhibits the replication of SARS-CoV-2 in vitro. *Antiviral Res* **2020**, *178*, 104787, doi:10.1016/j.antiviral.2020.104787.
38. Jeffreys, L.N.; Pennington, S.H.; Duggan, J.; Caygill, C.H.; Lopeman, R.C.; Breen, A.F.; Jinks, J.B.; Ardrey, A.; Donnellan, S.; Patterson, E.I.; et al. Remdesivir-ivermectin combination displays synergistic interaction with improved in vitro activity against SARS-CoV-2. *Int J Antimicrob Agents* **2022**, *59*, 106542, doi:10.1016/j.ijantimicag.2022.106542.
39. Yuan, S.; Yin, X.; Meng, X.; Chan, J.F.; Ye, Z.W.; Riva, L.; Pache, L.; Chan, C.C.; Lai, P.M.; Chan, C.C.; et al. Clofazimine broadly inhibits coronaviruses including SARS-CoV-2. *Nature* **2021**, *593*, 418-423, doi:10.1038/s41586-021-03431-4.
40. Yamamoto, N.; Yang, R.; Yoshinaka, Y.; Amari, S.; Nakano, T.; Cinatl, J.; Rabenau, H.; Doerr, H.W.; Hunsmann, G.; Otaka, A.; et al. HIV protease inhibitor nelfinavir inhibits replication of SARS-associated coronavirus. *Biochem Biophys Res Commun* **2004**, *318*, 719-725, doi:10.1016/j.bbrc.2004.04.083.
41. Xu, Z.; Yao, H.; Shen, J.; Wu, N.; Xu, Y.; Lu, X.; Zhu, W.; Li, L.-J. Nelfinavir Is Active Against SARS-CoV-2 in Vero E6 Cells. *ChemRxiv* **2020**.
42. Good, S.S.; Westover, J.; Jung, K.H.; Zhou, X.J.; Moussa, A.; La Colla, P.; Collu, G.; Canard, B.; Sommadossi, J.P. AT-527, a Double Prodrug of a Guanosine Nucleotide Analog, Is a Potent Inhibitor of SARS-CoV-2 In Vitro and a Promising Oral Antiviral for Treatment of COVID-19. *Antimicrob Agents Chemother* **2021**, *65*, doi:10.1128/AAC.02479-20.
43. Rogosnitzky, M.; Danks, R. Therapeutic potential of the biscoclaurine alkaloid, cepharanthine, for a range of clinical conditions. *Pharmacol Rep* **2011**, *63*, 337-347, doi:10.1016/s1734-1140(11)70500-x.
44. Zimniak, M.; Kirschner, L.; Hilpert, H.; Geiger, N.; Danov, O.; Oberwinkler, H.; Steinke, M.; Sewald, K.; Seibel, J.; Bodem, J. The serotonin reuptake inhibitor Fluoxetine inhibits SARS-CoV-2 in human lung tissue. *Sci Rep* **2021**, *11*, 5890, doi:10.1038/s41598-021-85049-0.
45. Schloer, S.; Brunotte, L.; Goretzko, J.; Mecate-Zambrano, A.; Korthals, N.; Gerke, V.; Ludwig, S.; Rescher, U. Targeting the endolysosomal host-SARS-CoV-2 interface by clinically licensed functional inhibitors of acid sphingomyelinase (FIASMA) including the antidepressant fluoxetine. *Emerg Microbes Infect* **2020**, *9*, 2245-2255, doi:10.1080/22221751.2020.1829082.
46. Fred, S.M.; Kuivanen, S.; Ugurlu, H.; Casarotto, P.C.; Levanov, L.; Saksela, K.; Vapalahti, O.; Castren, E. Antidepressant and Antipsychotic Drugs Reduce Viral Infection by SARS-CoV-2 and Fluoxetine Shows Antiviral Activity Against the Novel Variants in vitro. *Front Pharmacol* **2021**, *12*, 755600, doi:10.3389/fphar.2021.755600.
47. Olaleye, O.A.; Kaur, M.; Onyenaka, C.C. Ambroxol Hydrochloride Inhibits the Interaction between Severe Acute Respiratory Syndrome Coronavirus 2 Spike Protein's Receptor Binding Domain and Recombinant Human ACE2. *BioRxiv* **2020**.
48. Mahoney, M.; Damalanka, V.C.; Tartell, M.A.; Chung, D.H.; Lourenco, A.L.; Pwee, D.; Mayer Bridwell, A.E.; Hoffmann, M.; Voss, J.; Karmakar, P.; et al. A novel class of TMPRSS2 inhibitors potently block SARS-CoV-2 and MERS-CoV viral entry and protect human epithelial lung cells. *Proc Natl Acad Sci U S A* **2021**, *118*, doi:10.1073/pnas.2108728118.
49. Schultz, D.C.; Johnson, R.M.; Ayyanathan, K.; Miller, J.; Whig, K.; Kamalia, B.; Dittmar, M.; Weston, S.; Hammond, H.L.; Dillen, C.; et al. Pyrimidine inhibitors synergize with nucleoside analogues to block SARS-CoV-2. *Nature* **2022**, *604*, 134-140, doi:10.1038/s41586-022-04482-x.
50. Owen, D.R.; Allerton, C.M.N.; Anderson, A.S.; Aschenbrenner, L.; Avery, M.; Berritt, S.; Boras, B.; Cardin, R.D.; Carlo, A.; Coffman, K.J.; et al. An oral SARS-CoV-2 M(pro) inhibitor clinical candidate for the treatment of COVID-19. *Science* **2021**, *374*, 1586-1593, doi:10.1126/science.abl4784.
51. Xiong, R.; Zhang, L.; Li, S.; Sun, Y.; Ding, M.; Wang, Y.; Zhao, Y.; Wu, Y.; Shang, W.; Jiang, X.; et al. Novel and potent inhibitors targeting DHODH are broad-spectrum antivirals against RNA viruses including newly-emerged coronavirus SARS-CoV-2. *Protein Cell* **2020**, *11*, 723-739, doi:10.1007/s13238-020-00768-w.

52. Swaim, C.D.; Dwivedi, V.; Perng, Y.C.; Zhao, X.; Canadeo, L.A.; Harastani, H.H.; Darling, T.L.; Boon, A.C.M.; Lenschow, D.J.; Kulkarni, V.; et al. 6-Thioguanine blocks SARS-CoV-2 replication by inhibition of PLpro. *iScience* **2021**, *24*, 103213, doi:10.1016/j.isci.2021.103213.
53. McCoy, J.; Goren, A.; Cadegiani, F.A.; Vaño-Galván, S.; Kovacevic, M.; Situm, M.; Shapiro, J.; Sinclair, R.; Tosti, A.; Stanimirovic, A.; et al. Proxalutamide Reduces the Rate of Hospitalization for COVID-19 Male Outpatients: A Randomized Double-Blinded Placebo-Controlled Trial. *Front Med (Lausanne)* **2021**, *8*, 668698, doi:10.3389/fmed.2021.668698.
54. Murray, J.; Hogan, R.J.; Martin, D.E.; Blahunka, K.; Sancilio, F.D.; Balyan, R.; Lovern, M.; Still, R.; Tripp, R.A. Probenecid inhibits SARS-CoV-2 replication in vivo and in vitro. *Sci Rep* **2021**, *11*, 18085, doi:10.1038/s41598-021-97658-w.
55. Box, H.; Pennington, S.H.; Kijak, E.; Tatham, L.; Caygill, C.H.; Lopeman, R.C.; Jeffreys, L.N.; Herriott, J.; Sharp, J.; Neary, M.; et al. Lack of antiviral activity of probenecid in Vero E6 cells and Syrian golden hamsters: a need for better understanding of inter-lab differences in preclinical assays. *BioRxiv* **2022**.
56. Muturi, E.; Hong, W.; Li, J.; Yang, W.; He, J.; Wei, H.; Yang, H. Effects of simeprevir on the replication of SARS-CoV-2 in vitro and in transgenic hACE2 mice. *Int J Antimicrob Agents* **2022**, *59*, 106499, doi:10.1016/j.ijantimicag.2021.106499.
57. Abdelnabi, R.; Foo, C.S.; Kaptein, S.J.F.; Zhang, X.; Do, T.N.D.; Langendries, L.; Vangeel, L.; Breuer, J.; Pang, J.; Williams, R.; et al. The combined treatment of Molnupiravir and Favipiravir results in a potentiation of antiviral efficacy in a SARS-CoV-2 hamster infection model. *EBioMedicine* **2021**, *72*, 103595, doi:10.1016/j.ebiom.2021.103595.
58. Jan, J.T.; Cheng, T.R.; Juang, Y.P.; Ma, H.H.; Wu, Y.T.; Yang, W.B.; Cheng, C.W.; Chen, X.; Chou, T.H.; Shie, J.J.; et al. Identification of existing pharmaceuticals and herbal medicines as inhibitors of SARS-CoV-2 infection. *Proc Natl Acad Sci U S A* **2021**, *118*, doi:10.1073/pnas.2021579118.
59. Arevalo, A.P.; Pagotto, R.; Porfido, J.L.; Daghero, H.; Segovia, M.; Yamasaki, K.; Varela, B.; Hill, M.; Verdes, J.M.; Duhalde Vega, M.; et al. Ivermectin reduces in vivo coronavirus infection in a mouse experimental model. *Sci Rep* **2021**, *11*, 7132, doi:10.1038/s41598-021-86679-0.
60. Abdelnabi, R.; Foo, C.S.; Jochmans, D.; Vangeel, L.; De Jonghe, S.; Augustijns, P.; Mols, R.; Weynand, B.; Wattanakul, T.; Hoglund, R.M.; et al. The oral protease inhibitor (PF-07321332) protects Syrian hamsters against infection with SARS-CoV-2 variants of concern. *Nat Commun* **2022**, *13*, 719, doi:10.1038/s41467-022-28354-0.
61. Pussard, E.; Verdier, F. Antimalarial 4-aminoquinolines: mode of action and pharmacokinetics. *Fundam Clin Pharmacol* **1994**, *8*, 1-17, doi:10.1111/j.1472-8206.1994.tb00774.x.
62. Atazanavir PK fact sheet. Available from: https://liverpool-hiv-hep.s3.amazonaws.com/fact_sheets/pdfs/000/000/087/original/HIV_FactSheet_ATV_2016_Mar.pdf (last accessed July 2022).
63. Gandhi, Y.; Eley, T.; Fura, A.; Li, W.; Bertz, R.J.; Garimella, T. Daclatasvir: A Review of Preclinical and Clinical Pharmacokinetics. *Clin Pharmacokinet* **2018**, *57*, 911-928, doi:10.1007/s40262-017-0624-3.
64. Hayden, F.G.; Shindo, N. Influenza virus polymerase inhibitors in clinical development. *Current opinion in infectious diseases* **2019**, *32*, 176-186, doi:10.1097/qco.0000000000000532.
65. González Canga, A.; Sahagún Prieto, A.M.; Diez Liébana, M.J.; Fernández Martínez, N.; Sierra Vega, M.; García Vieitez, J.J. The pharmacokinetics and interactions of ivermectin in humans--a mini-review. *The AAPS journal* **2008**, *10*, 42-46, doi:10.1208/s12248-007-9000-9.
66. Romark Pharmaceuticals, Alinia®, Product monograph, available from: https://www.accessdata.fda.gov/drugsatfda_docs/label/2005/021818lbl.pdf. **2005**.
67. Cada, D.J.; Cong, J.; Baker, D.E. Sofosbuvir. *Hospital pharmacy* **2014**, *49*, 466-478, doi:10.1310/hpj4905-466.
68. Lee, H.J.; Joung, S.K.; Kim, Y.G.; Yoo, J.Y.; Han, S.B. Bioequivalence assessment of ambroxol tablet after a single oral dose administration to healthy male volunteers. *Pharmacological research* **2004**, *49*, 93-98, doi:10.1016/j.phrs.2003.07.011.
69. Tarning, J.; Chotsiri, P.; Jullien, V.; Rijken, M.J.; Bergstrand, M.; Cammas, M.; McGready, R.; Singhasivanon, P.; Day, N.P.; White, N.J.; et al. Population pharmacokinetic and pharmacodynamic modeling of amodiaquine and desethylamodiaquine in women with Plasmodium vivax malaria during and after pregnancy. *Antimicrob Agents Chemother* **2012**, *56*, 5764-5773, doi:10.1128/aac.01242-12.
70. Punyawudho, B.; Thammajaruk, N.; Ruxrungtham, K.; Avihingsanon, A. Population pharmacokinetics and dose optimisation of ritonavir-boosted atazanavir in Thai HIV-infected patients. *Int J Antimicrob Agents* **2017**, *49*, 327-332, doi:10.1016/j.ijantimicag.2016.11.019.
71. Hao, G.; Liang, H.; Li, Y.; Li, H.; Gao, H.; Liu, G.; Liu, Z. Simple, sensitive and rapid HPLC-MS/MS method for the determination of cepharanthine in human plasma. *Journal of chromatography. B, Analytical technologies in the biomedical and life sciences* **2010**, *878*, 2923-2927, doi:10.1016/j.jchromb.2010.08.026.
72. Yasuda, K.; Moro, M.; Akasu, M.; Ohnishi, A. Pharmacokinetic disposition of cepharanthin following single and multiple intravenous doses in healthy subjects. *Jpn J Clin Pharmacol Ther* **1989**, *20*, 741-749, doi:10.3999/jscpt.20.741.
73. Yasuda, K.; Moro, M.; Ohnishi, A.; Akasu, M.; Shishido, A.; Tsunoo, M. Pharmacokinetic study of cepharanthin following single oral doses in healthy subjects. *Jpn J Clin Pharmacol Ther* **1989**, *20*, 735-740, doi:10.3999/jscpt.20.735.
74. Faraj, A.; Svensson, R.J.; Diacon, A.H.; Simonsson, U.S.H. Drug Effect of Clofazimine on Persisters Explains an Unexpected Increase in Bacterial Load in Patients. *Antimicrob Agents Chemother* **2020**, *64*, doi:10.1128/aac.01905-19.
75. Chan, P.; Li, H.; Zhu, L.; Bifano, M.; Eley, T.; Osawa, M.; Ueno, T.; Hughes, E.; Bertz, R.; Garimella, T.; et al. Population Pharmacokinetic Analysis of Daclatasvir in Subjects with Chronic Hepatitis C Virus Infection. *Clin Pharmacokinet* **2017**, *56*, 1173-1183, doi:10.1007/s40262-016-0504-2.

76. Wang, Y.; Zhong, W.; Salam, A.; Tarning, J.; Zhan, Q.; Huang, J.A.; Weng, H.; Bai, C.; Ren, Y.; Yamada, K.; et al. Phase 2a, open-label, dose-escalating, multi-center pharmacokinetic study of favipiravir (T-705) in combination with oseltamivir in patients with severe influenza. *EBioMedicine* **2020**, *62*, 103125, doi:10.1016/j.ebiom.2020.103125.
77. Sagahón-Azúa, J.; Medellín-Garibay, S.E.; Chávez-Castillo, C.E.; González-Salinas, C.G.; Milán-Segovia, R.D.C.; Romano-Moreno, S. Factors associated with fluoxetine and norfluoxetine plasma concentrations and clinical response in Mexican patients with mental disorders. *Pharmacology research & perspectives* **2021**, *9*, e00864, doi:10.1002/prp2.864.
78. Orlando, R.; De Martin, S.; Andrighetto, L.; Floreani, M.; Palatini, P. Fluvoxamine pharmacokinetics in healthy elderly subjects and elderly patients with chronic heart failure. *Br J Clin Pharmacol* **2010**, *69*, 279-286, doi:10.1111/j.1365-2125.2009.03587.x.
79. Kobylinski, K.C.; Ubalee, R.; Ponlawat, A.; Nitatsukprasert, C.; Phasomkulsolsil, S.; Wattanakul, T.; Tarning, J.; Na-Bangchang, K.; McCardle, P.W.; Davidson, S.A.; et al. Ivermectin susceptibility and sporontocidal effect in Greater Mekong Subregion Anopheles. *Malaria journal* **2017**, *16*, 280, doi:10.1186/s12936-017-1923-8.
80. Painter, W.P.; Holman, W.; Bush, J.A.; Almazedi, F.; Malik, H.; Eraut, N.; Morin, M.J.; Szewczyk, L.J.; Painter, G.R. Human Safety, Tolerability, and Pharmacokinetics of Molnupiravir, a Novel Broad-Spectrum Oral Antiviral Agent with Activity Against SARS-CoV-2. *Antimicrob Agents Chemother* **2021**, *65*, doi:10.1128/aac.02428-20.
81. Hirt, D.; Treluyer, J.M.; Jullien, V.; Firtion, G.; Chappuy, H.; Rey, E.; Pons, G.; Mandelbrot, L.; Urien, S. Pregnancy-related effects on nelfinavir-M8 pharmacokinetics: a population study with 133 women. *Antimicrob Agents Chemother* **2006**, *50*, 2079-2086, doi:10.1128/aac.01596-05.
82. Rajoli, R.K.R.; Pertinez, H.; Arshad, U.; Box, H.; Tatham, L.; Curley, P.; Neary, M.; Sharp, J.; Liptrott, N.J.; Valentijn, A.; et al. Dose prediction for repurposing nitazoxanide in SARS-CoV-2 treatment or chemoprophylaxis. *Br J Clin Pharmacol* **2021**, *87*, 2078-2088, doi:10.1111/bcp.14619.
83. Balderas-Acata, J.; Bueno, E.; Pérez-Becerril, F.; Espinosa-Martínez, C.; Burkefraga, V.; González-de la Parra, M. Bioavailability of Two Oral-Suspension Formulations of a Single Dose of Nitazoxanide 500 mg: An Open-Label, Randomized-Sequence, Two-Period Crossover, Comparison in Healthy Fasted Mexican Adult Volunteers. *Journal of Bioequivalence & Bioavailability* **2011**, *03*, doi:10.4172/jbb.1000056.
84. Jin, F.; Kirby, B.; Gao, Y.; Kearney, B.; Mathias, A. Population Pharmacokinetic Modeling of Sofosbuvir, an NS5B Polymerase Inhibitor, and Its Metabolites in Patients With Hepatitis C Virus Infection, Poster presentation at PAGE-meeting, 2015, Hersonissos, Crete, Greece https://www.page-meeting.org/pdf_assets/3129-PAGE%20poster.pdf.
85. Rogosnitzky, M.; Okediji, P.; Koman, I. Cepharanthine: a review of the antiviral potential of a Japanese-approved alopecia drug in COVID-19. *Pharmacol Rep* **2020**, *72*, 1509-1516, doi:10.1007/s43440-020-00132-z.
